# A genetic screen for modifiers of *Drosophila *caspase Dcp-1 reveals caspase involvement in autophagy and novel caspase-related genes

**DOI:** 10.1186/1471-2121-11-9

**Published:** 2010-01-25

**Authors:** Young-Il Kim, Taewoo Ryu, Judong Lee, Young-Shin Heo, Joohong Ahnn, Seung-Jae Lee, OokJoon Yoo

**Affiliations:** 1 Bio Medical Research Center, Department of Biological Science, KAIST, 373-1, 305-701, Daejeon, Korea; 2 Department of Life Science, College of Natural Science, Hanyang University, 133-791, Seoul, Korea; 3 Division of Molecular and Life Sciences, School of Interdisciplinary Bioscience and Bioengineering, World Class University Division of IT Convergence Engineering, Pohang University of Science and Technology, 790-784, Pohang, Korea

## Abstract

**Background:**

Caspases are cysteine proteases with essential functions in the apoptotic pathway; their proteolytic activity toward various substrates is associated with the morphological changes of cells. Recent reports have described non-apoptotic functions of caspases, including autophagy. In this report, we searched for novel modifiers of the phenotype of *Dcp-1 *gain-of-function (GF) animals by screening promoter element- inserted *Drosophila melanogaster *lines (EP lines).

**Results:**

We screened ~15,000 EP lines and identified 72 *Dcp-1*-interacting genes that were classified into 10 groups based on their functions and pathways: 4 apoptosis signaling genes, 10 autophagy genes, 5 insulin/IGF and TOR signaling pathway genes, 6 MAP kinase and JNK signaling pathway genes, 4 ecdysone signaling genes, 6 ubiquitination genes, 11 various developmental signaling genes, 12 transcription factors, 3 translation factors, and 11 other unclassified genes including 5 functionally undefined genes. Among them, insulin/IGF and TOR signaling pathway, MAP kinase and JNK signaling pathway, and ecdysone signaling are known to be involved in autophagy. Together with the identification of autophagy genes, the results of our screen suggest that autophagy counteracts Dcp-1-induced apoptosis. Consistent with this idea, we show that expression of eGFP-Atg5 rescued the eye phenotype caused by *Dcp-1 *GF. Paradoxically, we found that over-expression of full-length *Dcp-1 *induced autophagy, as Atg8b-GFP, an indicator of autophagy, was increased in the eye imaginal discs and in the S2 cell line. Taken together, these data suggest that autophagy suppresses Dcp-1-mediated apoptotic cell death, whereas Dcp-1 positively regulates autophagy, possibly through feedback regulation.

**Conclusions:**

We identified a number of *Dcp-1 *modifiers that genetically interact with Dcp-1-induced cell death. Our results showing that *Dcp-1 *and autophagy-related genes influence each other will aid future investigations of the complicated relationships between apoptosis and autophagy.

## Background

Apoptosis, or programmed cell death, is an evolutionarily conserved, genetically regulated process, whereby cells that are no longer needed undergo self-destruction through the activation of a cell suicide program [1,2]. This cell death program is associated with characteristic morphological alterations, such as condensation of the nucleus and cytoplasm, fragmentation of nuclear DNA, reorganization of the cytoskeleton, and reduction of the cell into apoptotic bodies that can be phagocytosed by neighbouring epithelial cells or phagocytes [1,3].

Autophagy is also an evolutionarily conserved mechanism that degrades unnecessary long-lived proteins and organelles. During autophagy, cellular components are sequestered by double-membrane structures called autophagosomes. These autophagosomes then fuse with lysosomes to form autolysosomes, where degradation occurs [4]. The autophagy acts as a cellular response against extracellular stresses, such as nutrient starvation, hypoxia, and overcrowding and against intracellar stresses, such as formation of damaged or redundant organelles and cytoplasmic components [4]. Even though autophagy can induce a cell-survival response to some conditions, autophagic structures, especially the autophagic vacuoles, are associated with cell death. This cell death phenomenon is classified as type II cell death and called autophagic cell death. In *Drosophila*, loss of function of the *Atg *genes leads to lethality in the transition from the larval to pupal stages, because autophagic cell death is essential for puparium formation [4].

The caspases are a family of ubiquitously expressed cysteine proteases whose prototypic member is the *Caenorhabditis elegans *death effector, CED-3 [5]. Activation of caspases typically leads to the selective cleavage of a restricted set of target proteins, generally resulting in inactivation of the target proteins. Normally present in cells as inactive precursors, caspases are proteolytically activated following upstream pro-apoptotic signals. Activated caspases cleave their substrates at an aspartic acid residue, and substrate specificity is determined by a four-residue motif N-terminal to the cleavage site [6,7]. The "initiator" caspases primarily activate the downstream "effector" caspases whose proteolytic activity is directed toward the deconstruction of the cellular machinery during apoptosis.

In *Drosophila*, a normally functioning apoptotic pathway depends critically on caspases, seven of which have been identified in the *Drosophila melanogaster *genome [8,9]. These *Drosophila *caspase genes include three initiators and four effectors. The initiators are *Dronc *(Drosophila Nedd-2-like caspase), *Dredd *(Death-related ced-3/Nedd2-like), and *strica/dream*. The effectors are *Dcp-1 *(Death caspase-1), *Drice *(Drosophila ice), *Damm*, and *decay *(Death executioner caspase related to Apopain/Yama) [10-13]. These caspases are expected to have functions that lead to apoptosis. Many recent reports have described non-apoptotic functions of caspases, such as the cell proliferation function of *Dronc *[14,15], spermatid individualization by *Drice *[14,16], and activation of the *Drosophila *immune system, toll receptor signaling, by *Dredd *[14].

Dcp-1 proteins cleave cysteine protease substrates and are important for development and oogenesis [13,17]. *Dcp-1 *deletion mutants display a lack of germline cell death phenotype during mid-stage oogenesis in response to nutrient deprivation [18], whereas in normal flies, cell death occurs during mid-stage oogenesis under nutrient-deprived conditions (stage 7 to 8) [3,19-21]. In contrast, over-expression of a single copy of the truncated N-terminal region of Dcp-1 (constitutively active Dcp-1), specifically in the eye using the *Glass *Multimer Reporter (GMR) promoter, results in a slightly rough and reduced pigment eye phenotype [22]. In addition to the critical roles of Dcp-1 and caspase 3 in apoptosis, recent studies in mammals and flies suggest that these caspases have many important non-apoptotic roles [13,23], although how these caspases act in these non-apoptotic responses are incompletely understood.

We hypothesized the existence of unknown effectors for Dcp-1. Identification and characterization of such proteins would allow us to better understand apoptotic pathways and Dcp-1-related non-apoptotic pathways. Since a large-scale genetic screen to identify components of the Dcp-1 pathway had not been preformed, either *in vivo *or *in vitro*, we screened ~15,000 GenExel EP fly lines. Interestingly, we noticed that autophagy-related genes specifically suppressed the rough eye phenotype caused by *Dcp-1 *expression. In addition to eight autophagy genes and two genes reported to be related to autophagy, we identified five Insulin/IGF and TOR signaling genes, six MAP kinase and Jun N-terminal kinase (JNK) signaling, four Ecdysone genes, and others (Additional file 1). There were several interesting novel genes among the 72 *Dcp-1 *genetic interactors. The identification of many new *Dcp-1*-interacting genes will help clarify the molecular mechanisms by which *Dcp-1 *regulates apoptosis and other non-apoptotic cellular processes. In addition, our findings that autophagy genes influence the roles of the Dcp-1 caspase in autophagy suggest a relationship between autophagy and apoptosis. Finally, our findings that signaling pathways such as MAP kinase, JNK, and ecdysone signaling regulate Dcp-1 indicate that there are other regulatory pathways for caspase functions.

## Results

### Phenotypes caused by ectopic expression of *Drosophila *caspases

**Figure 1:**
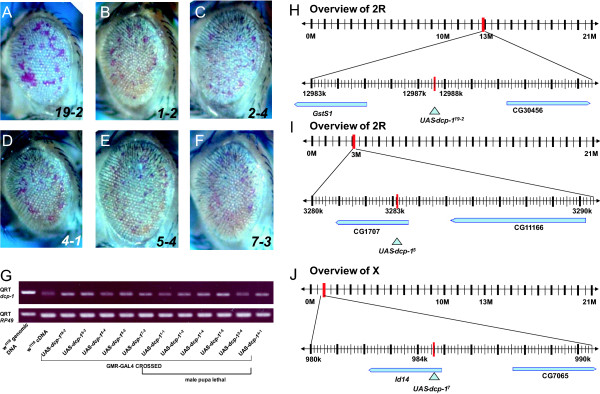
***Dcp-1 *fly lines used for screening**. (A) GMR-*GAL4*/UAS-*Dcp-1*^19-2 ^eyes exhibited a disordered rough eye phenotype with decreased pigmentation. (B, C, D, E, F) GMR-*GAL4*/UAS-*Dcp-1*^1-2^, GMR-*GAL4*/UAS-*Dcp-1*^2-4^, GMR-*GAL4*/UAS-*Dcp-1*^4-1^, GMR-*GAL4*/UAS-*Dcp-1*^5-4^, and GMR-*GAL4*/UAS-*Dcp-1*^7-3 ^eyes exhibited a disordered rough eye phenotype and pigment loss. (G) Lines derived from UAS-*Dcp-1*^1^, UAS-*Dcp-1*^2^, and UAS-*Dcp-1*^4 ^showed a male pupal lethality phenotype when crossed with GMR-*GAL4*. (H) UAS-*Dcp-1*^19-2 ^was inserted in 2R at position 12987659 between GstS1 and CG30456. (I) UAS-*Dcp-1*^5 ^was inserted in 2R at position 3283377 in the 5' region of CG1707. (J) UAS-*Dcp-1*^7 ^was inserted in X at position 9029070 in the 5' region of Id14.

**Figure 2:**
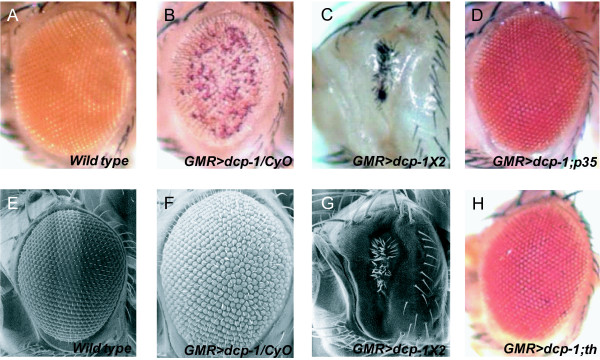
**Eye phenotype of *Dcp-1 *over-expressing flies**. (A and E) The eyes of wild-type flies. (B and F) The eyes of flies carrying one copy of *Dcp-1 *GF appeared small and rough with reduced pigmentation. (C and G) Flies carrying two copies of *Dcp-1 *GF have a dramatically stronger disrupted eye phenotype. Ommatidia and pigment spots are almost absent. (E, F, and G) Scanning electron micrographs of the eyes in panels A, B and C, respectively. (D and H) *Dcp-1 *GF phenotype was rescued by co-expression with *p*35 and *thread*.

To identify genes that interact with fly *Dcp-1*, we first generated transgenic flies that over-express full-length *Dcp-1 *using the Upstream Activation Sequence (UAS)/GAL4 system. We obtained 49 stable lines by microinjecting the UAS-*Dcp-1 *construct in *w*^1118 ^embryos. Forty out of 49 fly lines carrying this construct showed a small, slightly rough and reduced eye pigment phenotype when expressed with the eye-specific GMR-*GAL4 *drivers (Figure 1A-F). Among the UAS-*Dcp-1 *lines we generated, we chose to use UAS-*Dcp-1*^19-2 ^flies in this study for several reasons: First, we found that these flies did not exhibit a lethal phenotype. Second, the UAS-*Dcp-1 *was inserted between *GstS1 *and CG30456 without disrupting any flanking genes (Figure 1H). We found that in some other lines the UAS-*Dcp-1 *construct was inserted into exon regions of genes (Figure 1I and 1J). In addition, some transgenic flies showed a male pupal lethal phenotype when induced by GMR-*GAL4 *(Figure 1G). Lastly, since the GMR-*GAL4*/UAS-*Dcp-1*^19-2 ^flies displayed a modest rough eye phenotype, we reasoned that this fly line was suitable for identifying both suppressors and enhancers of *Dcp-1 *from our modifier screen. The eye phenotype that we observed for the GMR-*GAL4*, UAS-*Dcp-1 *flies was similar to a previous study in which flies that express one copy each of GMR-ΔN-*Dcp-1 *and GMR-fl-*Dcp-1 *(full-length *Dcp-1*) showed a faintly-colored and ablated eye phenotype [22]. The intermediate phenotype caused by one copy of GMR-*GAL4 *and one copy of UAS-*Dcp-1 *makes these flies very useful for screening for modifiers (Figure 2B and 2F). Flies carrying two copies of GMR-*GAL4 *and UAS-*Dcp-1 *displayed a much more severe eye phenotype, in general agreement with the phenotype from a previous study using two copies of GMR-ΔN-*Dcp-1 *(Figure 2C and 2G). These flies were semi-lethal and had a short life span. As positive controls for the suppressors of *Dcp-1 *over-expressing flies, we expressed caspase inhibitor *p35 *and Drosophila inhibitor of apoptosis (Diap1; *thread, th*). The eye phenotype caused by GMR-*GAL4*, UAS-*Dcp-1*^19-2 ^(hereafter abbreviated as *Dcp-1 *GF (gain of function); GMR-*GAL4 *and UAS-*Dcp-1*^19-2 ^are linked on one chromosome) was completely rescued by the expression of the caspase inhibitors (Figure 2D and 2H).

### *Dcp-1 *modifier screen

We screened ~15,000 EP lines obtained from the Szeged Stock Center, GenExel, and cell signaling pathway gene lines from the Indiana University Bloomington Stock Center and Dr. J. Chung's laboratory (KAIST, South Korea). Among them, 414 transgenic flies showed suppression or enhancement of the rough eye phenotype caused by the *Dcp-1 *GF. False positives were excluded by comparing these lines with the lines induced by GMR-*GAL4*- lines without UAS-*Dcp-1*. We confirmed that 85 alleles corresponding to 72 genes showed specific genetic interaction with *Dcp-1 *(Additional file 1). In addition, we verified that the *GAL4 *mRNA expression was constant even when *Dcp-1 *GF was crossed with representative alleles, *Atg1*^*EP*(*G*13748) ^and *Atg6*^*EP*(*G*6854) ^(Additional file 2). We also confirmed that EP insertions induced the genes placed down-stream by the GMR-*GAL4 *while not affecting the expression of flanking genes placed at a distance: the expression level of *CG10969*, down-stream gene of *Atg1*, was not affected by EP inserted up-stream of *Atg1 *gene (Additional file 3) and the expression level of *Tango5*, down-stream gene of *Atg8a*, was not affected by EP inserted up-stream of *Atg8a *gene (Additional file 4).

### Apoptotic cell death pathway

We identified three genes that are known to regulate apoptotic cell death. Expression of *Diap1 *suppressed the eye phenotype, whereas EP lines of effete (*eff) *and *faf *enhanced the rough eye phenotype (Additional file 1A). These results are consistent with the previous reports that Diap1 is a caspase inhibitor and that *eff *and *faf *enhance apoptotic cell death [24,25]. The fact that we recovered apoptosis-modulating genes with the expected direction shows the efficacy of our genetic screen.

### Autophagy-related genes

**Figure 3:**
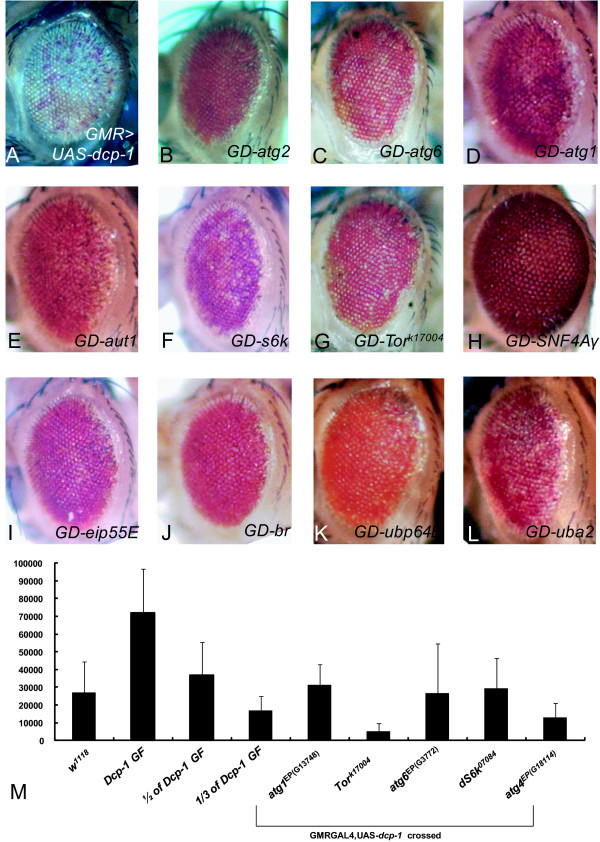
**Suppressors of the *Dcp-1 *GF eye phenotype**. (A) *Dcp-1 *GF, The *Dcp-1 *eye phenotype of flies with a single copy of *Dcp-1 *GF was suppressed by: (B) *Atg2*^*EP*(*G*6691)^, (C) *Atg6*^*EP*(*G*6854)^, (D) *Atg1*^*EP*(*G*13748)^, (E) *Aut1*^*EP*(*G*3894)^, (F) UAS-*dS6k*, (G) *Tor*^*k*17004^, (H) *SNF4Aγ*^*EP*(*GX*6409)^, (I) *Eip55E*^*EP*(*G*13564)^, (J) *br*^*EP*(*G*10174)^, (K) *Ubp64E*^*EP*(*G*5032)^, and (L) *Uba2*^*EP*(*G*4384)^. Each panel shows the effects of each gene in the *Dcp-1 *GF background. GD- indicates DCP-1 expression with each transgenes by crossing with *Dcp-1 *GF. (M) Caspase activity was determined with lysate from heads of each virgin flies from *w*^1118^, GMR-*GAL4*;UAS-*Dcp-1 *and GMR-*GAL4*;UAS-*Dcp-1 *which were crossed with autophagy related lines. Samples that provided statistically significant reduced activity in at least three independent experiments are shown (*, P < 0.05; **, P < 0.1 *t *test). RLU, relative light unit. The error bars represent the standard deviation of amount of RLU (B, C, D and E).

**Figure 4:**
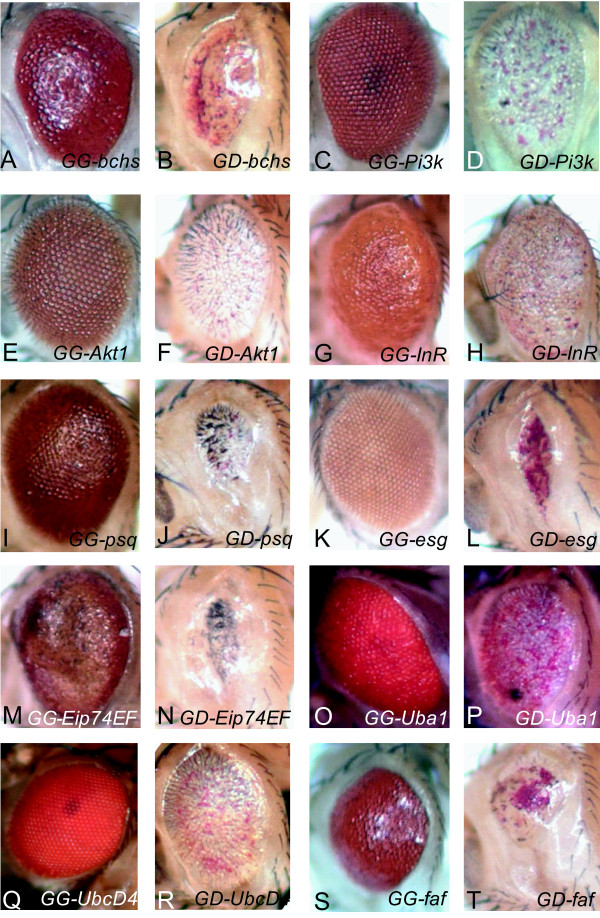
**Enhancers of the *Dcp-1 *GF eye phenotype**. The *Dcp-1 *eye phenotype of flies with a single copy of the *Dcp-1 *GF transgene was enhanced by (A, B) *bchs*, (C, D) *Pi3k*, (E, F) *Akt1*, (G, H) *InR*, (I, J) *psq*, (K, L) *esg*, (M, N) *Eip74EF*, (O, P) *Uba1*, (Q, R) *UbcD4*, and (S, T) *faf*. The left image of each pair (A, C, E, G, I, K, M, O, Q and S) shows the effect of an EP line crossed with the GMR-*GAL4 *driver alone. The right images (B, D, F, H, J, L, N, P, R and T) show the corresponding EP line crossed with *Dcp-1 *GF/*CyO*. GG- indicates transgenes that induced by GMR-*GAL4 *only. GD- indicates expression of each transgenes with DCP-1 by crossing with *Dcp-1 *GF.

Three autophagy-related genes, *Aut1, SNF4/*AMP-activated protein kinase γ subunit (SNF4Aγ), and Blue cheese (*Bchs*), were identified as genetic modifiers from unbiased screening: expression of *Aut1 *and SNF4Aγ suppressed the rough eye phenotype caused by *Dcp-1 *expression, whereas *Bchs *expression enhanced this phenotype, (Figure 3E, H and 4A and 4B, respectively). Aut1 is an important regulator of autophagy that is required for modification of Atg8 (Autophagy-specific gene 8) in a ubiquitin conjugation-like manner [26,27]. *Aut1 *loss of function mutant larvae fail to induce autophagy in the fat body before puparium stage and die during metamorphosis [26]. Recently, SNF4Aγ function in autophagosome formation during larval metamorphosis was revealed through a combination of *Drosophila *mutational and RNAi studies [28]. We found that eye-specific expression of SNF4Aγ suppressed the rough eye phenotype in the *Dcp-1 *GF flies (Figure 3H). It has been shown that SNF4Aγ induces developmental and stress-mediated autophagy [28]. Thus, our data suggest that autophagy induced by SNF4Aγ over-expression negatively modulates the *Dcp-1*-mediated cell death phenotype. *Bchs *is a *Drosophila *homolog of human Alfy (autophagy-linked FYVE protein), which may serve as a scaffold protein to promote autophagosome-related vesicle trafficking to lysosomes [29-31]. Since we identified three autophagy-related genes from our primary screen and since EP lines of *Aut1*, SNF4Aγ, and *Bchs *resulted in opposite phenotypes, we examined the EP lines for other autophagy-specific genes, including *Atg1*, *Atg2*, *Atg4*, *Atg6*, *Atg7, Atg8a*, and *Atg18 *(Additional file 1B). We found that 10 of 40 EP alleles for autophagy-specific genes partially recovered the disordered ommatidia and reduced eye pigment that was caused by *Dcp-1 *GF, as shown by introduction of *Atg2*^*EP*(*G*6691)^, *Atg6*^*EP*(*G*6654 and *G*3772)^, and *Atg1*^*EP*(*G*13748) ^(Figure 3BD). Atg1, whose activity is regulated by Tor (Target of rapamycin), initiates the induction of autophagy. Atg2 is important for retrieval of autophagic proteins and vesicles by interacting with membrane protein Atg9 during autophagosome formation. Atg6 plays a role in nucleation of the autophagic vesicles by formation of Class III PI3K complexes [4,32,33]. The general process of autophagy is considered to be a survival mechanism against cell death [34]. These data suggest that important components of autophagy generally counteract the cell death caused by *Dcp-1*over-expression. Consistent with this idea, we found that a *Bchs*^*EP*(*G*2362) ^line can suppress the rough eye phenotype in an allele-specific manner (see Additional file 1B and Discussion).

### Genetic interaction between the insulin-IGF pathway or TOR pathway and *Dcp-1*

In our screen, we identified a set of genes in the insulin-IGF signaling pathway, including *InR *(insulin receptor), *Pi3k*, *Akt1*, *S6k *(ribosomal subunit S6 kinase), *Tor *(Target of Rapamycin), and *Pten *(Additional file 1C). Insulin-IGF signaling is initiated by the activation of *InR*, which leads to the activation of a downstream kinase cascade including Pi3k and Akt1. On the other hand, PTEN phosphatase inhibits this signaling by dephosphorylating 3-phosphoinositides (PI(3)Ps), the product of PI3K [35]. Tor is an important regulator of nutrient responses, cellular growth, and protein synthesis [36]. Tor regulates cellular nutrients by insulin or the AMP: ATP ratio. Tor is activated by the insulin/IGF signaling through the IGF, Akt1, TSC1/2 and Rheb or by the LKB1 and AMPK signaling pathway through LKB1, AMPK, TSC1/2 and Rheb. The signals from these two pathways converge on TSC1/2, Rheb, and Tor [37]. One of the important downstream targets of Tor is *S6k*, which is an important regulator of protein translation [38-40]. We found that the rough eye phenotype caused by *Dcp-1 *GF was exacerbated by over-expression of *InR*, *Pi3k*, *Akt1, Pten*, or *Tor *(Figure 3C-H). The *Tor *hypomorphic mutant *Tor*^*k*17004^, which shows a more moderate phenotype than *Tor *null mutant *Tor*^*ΔP *^[41], recovered the *Dcp-1 *GF phenotype (Figure 3G).

We also identified several genes in the TOR pathway from our screen (Additional file 1C). In addition to the main role of S6k in the regulation of cell size [42], recent reports showed that S6k mediates many other physological processes, such as larval feeding behaviour [43], adult lifespan [44], and autophagy [45]. The progeny from the cross of *Dcp-1 *GF with the UAS-*dS6k *showed a suppressed rough eye phenotype (Figure 3F and Additional file 1C). On the other hand, Pten is known to antagonize Pi3k signaling. However, our data showed that the eye phenotype of *Dcp-1 *GF was exacerbated by co-expression of UAS-*Pten*^*ff*20.2^, the line that over-expresses wild type *Pten*. Although this result seems to contradict the enhancement of the rough eye phenotype caused by Pi3k expression, recent studies have shown that *Pten *over-expression induces apoptosis in a cell context-dependent manner even though Pten functions to alleviate the effect of Insulin/IGF signaling [46]. Thus, it is possible that Pten may reinforce the apoptotic effect of Dcp-1 by acting in some other signaling pathways that are distinct from the insulin Pi3k signaling and may worsen the rough eye phenotype caused by *Dcp-1 *GF.

Overall, nine autophagy-specific genes as well as Tor, which is a well-known regulator of autophagy, modified *Dcp-1 *GF. The identification of these genes as rescuers of the *Dcp-1 *phenotype raised questions about the effect of autophagy genes on Dcp-1 caspase function. Thus, we performed caspase assays using the autophagy strains that over-expressed *Dcp-1*. We confirmed that over-expression of *Dcp-1 *strongly increased caspase activity in *Dcp-1 *GF animals (Figure 3M). The increased caspase activity induced by *Dcp-1 *over-expression was reduced by the expression of autophagy genes in the co-heterozygotic lines with *Atg1*, *Tor*^*k*17004^, *Atg6*, *dS6k*, and *Atg4 *(Figure 3M).

### The MAPK and JNK pathway

The JNK pathway is a well known signaling cascade that regulates apoptosis. We found that the rough eye phenotype of *Dcp-1 *GF was also severely affected by the expression of many components in the MAPK and JNK pathways, such as *Tak1 *(TGF-β activated kinase 1), *Mekk1 *(MAP kinase kinase kinase), *hep*, *aop *(anterior open), dominant negative form of *bsk *(*Drosophila *Jnk), and *mkp *(MAP kinase phosphatase). The progeny of the co-heterozygotic lines of the MAPK and JNK pathway genes with *Dcp-1 *GF showed extensive, although not complete, lethality. In rare cases, a few progeny reached adulthood although they displayed severe rough eye phenotypes and shortened life span (Additional file 1D). Our finding that expression of *Tak1*, *Mekk1*, *hep*, or *aop *enhanced the rough eye phenotype is consistent with the notion that the JNK pathway positively regulates apoptosis. Paradoxically, we found that expression of the *mkp*, a negative regulator of JNK, or the dominant negative form of *bsk *(*jnk*) enhanced apoptosis in the eye, as well. These data suggest that the interaction between Dcp-1-induced apoptosis and the JNK pathway is not simple but complicated (see Discussion).

### The ecdysone pathway

We identified the EP lines of four ecdysone-regulated genes,*Eip74EF *(ecdysone induced protein 74EF), *Eip78C *(ecdysone induced protein 78C), *broad *(br), or *Eip55E *(ecdysone induced protein 55E) from our screen. Ecdysone is a hormone that regulates the metamorphosis of insects and the ecdysone hormonal signaling is known to induce apoptotic and autophagic cell death during metamorphosis [47]. The co-heterozygote of *Eip74EF*^*EP*(*G*15347) ^in the *Dcp-1 *background enhanced the *Dcp-1 *GF eye phenotype (Figure 4M and 4N). The *Eip78C*^*EP*(*G*14526) ^progeny showed a phenotype similar to *Eip74EF *when they were crossed with *Dcp-1 *GF (Additional file 1E). In contrast, when crossed to one of the *Eip55E *alleles, *Eip55E*^*EP*(*G*13564)^, *Dcp-1 *GF phenotype was suppressed (Figure 3I). The co-heterozygote of one of the *br *alleles, *br*^*EP*(*G*10174)^, with *Dcp-1 *GF rescued the eye phenotype almost completely (Figure 3J), while another allele, *br*^*EP*(*G*1972)^, showed an enhanced phenotype (Data not shown). We noticed that *Eip55E*^*EP*(*G*2166)^, and *br*^*EP*(*G*1972) ^enhanced the rough eye phenotype of *Dcp-1 *GF whereas *Eip55E*^*EP*(*G*13564) ^and *br*^*EP*(*G*10174) ^significantly suppressed the rough eye phenotype. One possibility is that the insertion positions of EP lines may have caused opposite phenotypes. Consistent with this idea, we found that *Eip55E*^*EP*(*G*13564) ^and *br*^*EP*(*G*10174) ^have insertions in the exon region of *Eip55E *and the first intron of *br*, respectively (data not shown) suggesting that these EP elements may reduce the function of the corresponding genes and suppress the cell death phenotype.

### Relationship between autophagy and *Dcp-1*-induced apoptosis

Our initial characterization of the *Dcp-1*-modifier screen suggested that autophagy specifically interacts with *Dcp-1*-induced apoptosis. First, we identified many genes directly involved in autophagy. Second, we identified several components in the insulin/IGF-1/TOR pathway, which has been shown to regulate autophagy. Third, the involvement of ecdysone signaling in autophagic cell death is well documented and we identified several ecdysone-induced proteins in our screen. These findings led us to contemplate the relationship between autophagy and *Dcp-1*.

**Figure 5:**
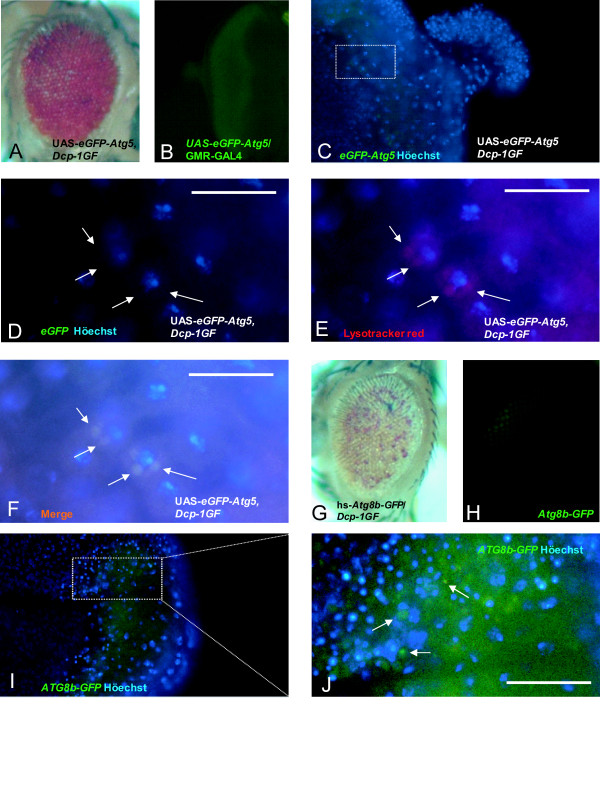
**eGFP-Atg5 and Atg8b-GFP were expressed near condensed chromatin in *Dcp-1 *GF imaginal discs**. (A) Adult eye phenotype of *Dcp-1 *GF;UAS-*eGFP-Atg5*. (B) GFP expression pattern in eye imaginal disc of a GMR-*GAL4*;UAS-*eGFP-Atg5*. (C) Imaginal discs of late third instar larva of *Dcp-1 *GF;UAS-*eGFP-Atg5*. (D) eGFP-Atg5 spots were observed near the morphogenetic furrow. A punctate nucleus was observed adjacent to the GFP spots. (E) LysoTracker Red spots. (F) LysoTracker Red spots and eGFP-Atg5 spots overlapped (white arrows indicate autophagosome). (D, E and F) higher-magnification images of (C). (G) Adult eye phenotype of *Hs-Atg8b-GFP*; *Dcp-1 *GF. (H) GFP expression pattern in eye imaginal disc of *Hs-Atg8b-GFP *larva after heat shock. (I and J) Eye imaginal discs of third instar larva of *Hs-Atg8b-GFP*; *Dcp-1 *GF. (J) Condensed chromatin was detected in the inner part (near the morphogenetic furrow) of the eye imaginal discs. Atg8b-GFP spots were observed near condensed chromatin (12 hour after five 1 hour heat shocks).

To verify our finding that autophagy suppresses *Dcp-1*, we over-expressed eGFP fused to Atg5 (eGFP-Atg5) and the *Dcp-1 *in the eye imaginal disc using the UAS/GAL4 system. Indeed, we found that the eye phenotype was suppressed by eGFP-Atg5 over-expression (Figure 5A). Intriguingly, we noticed that the eGFP-Atg5 level was increased in the *Dcp-1 *GF animals (Figure 5C and 5D) compared with control animals (Figure 5B), suggesting that Dcp-1 over-expression may induce autophagic genes and autophagy. We tested this possibility using LysoTracker Red staining, which is an autophagy marker. The level of LysoTracker Red was higher in the UAS-*eGFP*-*Atg5*/*Dcp-1 *GF animals (Figure 5E and 5F) than in the UAS-*eGFP*-*Atg5 *control animals (Additional file 5E and F). Together, these data suggest that there exists an interaction between autophagy and Dcp-1-induced apoptosis: autophagy suppresses Dcp-1-induced cell death, whereas Dcp-1 induces autophagy. To further demonstrate the Dcp-1-regulated autophagic gene induction, we used UAS-*Atg8b*-*GFP *as an autophagy marker in the *Dcp-1 *GF animals. Similar to Atg5, Atg8b-GFP was induced in these animals (Figure 5J), even though Atg8b-GFP over-expression did not suppress the rough eye phenotype (Figure 5G). These data suggest that the Dcp-1 may be able to increase autophagic gene expression even when autophagy does not suppress Dcp-1-induced apoptosis.

**Figure 6:**
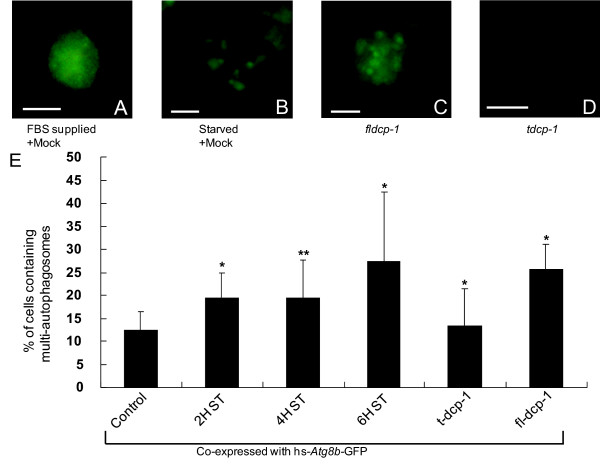
**Expression of *Atg8b-GFP *with *Dcp-1 *in S2 cells**. S2 cells were transfected with *Atg8b-GFP *with or without *Dcp-1 *constructs were maintained under (A) FBS supplied or (B) starvation-inducing culture conditions. (C) Atg8b-GFP was induced with full-length (fl-) Dcp-1 in the presence of FBS. (D) Atg8b-GFP was induced with truncated (t-) Dcp-1. (E) The percentage of cells with multi-autophagosomes was counted by cell number of punctate Atg8b-GFP expression. (n of; control: 1,705, 2 hour starvation: 1,353, 4 hour starvation: 1,464, 6 hour starvation: 1,304, *t-Dcp-1*: 1,019, *fl-Dcp-1*:1,031, (*, P < 0.05; **, P < 0.01 *t *test). Starvation is abbreviated to ST. Scale bars in A-D stand for 10 um. We also tested the expression level of *Dcp-1 *and *Atg8b *in the lines that over-express *Dcp-1 *with *Atg1*, *Atg6*, *Aut1 *and *S*6*k*^*wt *^but we found that the gene expression levels were not significantly affected in any case (Additional file 7).

Next, we confirmed these results using the *Drosophila *S2 cell culture system. We transfected S2 cells with the *Atg8b*-*GFP *construct with or without full-length *Dcp-1 *and monitored the pattern of GFP fluorescence to observe the induction of autophagy. As a positive control, we starved S2 cells and found that starvation, which is a known cause of autophagy, induced Atg8b-GFP punctate expression (Figure 6B). In cells co-transfected with Atg8b-GFP and full-length *Dcp-1*, punctate GFP expression appeared even under replete conditions (Figure 6C), suggesting that Dcp-1 increased autophagy in S2 cells. Taken together, our data imply that increased autophagy suppresses Dcp-1-induced apoptosis and that Dcp-1 in turn positively regulates autophagy through a feedback regulation.

## Discussion

In this study, we identified potentially important genes that interact with fly caspase Dcp-1 through a large-scale *in vivo *screen. We used a GAL4-induced *Dcp-1*-over-expressing strain to screen for modifiers of the caspase pathway in *Drosophila*. Recently, various caspase substrates or effectors were identified through a genome-wide microarray analysis using *Diap1 *knockdown, a caspase inhibitor, and anti-caspase *RNAi *in *Drosophila *embryo-derived Kc cells [48]. To the best of our knowledge, the present study represents the first large-scale *in vivo *screen with a *Drosophila *caspase. Moreover, we used a full-length form of Dcp-1, which has the advantage of mimicking the pre-apoptotic state. As expected, we identified both enhancer and suppressor genes that exacerbated and ameliorated apoptosis, respectively. Moreover, our genetic screen revealed that the phenotype caused by caspase Dcp-1 is suppressed by autophagy gene expression and influenced by genes from other pathways. We showed that *Dcp-1 *GF was rescued by the expression of autophagy genes, *S6k*, ecdysone-inducible genes, such as *Eip55e *and *broad*, and hypomorphic allele of *Tor*. Our data indicate that Dcp-1 activity is negatively controlled by autophagy.

### Relationship between *Dcp-1 *and autophagy

While we found that over-expression of autophagy genes suppressed Dcp-1-induced apoptosis, we also observed that expression of full-length *Dcp-1 *induced autophagy in eye imaginal disc and S2 cell. This autophagy induction caused by full-length *Dcp-1 *expression was very similar to the one induced by starvation in S2 cell. It has been shown that RNAi knock down of *Dcp-1 *reduces autophagy in tumorous larval hemocytes-derived *l(2)mbn *cells during starvation, indicating that Dcp-1 is required for autophagy regulation [49]. In addition, the *Dcp-1*^*prev *^reduction-of-function mutation has been shown to reduce autophagy in region two germaria and stage 8 degenerating egg chambers during *Drosophila *oogenesis [49]. Autophagic cell death has also been shown to be associated with the transcriptional up-regulation of pro-apoptotic genes, including *Dcp-1*, in salivary glands and the mid-gut [47,50,51]. These results are consistent with our observations that autophagy signals were found in the *Dcp-1*-expressing eye discs and in the full-length *Dcp-1 *transfected S2 cells.

Our findings showed that the *Dcp-1 *GF phenotype was rescued by autophagy and that autophagy was in turn induced by *Dcp-1 *expression. This seemingly paradoxical relationship between apoptosis and autophagy is not unprecedented. It has been shown that the autophagy gene, *Atg5*, also plays important roles in the interplay between autophagy and apoptosis. The full length Atg5 protein is an essential component for autophagy induction. However, when Atg5 is cleaved by calpain, it loses the ability to induce autophagy. Instead, the truncated Atg5 interacts with BCL-XL, is translocated to mitochondria, and causes apoptosis [52,53]. Interestingly, we found that full-length Dcp-1 could also induce autophagy, whereas the truncated active Dcp-1 resulted in apoptosis. Thus, our results suggest that Dcp-1 may act as a key protein in the regulation of not only apoptosis but also autophagy, similar to Atg5.

It remains to be determined how full-length *Dcp-1 *induces autophagy. It is tempting to speculate that full-length *Dcp-1*, which is a less active form, causes autophagy as a defensive mechanism at a very early stage in response to cellular damages. When the cellular damages reach the threshold level for apoptosis, Dcp-1 is cleaved to its active form and facilitates full-fledged apoptosis. Consistent with this idea, previous studies have shown that autophagy delays apoptosis, perhaps by eliminating unwanted or damaged molecules, to establish cellular homeostasis at the initial stages of cisplatin injury [54]. Further studies are needed to test the interaction between Dcp-1 and autophagy proteins and to elucidate the molecular mechanisms linking Dcp-1 and autophagy.

### Other signaling pathways that interact with *Dcp-1*

Our genetic screening of EP lines and their interactions with other candidate lines allowed us to identify genetic pathways other than the classical apoptotic pathway. The major signal that triggers entry into metamorphosis and the activation of autophagic cell death in *Drosophila *is a high-titer pulse of ecdysone (20-hydroxyecdysone) that occurs during puparium formation [55-57]. The ecdysone receptor complex and the nuclear receptor competence factor ftz-f1 lead to salivary gland death through the transcriptional activation of a set of "early" gene transcription factors, *br*, *Eip74EF*, and *Eip93F*, which in turn regulate the expression of "late" effector genes that appear to function more directly in apoptosis [56]. Mutations in each of *ftz-f1*, *br*, *Eip74EF*, and *Eip93F *impair salivary gland degeneration during metamorphosis at different stages in the cell death process. *Eip93F *seems important in autophagic induction because mutations in this gene produce an early block in the formation of autophagic vacuoles in the salivary gland and midgut cells [47,58]. In conclusion, apoptotic and autophagic cell deaths during *Drosophila *metamorphosis seem to be either combined or sequential, depending on various factors. Serial analysis of gene expression (SAGE) and microarray analyses have revealed that *Eip93F *is required for the transcriptional up-regulation of genes that are activated in dying salivary glands, including genes involved in autophagy, apoptosis, non-caspase proteolysis, and cytoskeletal remodelling [50].

A GMR-*Hid *modifier screen and *Hs-Hid *suppression assay showed that Hid is inactivated by the ERK/MAPK pathway [59]. Even though our data differ from previous findings that *Dcp-1 *is down-regulated by *reaper *and *grim *but not by *Hid *[22], our screen data uncover a relationship between *Dcp-1 *and *Hid*, as well as between *Dcp-1 *and *MAPK*. Co-expression of GMR-*Hid *and GMR-full-length *Dcp-1 *line leads to a slight increase in eye roughness and pigment loss [22]. A similar phenomenon was observed in the present study with our *Dcp-1 *GF/*CyO *line (Additional file 6). These results suggest that *Hid *affects *Dcp-1*, albeit not as strongly as *grim *and *reaper*. The pro-apoptotic protein Hid may also have a role in autophagic induction [60]. Dcp-1 activity was shown to be regulated by Diap1 and Hid [61]. These reports suggest that autophagic cell death in organs, which undergo metamorphosis, is regulated by ecdysone, Eip93F, and pro-apoptotic proteins. Thus, we propose that Dcp-1 and Hid regulate autophagy and that Dcp-1 may regulate autophagy by receiving a signal from Hid through a non-apoptotic pathway.

### Opposite phenotypes and allelic differences

Some of the transgenic lines that we tested in this study showed somewhat unexpected phenotypes. For example, while the enhancement of the rough eye phenotype by co-expression of Tak1, Mekk1, hep, or aop is consistent with the notion that the JNK pathway positively regulates apoptosis, the expression of *mkp*, a negative regulator of JNK, or the dominant negative form of *bsk *(*jnk*) also enhanced the eye phenotype. Likewise, we found that various reduction-of-function mutations of *Tor *either enhanced or suppressed the rough eye phenotype (Figure 3G and Additional file 1). Both the JNK pathway and the TOR pathway have been shown to affect apoptosis and autophagy [62-65]. Therefore, we speculate that the different expression levels of these signaling pathway genes may lead to survival or death of the cells by differentially affecting autophagy and apoptosis. For example, a mild increase in autophagy may lead to cell survival by suppressing the apoptosis caused by Dcp-1 over-expression, whereas strong induction of autophagy may worsen the cell death phenotype through the combination of apoptotic and autophagic cell deaths [56,57,66,67]. Consistent with this idea, whereas Atg1 is essential for autophagy, strong induction of Atg1 has been shown to result in cell death [68]. Thus, it is possible that different levels of autophagic induction in the flies mentioned above may explain the seemingly unexpected phenotypes.

Also, we noticed that the *Bchs *alleles (EP(2)2299, G12113, and G13044) enhanced the *Dcp-1 *GF severely. However, the *Bchs*^*EP*(*G*2362) ^line can suppress the rough eye phenotype (Additional file 6). Therefore, we further examined the insertion positions of these EP lines. *Bchs*^*EP*(*G*2362)^, *Bchs*^EP(G12113)^, *Bchs*^EP(G13044) ^were inserted at 1182, 985, and 727 base pairs upstream from the start codon, respectively (data not shown). We speculate that perhaps the insertion positions determined the expression level of *Bchs *and affected the rough eye phenotype differently.

## Conclusions

In this study, we aimed to identify genes that modify Dcp-1 function in a large-scale genetic screen. We demonstrate that the effector caspase Dcp-1 regulates and/or is regulated by autophagy, ecdysone signals, ubiquitination signals, JNK, MAPK, and various transcription factors and cell death signals. Our genetic screen provides a wealth of information on various genes and pathways that may regulate caspase functions for future research. Furthermore, our findings on the integrated regulation between autophagy and *Dcp-1 *could aid the elucidation of molecular mechanisms connecting autophagy and apoptosis.

## Methods

### Fly stocks

*w*^1118^, BL11218 *Tor*^*k*17004^, BL5368 UAS-*Egfr*, BL6292 UAS-*Rac1N17*, BL5844 UAS-*Hsc70-4.D206S*, and BL5613 UAS-*Dl*^*DN *^were obtained from the Indiana University Bloomington Stock Center. For screening, ~1,500 EP lines were obtained from the Szeged *Drosophila *Stock Center, and ~13,000 EP lines were obtained from GenExel Inc. Eye-specific GMR-*GAL4*, wing-specific MS^1096^-*GAL4*, *AP-GAL4*, and other signal transduction- related lines used for screening were kind gifts from Dr. J. Chung (KAIST, South Korea). To make transgenic flies carrying *Dcp-1*, pUAST-*Dcp-1 *was injected into *Drosophila *embryos (*w*^1118^) prior to the time of pole cell formation using a microinjector model IM300 (Narishige, Japan) and an Axiovert25 micromanipulator (Carl Zeiss, Germany). UAS-*Dredd*, UAS-*Dark*, and UAS-*Dronc *lines were generated using the same method that was used for creating the UAS-*Dcp-1 *line. Fly cultures and crosses were maintained at 23.5°C.

### Generation of caspase constructs

To make template cDNA, total RNA from all developmental stages (embryo to adult) was extracted using Trizol reagent (MRC, Inc). Reverse transcription was carried out using Sensiscript RT kit (Qiagen) and Oligo (dT) 12-18 primer (Invitrogen) following manufacturer's instruction. To generate the UAS-*Dcp-1 *vector, an upstream primer with a *Bgl*II site and a downstream primer with an *Xba*I site were designed as follows: forward (5'-AAC AGA TCT ATG ACC GAC GAG TGC GTA AC-3'), reverse (5'-AGT TCT AGA CTA GCC AGC CTT ATT GCC GT-3'). To generate UAS-*Dredd*, UAS-*Dark*, andUAS-*Dronc*, primers were designed as follows: UAS-*Dredd*, forward (5'-CTCGA ATTCA TGGCC GGATC AAACC TGTT-3') and reverse (5'-GCGCT CGAGT CACAG ACGAG GTGGA AAG-3'); UAS-*dark*, forward (5'-ATAGC GGCCG CATGG ATTTT GAAAC TG-3') and reverse (5'-GCTCT CGAGT CATGA ACTGG CCTCC TCC-3'); UAS-*Dronc*, forward (5'-AAAAG ATCTA TGCAG CCGCC GGAGC T-3') and reverse (5'-GCGTC TAGAC TATTC GTTGA AAAAC CCGGG A-3'). Cloning was performed using standard methods.

### Electron microscopy of fly eyes

The surfaces of eyes from wild-type *Drosophila *or from flies with single or double copies of *Dcp-1 *GF were examined using a Scanning Electron Microscope (SEM; Leo 1455VP, Leo Electron Microscopy. Ltd., Korea Basic Science Institute).

### Genetic screening with UAS-*Dcp-1 *and EP lines

We first mapped several UAS-*Dcp-1 *lines and selected the line UAS-*Dcp-1*^19-2^, which has an insertion in chromosome 2R at position 12987659 between *GstS1 *and CG30456. Male flies from this line were crossed with GMR-*GAL4 *virgin female flies. Progeny virgin flies showing the unique *Dcp-1 *GF phenotype were crossed with *Bc*/*CyO *males. Among those offspring, flies having the same phenotype as their mother as well as curled wings were isolated. These flies possess GMR-*GAL4 *and UAS-*Dcp-1 *on the second chromosome as a result of homologous recombination. To conduct the screen, we performed the crossing of *Dcp-1 *GF/*CyO *virgin females with males from the EP lines. Flies showing any phenotype that differed from that of the *Dcp-1 *GF flies were selected. The eye phenotypes from the selected flies were photographed using a Carl Zeiss Stemi 2000C microscope with Axio Vision AC software. Inverse polymerase chain reaction (PCR) and the sequencing of the selected EP lines were both performed according to the descriptions found at http://www.fruitfly.org/about/methods/inverse.pcr.html.

### Induction of Hs-*Atg8b-GFP *flies

*yw*, *Hs-Atg8b-GFP*/*yw*, *Hs-Atg8b-GFP *(*Hs-Atg8b-GFP*) flies were crossed with *Dcp-1 *GF/*CyO*. Then, *yw*, *Hs-Atg8b-GFP*/+; *Dcp-1 *GF/+ and *yw*, *Hs-Atg8b-GFP*/+; *CyO*/+ progenies were collected and crossed to each other. *yw*, *Hs-Atg8b-GFP*/*yw*, *Hs-Atg8b-GFP*; *Dcp-1 *GF/*CyO *(*Hs-Atg8b-GFP*; *Dcp-1 *GF) flies were selected by GFP positives with *Dcp-1 *GF adult eye phenotype. *Hs-Atg8b-GFP*; *Dcp-1 *GF and *Hs-Atg8b-GFP *control flies were allowed to lay eggs, which developed to the early third instar larval stage. Then, these larvae were heat shocked five times at 37°C for 1 hour with a 30-min break interval. About 6 hour after the last heat shock, when the flies had reached the climbing late third instar larval stage, larvae were collected and dissected.

### UAS-eGFP-*Atg5 *flies

*wg/CyO*; UAS-*eGFP-Atg5 *flies were crossed with *Dcp-1 *GF/*CyO *or GMR-*GAL4 *flies. Then, *Dcp-1 *GF;UAS-*eGFP-Atg5 *and GMR-*GAL4; *UAS-*eGFP-Atg5 *flies were collected and dissected separately.

### Eye imaginal disc preparation and staining

Eye imaginal discs were dissected from third instar larvae in PBS. Discs were incubated for 1 min in 100 μM LysoTracker Red (Molecular Probes) and 1 μM Höechst 33342 in PBS. Collected discs were washed twice with PBS, mounted on slide glasses with 80% glycerol in PBS on glass slides with cover slips, and immediately photographed using a Leica DM6000B microscope.

### S2 cell culture and transfections

S2 cell culture and transfections were performed as previously reported [69]. Full-length and truncated *Dcp-1 *coding regions were subcloned into the pENTR directional TOPO vector (Invitrogen) and then cloned into the *Drosophila *gateway vector pHWF by the LR recombination reaction with the LR clonase enzyme mix (Invitrogen) following the provided protocol. The UAS-*Dcp-1 *construct was used for the PCR template. To make *Atg8b-GFP *construct, the *Atg8b *coding region was subcloned into the pENTR directional TOPO vector (Invitrogen) and the relevant construct region was cloned into the *Drosophila *gateway vector pHGW by performing the LR recombination. The pHS-*GA8 *construct from the Neufeld laboratory was used as a PCR template. Full-length *Dcp-1 *primers were designed as follows: forward (5'-CACCATG ACC GAC GAG TGC GTA ACC AGA-3'), reverse (5'-GCC AGC CTT ATT GCC GTT CGG CTT GT-3'). Truncated *Dcp-1 *primers were designed as follows: forward (5'-CACCATG GCC AAG GGC TGT ACG CCG GAG-3'), reverse (5'-GCC AGC CTT ATT GCC GTT CGG CTT GT-3'). The full-length and truncated primer sequences were identical to those used previously [22], except for the CACCATG in the upstream primer for TOPO cloning. *Atg8b-GFP *primers were designed as follows: forward (5'-CACCATG GAT ATG AAC TAC CAG TA-3'), reverse (5'-CTA CTG CCG TCC ATA GAC GT-3'). Cells were plated on round cover glasses inserted into 6-well plates (NUNC).

### Starvation induction

48 hours after transfection of S2 cells, the culture medium was replaced with complete Schneider's medium (GIBCO) containing FBS. After 12 hours stabilization, starvation was induced by changing the complete medium to Hank's balanced salt solution with CaCl_2 _and MgCl_2 _and without phenol red (HBSS; Welgene Inc.). After 3 hours, the HBSS medium was replaced with Schneider's medium without FBS.

### Observation and quantification of GFP fluorescence

The transfected cells attached on the cover glasses were fixed with 2% final concentration of paraformaldehyde solution for 10 min and washed three times with PBS and mounted on glass slides. The cells on the cover glasses were mounted on the slide glasses with nail polish and photographed using a Leica DM6000B microscope. The GFP-positive spots in the cells were counted from top to bottom and again from left to right under the 1000× magnification. Transfected cell populations were then counted three times with three different batches of transfection and the results were normalized. The tests were done blindly and the statistical analysis was performed by using Student's *t*-test.

### Quantitative RT-PCR and real-time PCR

Fly lines containing UAS-*Dcp-1 *alleles were crossed to GMR-*GAL4 *flies, and their progeny were grown to adulthood. Heads from virgin progeny were collected. For each sample, the same number of collected heads was ground in Trizol reagent (Invitrogen) using a hand pestle. Total RNA was purified using the provided protocol. Reverse transcription was carried out with the Sensiscript RT kit (Qiagen) and Oligo (dT)12-18 primer (Invitrogen) using the provided protocols [70]. The resulting cDNA was used for PCR. QRT-*Dcp-1 *primers were designed as follows: forward (5'-TCG ACG AGC TAC AAG ATA-3'), reverse (5'-GCT GGT TAA CGA ATG TAA-3'). PCR was performed as follows: 5-min denaturation at 95°C followed by 25 cycles with 94°C for 30 sec, 52°C for 30 sec, and 72°C for 30 sec. *w*^1118 ^genomic DNA and cDNA from *w*^1118 ^heads were used as control templates. Real-time PCR was performed using the IQ5 real-time PCR detection system (Bio-Rad). Amplification was performed using IQ5 SYBR green supermix (Bio-Rad). Template cDNAs were prepared as previously described [70]. At least three independent experiments were performed for each sample. PCR conditions and primers used in each reactions were designed as follows: 3-min denaturation at 95°C followed by 40 cycles with 95°C for 30 sec, 52°C for 30 sec. Elongation was then performed at 72°C for 30 sec followed by 72°C for 1-min and followed by 81 cycles of Melt curve analysis from 55°C to 95°C. QRT-*Dcp-1 *primers were designed as previously described. QRT-*Atg8b *primers were designed as follows: forward (5'-AGT TCT ACT TTC TCA TCC GC-3'), reverse (5'-CAT AGA CGT TCT CAT CGG TAT-3'). QRT-*Tor *primers were designed as follows: forward (5'-ACC ACA AAC GAA CTA CGA-3'), reverse (5'-TAC CTT GTG AGC AGA CCT-3'). QRT-*RP49 *primers were designed as follows: forward (5'-AGA TCG TGA AGA AGC GCA CC-3'), reverse (5'-CGA TCC GTA ACC GAT GTT GG-3'). Real-time PCR results were analyzed using IQ5-2.0 software (Bio-Rad). Each of individual expression level was normalized with *w*^1118 ^expression level of reaction sets of same primers and with *RP49 *expression level of reaction sets of same templates.

### Caspase activity test

Caspase activity was determined using the Caspase-Glo^® ^3/7 Assay (Promega) and measured using a Wallac Vitor 1420 multilabel counter (Perkin-Elmer). Each lysate was prepared by grinding ~20 heads of virgin progeny of *w*^1118^, *Dcp-1 *GF and crossed progeny of *Dcp-1 *GF with various autophagy-related lines. The heads were homogenized and sonicated in lysate buffer supplemented with 1 mM phenylmethylsulfonyl fluoride (*PMSF: *Serine specific protease inhibitor). The lysates were centrifuged to remove debris-like cuticles and cell membranes. The lysates were immediately mixed with Caspase Glo3/7 reagent as 1:1 ratio individually. Samples were incubated for 30 min at room temperature. Luminescence of each sample was then measured by using Wallac Vitor 1420 multilabel counter (Perkin-Elmer). At least three independent experiments were performed for each genotype. Statistical analysis was performed by using Student's *t*-test.

## Authors' contributions

YIK managed overall experiments and performed S2 cell experiments, molecular biology experiments and *Drosophila *experiments, and wrote the manuscript. TY performed primary screen with random selected EP lines. YIK and JL confirmed the selected lines from the screening. YSH carried out the RNA experiments including the quantitative RT-PCR. JA participated in the design of the study and helped writing the manuscript. SJL participated in experimental designs and coordination, and helped writing the manuscript during the revision. OJY conceived the study, and participated in experimental designs and coordination and helped writing the manuscript.

All authors read and approved the final manuscript.

## Supplementary Material

Additional file 1 **Table S1**. Enhancers and suppressors of the eye phenotype caused by *Dcp-1 *over-expression. Click here for file

Additional file 2 ***GAL4 *mRNA expression level was constant in every GAL4 mediated line**. (A) The level of *GAL4 *expression was not detected in the *w*^1118^. The level of *GAL4 *expression was constant in every GAL4 mediated line. (B) The expression level of *GAL4 *was compared with that of *RP49 *by using the real-time PCR experiment. (NT: No template/GG: GMR-*GAL4*, homozygote/GD: *Dcp-1 *GF/*CyO*/A1D: *Atg1*^*EP*(*G*13748)^/*Dcp-1 *GF/A6D: *Atg6*^*EP*(*G*6854)^/*Dcp-1 *GF) Click here for file

Additional file 3 **Expression level of *CG10969*, down-stream gene of *Atg1*, was not affected by EP inserted up-stream of *Atg1***. (A) The surrounding genes of *Atg1*. CG10969, CG17666, CG17667 and CG11006 are located next to the *Atg1*. However, the directions of CG17666, CG17667 and CG11006 are opposite to the direction of EP (G13748) insertion. CG10969 is placed in down-stream site of *Atg1 *and EP (G13748). (Black arrow indicates the P-element insertion site of *Atg1*^*EP*(*G*13748).^). (B) The genomic DNA of *w*^1118 ^was used as a template for the lane 1, while appropriate cDNAs were used for the lanes 2, 3, 4 and 5. The lane 1 indicates that all the primer sets worked properly. The lane 2 indicates that *Atg1 *and CG10969 were not expressed in the *w*^1118^. The lane 3 indicates that *Atg1 *was slightly up-regulated by the expression of *Dcp-1*. The lane 4 and 5 indicates that *Atg1 *expression level was increased in GMR-*GAL4*/*Atg1*^*EP*(*G*13748) ^and GMR-*GAL4*, *Dcp-1*/*Atg1*^*EP*(*G*13748)^. No expression was detected by the CG10969 primer set. Click here for file

Additional file 4 **Expression level of *Tango5*, down-stream gene of *Atg8a*, was not affected by EP inserted up-stream of *Atg8a***. (A) The surrounding genes of *Atg8a*. CG1826 and CG15221 are located up-stream site of *Atg8a*. *Tango5 *is located down-stream of *Atg8a *and EP (G9749). (Black arrow indicates P-element insertion site of *Atg8a*^*EP*(*G*9749)^*)*. (B) Genomic DNA of *w*^1118 ^was used as a template for the lane 1, while appropriate cDNAs were used for the lanes 2, 3, 4 and 5. The lane 1 indicates that all the primer sets worked properly. The lane 2 indicates that *Atg8a *and *Tango5 *were expressed in the *w*^1118 ^as the baseline level. The lane 3 indicates that *Atg8a *was up-regulated by the expression of *Dcp-1*. As showed in the lane 4 and 5,*Atg8a *expression level was increased in GMR-*GAL4*/*Atg8a*^*EP*(*G*9749) ^and GMR-*GAL4*, *Dcp-1*/*Atg8a*^*EP*(*G*9749)^. The level of *Tango5 *expression was detected slightly in the Lane 2, 3 and 4. However, *Tango5 *expression level was not increased by *Atg8a*^*EP*(*G*9749)^. Click here for file

Additional file 5 **eGFP-Atg5 and Atg8b-GFP were expressed by GMR-*GAL4 *in imaginal discs**. (A) Merged image of GFP expression pattern and Höechst 33342 stained pattern in eye imaginal disc of a GMR-*GAL4*;UAS-*eGFP-Atg5*. (B) eGFP-Atg5 expression pattern in eye imaginal disc of a GMR-*GAL4*;UAS-*eGFP-Atg5*. (B, C and D) Higher-magnification images of (A). (C) LysoTracker Red spots. (D) LysoTracker Red spots and eGFP-Atg5 spots were overlapped (white arrows indicate autophagosome). (E and F) Merged image of GFP expression pattern and Höechst 33342 stained pattern in eye imaginal disc of *Hs-Atg8b-GFP *larva after heat shock. (12 hour after five 1-hour heat shocks). Click here for file

Additional file 6 **List of positive candidates from the screen**. This file was originally made by using the MS Access and transformed to the PDF format provided here. Click here for file

Additional file 7 **Expression level test by real time PCR**. (A) *Dcp-1 *expression level was increased in *Dcp-1 *GF flies that crossed with autophagy-related EP strains. (B) *Atg8b *expression level was increased in autophagy strains that crossed with GMR-*GAL4 *or *Dcp-1 *GF. (C) *Tor *expression level was reduced ~60% in, *Tor*^*k*17004^/*Dcp-1 *GF and *Tor*^*k*17004^/GMR-*GAL4, Tor *mutants. However, the expression level was not changed in *Tor*^DN^/*Dcp-1 *GF line. (D) *Atg8b *expression level was increased in each of the *Tor *mutants. *Atg8b *expression level was slightly increased in the *Tor *mutants crossed with *Dcp-1 *GF than that of the flies crossed with GMR-*GAL4*. At least three individual experiments for each sample were put together for the gene expression study function in IQ5 2.0 and the expression levels were normalized to the reference gene RP-49. The control sample was obtained from *w*^1118 ^wild-type flies. The error bars represent the SD of ddCt value. GD indicates GMR-*GAL4*;UAS-*Dcp-1 *and GG indicates GMR-*GAL4*. Click here for file
