# Supplementary material for: A genetic screen for modifiers of Drosophila caspase Dcp-1 reveals caspase involvement in autophagy and novel caspase-related genes
Source: BMC Cell Biol. 2010 Jan 25;11:9. doi: 10.1186/1471-2121-11-9 (PMC2822743; doi:10.1186/1471-2121-11-9)
Supplement: Additional file 1 — Table S1. Enhancers and suppressors of the eye phenotype caused by Dcp-1 over-expression. [file 1471-2121-11-9-S1.DOC]

## Table S1-Enhancers and suppressors of the *Dcp-1* over-expression eye phenotype

| **Gene** | **Fly Base ID** | | | **Line number** | **Modification** | | | | | | **Homologs and functions** |
| --- | --- | --- | --- | --- | --- | --- | --- | --- | --- | --- | --- |
| **A. Apoptotic signaling** | | | | | | | | | | | |
| *th* | | CG12284 | EP(3)3279^*^ | | P: Ssu | | | O:Ssu | | | XIAP, ubiquitin-protein ligase activity |
| *p35* | |  | UAS-*p35* | | P: Ssu | | | O:Ssu | | | Pan-caspase inhibitor, Baculovirus protein |
| *faf* | | CG1945 | EP(3)3520^*^ | | Sen | | | | | | ubiquitin-specific protease activity |
| *eff* | | CG7425 | G15069^*^ | | Sen | | | | | | ubiquitin-conjugating enzyme E2D 3 isoform 2, ubiquitin-protein ligase activity |
| **B. Autophagy signaling** | | | | | | | | | | | |
| *Aut1* | | CG6877 | G3894^*^ | | P: Ssu | | | O:Ssu | | | E2-like enzyme conjugated PE to Atg8 |
| *bchs* | | CG14001 | G2362^+^ | | P: Su | | | O: Psu# | | | *Alfy* (autophagy linked FYVE)  Lysosomal transport, autophagy-linked |
|  |  |  | EP(2)2299^*^  G12113^*^, G13044^+^ | | Sen | | | | | |  |
| *SNF4Aγ* | | CG17299 | GX6409^*^ | | P: Su | | | O:Su | | | AMPK *γ* subunit , serine/threonine kinase activity |
| *Atg1* | | CG10967 | G13748^+^ | | P: Su | | | O:Su | | | protein serine/threonine kinase activity |
| *Atg2* | | CG1241 | G6691^+^ | | P: Su | | | O:Su | | | unknown |
| *Atg4* | | CG4428 | G18114^+^ | | P: Psu# | | | O:Su | | | cysteine-type endopeptidase activity, cleaves at C-term of Atg8 |
| *Atg6(Beclin)* | | CG5429 | G6854^+^ | | P: Su | | | O:Su | | | Beclin-1, protease inhibitor activity,  autophagic cell death |
|  |  |  | G3772^+^ | | P: Psu | | | O:Su | | |  |
| *Atg7* | | CG5489 | G8010^+^ | | P: Psu | | | O:Su | | | transmembrane transporter activity |
| *Atg8a* | | CG32672 | G9749^+^ | | P: Su | | | O:Su | | | LC3, microtubule binding, |
| *Atg18* | | CG7986 | G3744^+^ | | P: Su | | | O:Psu | | | Peripheral membrane protein, binds PtdIns(3)P |
| **C. Insulin /IGF and Tor signaling** | | | | | | | | | | | |
| *Tor* | | CG5092 | BL11218 *Tor^k17004^*^*^ | | P: Psu | O:Su | | | | | Tor, protein kinase activity |
| *Akt1* | | CG4006 | UAS-*Akt*  #3^*^ | | Sen | | | | | | AKT3 (STK-2), protein serine/threonine kinase activity |
| *S6k* | | CG10539 | UAS-*dS6K* | | P: Su | | O:Su | | | | ribosomal protein S6 kinase activity; protein serine/threonine kinase activity |
| *Pten* | | CG5671 | UAS-*Pten*/*CyO* ff20.2^*^ | | Lethal | | | | | | phosphatidylinositol 3-phosphatase activity |
| *Pi3k92E* | | CG4141 | UAS-*Pi3K^wt^** | | Sen | | | | | | phosphatidylinositol 3-kinase activity |
| **D. MAP kinase and Jun N-terminal kinase (JNK) signaling** | | | | | | | | | | | |
| *Mekk1* | | CG7717 | UAS-*DMEKKla3-5^*^* | | Sen | | | | | | MAP kinase kinase kinase activity |
| *mkp* | | CG34099 | UAS-*mkp/CyO* ^*^ | | Sen | | | | | | MAP kinase phosphatase activity |
| *hep* | | CG4353 | UAS-*hepCA* #3^*^ | | Sen | | | | | | JUN kinase activity/MAP kinase activity |
| *Tak1* | | CG18492 | UAS-*dTak1* ^WT4*^ | | Lethal | | | | | | JUN kinase kinase activity |
| *bsk* | | CG5680 | UAS-*DJNK*^DN*^ | | Lethal | | | | | | JNK beta2, JUN /MAP kinase activity |
| *aop* | | CG3166 | EP(2)0598^*^ | | P: Ssu | | O:Ssu# | | | | ETV6, transcription factor activity, downstream of JNK |
| **E. Ecdysone signaling** | | | | | | | | | | | |
| *Eip74EF* | | CG32180 | G15347^*^ | | Sen | | | | | | transcription factor activity |
| *Eip78C* | | CG18023 | G14526^*^ | | Sen | | | | | | transcription factor activity |
| *br* | | CG11491 | G10174^*^ | | P: Ssu | | O:Su | | | | transcription factor activity |
|  |  |  | G1972^*^ | | Sen | | | | | |  |
| *Eip55E* | | CG5345 | G2166^+^ | | En | | | | | | cystathionine gamma-lyase activity |
|  |  |  | G13564^+^ | | P: Ssu | | O:Su | | | |  |
| **F. Ubiquitination and SUMOylation signaling** | | | | | | | | | | | |
| *Ubp64E* | | CG5486 | G5032^+^, G5401^+^ | | P: Ssu | | O:Psu# | | | | ubiquitin-specific protease activity |
| *UbcD4* | | CG8284 | G4874^+^ | | En | | | | | | ubiquitin-protein ligase activity |
| *Uba1* | | CG1782 | G3534^+^ | | En | | | | | | ubiquitin-protein ligase activity |
| *Ubc-E2H* | | CG2257 | G9680^+^ | | En | | | | | | ubiquitin-protein ligase activity |
| *Cul-5* | | CG1401 | EP(3)3390^*^ | | Lethal | | | | | | calcium mobilizing receptor activity |
| *Uba2* | | CG7528 | G6571^+^, G8583^+^ | | En | | | | | | SUMO activating enzyme activity |
|  |  |  | G4384^+^ | | P: Psu | | O:Psu | | | |  |
| **G. Various developmental signaling** | | | | | | | | | | | |
| *EGFR* | | CG10079 | BL5368 UAS-*Egfr^*^* | | Sen | | | | | | epidermal growth factor receptor activity |
| *PVR* | | CG8222 | UAS-*λPVR-1*^*^ | | Sen | | | | | | PDGF, protein-tyrosine kinase activity |
| *Rac1* | | CG2248 | BL6292 UAS-*Rac1*^N17*^ | | Lethal | | | | | | GTPase activity; GTP binding |
| *Delta* | | CG3619 | BL5613 UAS-*Dl*^DN *^ | | Sen | | | | | | Notch binding |
| *wnd* | | CG8789 | G14551^*^ | | Sen | | | | | | ZPK, protein serine/threonine kinase activity |
| *sgg* | | CG2621 | UAS-*sgg(poor)* ^*^ | | Lethal | | | | | | GSK3-β, glycogen synthase kinase 3 activity |
| *Sec61α* | | CG9539 | G3142^*^ | | Sen | | | | | | protein transmembrane transporter activity |
| *jigr1* | | CG17383 | EP(3)3354^*^ | | En | | | | | | MADF subfamily of SANT domain |
| *Hsp70Aa* | | CG31366 | BL5844 UAS-*Hsc70*^-4*^ | | Sen | | | | | | ATP binding |
| *hdc* | | CG15532 | G2198^*^ | | Sen | | | | | | unknown |
| *GST S1* | | CG8938 | EP(2)0670^*^ | | En | | | | | | glutathione transferase activity |
| **H. Transcription factors** | | | | | | | | | | | |
| *psq* | | CG2368 | G7540^*^ | | Sen | | | | | | transcription factor activity |
| *Nej* | | CG15319 | EP(X)1179^*^ | | Lethal | | | | | | CREB binding protein, transcription co-activator activity |
| *HPS* | | CG12855 | G2504^*^ | | P: Psu | O:Su | | | | | protein binding; zinc ion binding |
| *kay* | | CG33956 | G3984^*^ | | Lethal | | | | | | FOS, transcription factor activity |
| *Fmr1* | | CG6203 | EP(3)3517^*^ | | Sen | | | | | | FMR1, mRNA binding; protein binding |
| *Esg* | | CG3758 | EP(2)2009^*^ | | Sen | | | | | | RNA polymerase II transcription factor activity |
|  |  |  | EP(2)0633^*^ | | Sen | | | | | |  |
| *enc* | | CG10847 | G4432^*^ | | En | | | | | | nucleic acid binding |
| *da* | | CG5102 | G3407^*^ | | Sen | | | | | | transcription factor activity |
| CG6854 | | CG6854 | G15984^*^ | | Sen | | | | | | transcription factor activity |
| CG3065 | | CG3065 | EP(2)316^*^ | | P: Su | | | O:Su | | | transcription regulator activity |
| *Bun* | | CG5461 | EP(2)2447^*^ | | Lethal | | | | | | TSC22 domain family, transcription factor activity |
| *Alhambra* | | CG1070 | G14436^*^ | | P: Su | | O:Su | | | | transcription factor activity |
| **I. Translation factors** | | | | | | | | | | | |
| *Atx2* | | CG5166 | EP(3)3145^*^ | | Sen | | | | | | ATAXIN 2, mRNA binding |
| *Ago2* | | CG7439 | G8907^*^ | | P: Ssu | | | | O:Psu | | translation initiation factor activity |
| *Ef1α48D* | | CG8280 | G2194^*^ | | En | | | | | | translation elongation factor activity |
| **J. signaling undefined genes and novel genes** | | | | | | | | | | | |
| CG9813 | | CG9813 | G16835^*^ | | En | | | | | | unknown |
| CG6685 | | CG6685 | G6729^+^ | | P: Su | | | | | O:Su | unknown |
| CG6301 | | CG6301 | EP(2)2402^*^ | | Sen | | | | | | unknown |
| CG4747 | | CG4747 | EP(2)0594^*^ | | Lethal | | | | | | cytokine-like nuclear factor n-pac, |
| CG4860 | | CG4860 | G4748^+^ | | P: Su | | | | | O:Su | acyl-CoA dehydrogenase activity |
|  |  |  | G9085^+^, G14823^+^ | | P: Ssu | | | | | O:Psu |  |
| CG17836 | | CG17836 | G4693^*^ | | Sen | | | | | | mucin5, protein dimerization activity |
| CG12262 | | CG12262 | G5568^+^, G13743^+^ | | P: Psu | | | | | O:Psu | acyl-CoA dehydrogenase activity |
| CG11505 | | CG11505 | G15474^*^ | | Sen | | | | | | LARP4 |
| CG14696 | | CG14696 | G15368^*^ | | Sen | | | | | | unknown |
| CG3902 | | CG3902 | G1851^+^ | | P: Su | | | | | O:Su | unknown |
| CG31217 | | CG31217 | G4511^*^ | | En | | | | | | serine-type endopeptidase activity |
| ^a^Line numbers that start with “G” or “GX” were selected from GenExel *Drosophila* EP lines; that start with “EP” were selected from the Szeged EP lines. ^b^“P” and “O” in modification column mean modification of pigment and order of ommatidium respectively. ^c^En, enhancer; Sen, strong enhancer; Su, suppressor; Ssu, strong suppressor; Psu, partial suppressor. ^d^Homologs and functions of autophagy genes (Table S1A) were noted from Levine and Klionsky [4]. ^e^Homolog names in Tables S1B, C, D and E were selected by NCBI protein blast search. *Selected from primary unbiased large scale screening, +selected from additional secondary ubiquitin and autophagy related lines screening, #lines showing phenotype variations by penetrance. (Some fly lines involved previously described Dcp-1-interacting apoptosis-related genes; these were omitted from the Table S1 but were included in the Additional file 6.) | | | | | | | | | | | |
