# Supplementary figures and images for: A genetic screen for modifiers of Drosophila caspase Dcp-1 reveals caspase involvement in autophagy and novel caspase-related genes
Source: BMC Cell Biol. 2010 Jan 25;11:9. doi: 10.1186/1471-2121-11-9 (PMC2822743; doi:10.1186/1471-2121-11-9)

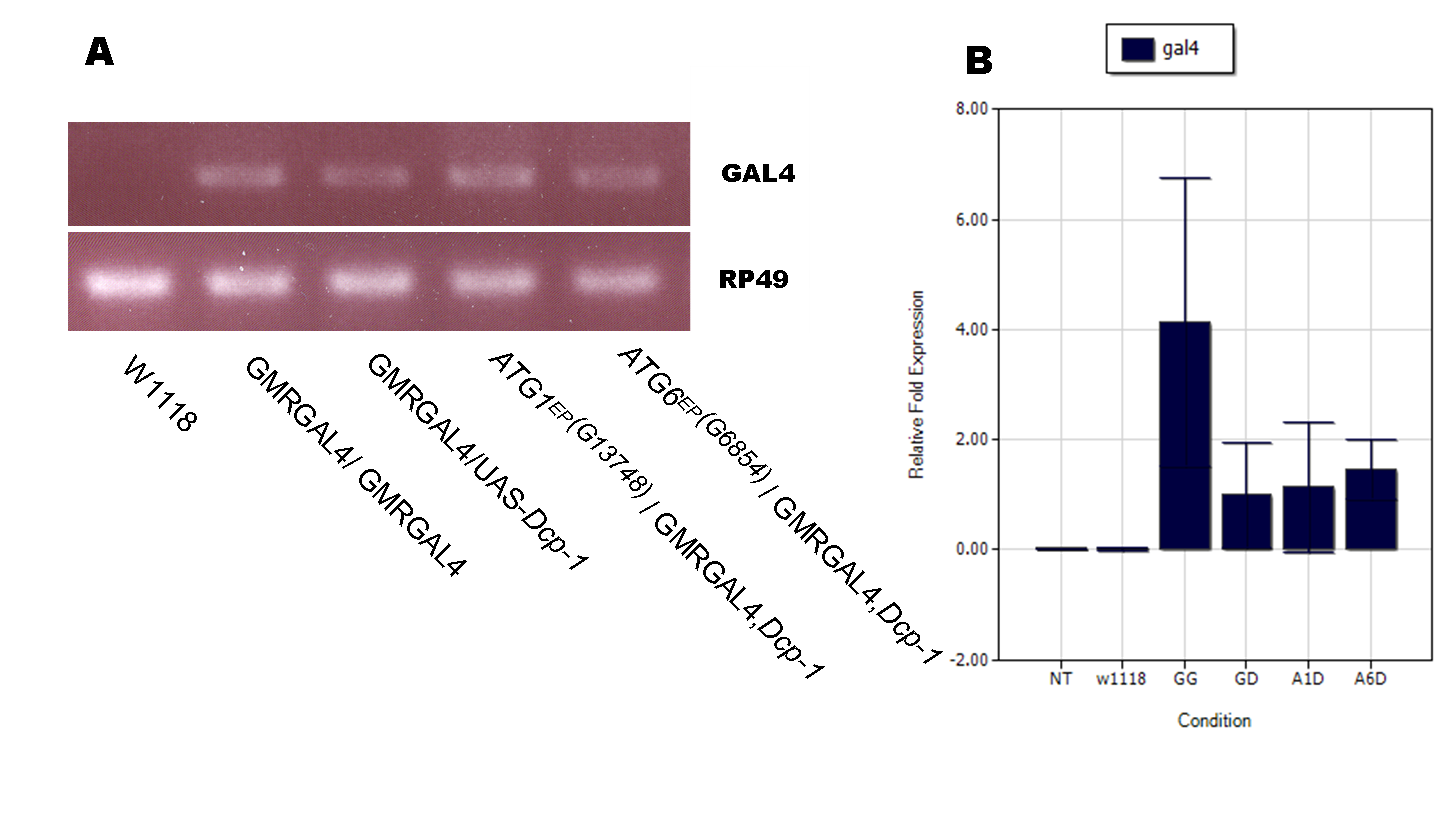

Supplement: Additional file 2 — GAL4 mRNA expression level was constant in every GAL4 mediated line. (A) The level of GAL4 expression was not detected in the w1118. The level of GAL4 expression was constant in every GAL4 mediated line. (B) The expression level of GAL4 was compared with that of RP49 by using the real-time PCR experiment. (NT: No template/GG: GMR-GAL4, homozygote/GD: Dcp-1 GF/CyO/A1D: Atg1EP(G13748)/Dcp-1 GF/A6D: Atg6EP(G6854)/Dcp-1 GF) [file 1471-2121-11-9-S2.TIFF]

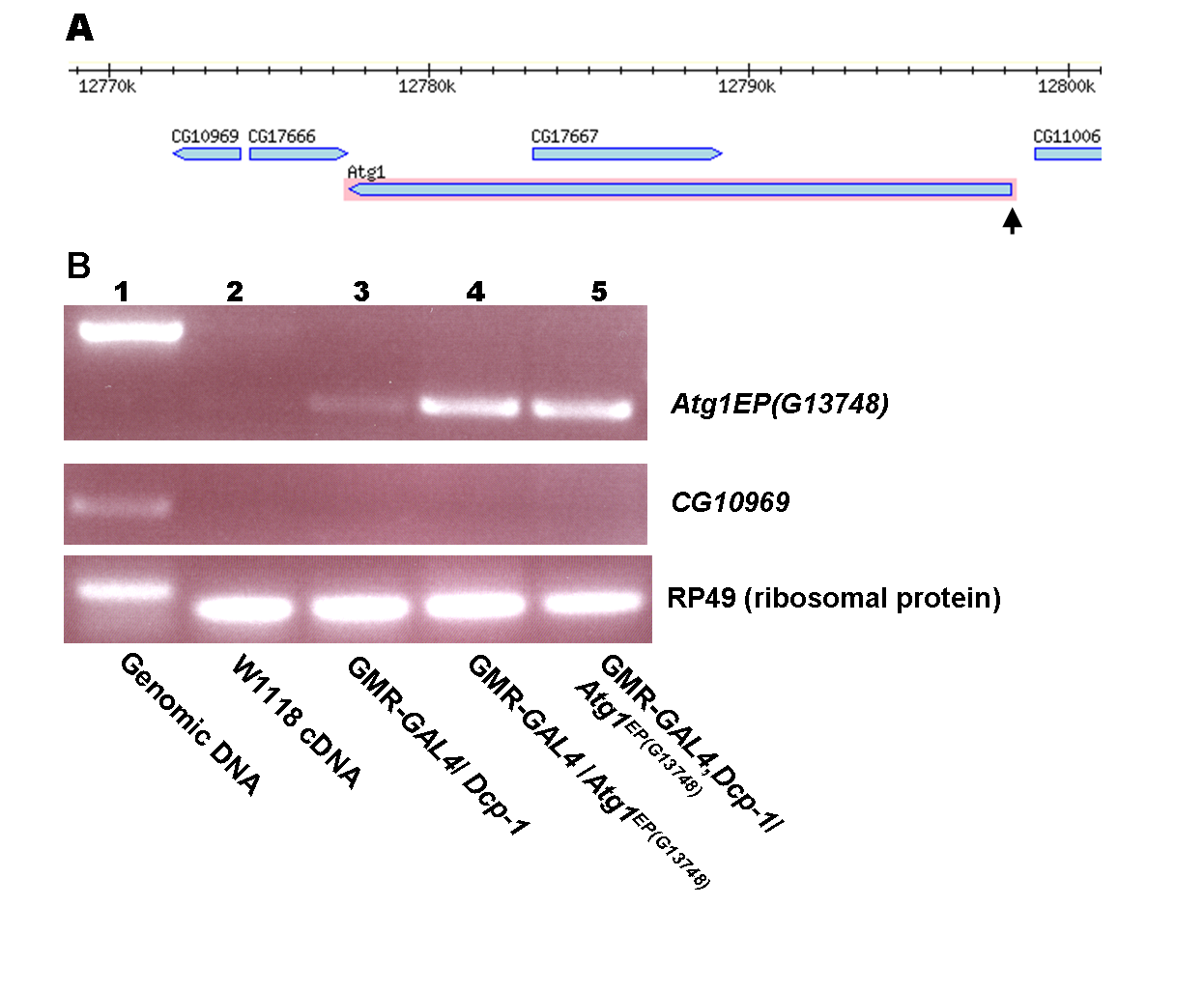

Supplement: Additional file 3 — Expression level of CG10969, down-stream gene of Atg1, was not affected by EP inserted up-stream of Atg1. (A) The surrounding genes of Atg1. CG10969, CG17666, CG17667 and CG11006 are located next to the Atg1. However, the directions of CG17666, CG17667 and CG11006 are opposite to the direction of EP (G13748) insertion. CG10969 is placed in down-stream site of Atg1 and EP (G13748). (Black arrow indicates the P-element insertion site of Atg1EP(G13748).). (B) The genomic DNA of w1118 was used as a template for the lane 1, while appropriate cDNAs were used for the lanes 2, 3, 4 and 5. The lane 1 indicates that all the primer sets worked properly. The lane 2 indicates that Atg1 and CG10969 were not expressed in the w1118. The lane 3 indicates that Atg1 was slightly up-regulated by the expression of Dcp-1. The lane 4 and 5 indicates that Atg1 expression level was increased in GMR-GAL4/Atg1EP(G13748) and GMR-GAL4, Dcp-1/Atg1EP(G13748). No expression was detected by the CG10969 primer set. [file 1471-2121-11-9-S3.TIFF]

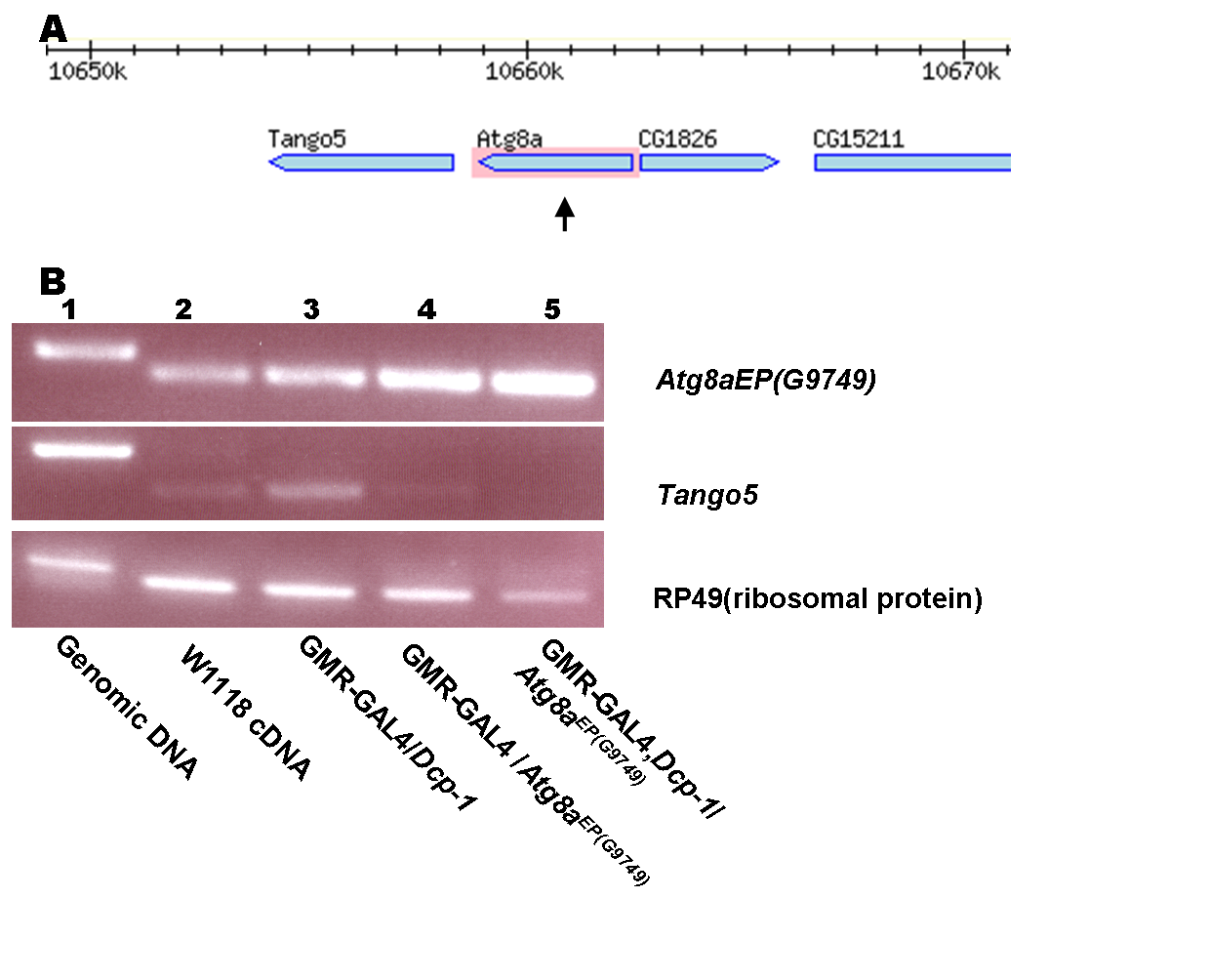

Supplement: Additional file 4 — Expression level of Tango5, down-stream gene of Atg8a, was not affected by EP inserted up-stream of Atg8a. (A) The surrounding genes of Atg8a. CG1826 and CG15221 are located up-stream site of Atg8a. Tango5 is located down-stream of Atg8a and EP (G9749). (Black arrow indicates P-element insertion site of Atg8aEP(G9749)). (B) Genomic DNA of w1118 was used as a template for the lane 1, while appropriate cDNAs were used for the lanes 2, 3, 4 and 5. The lane 1 indicates that all the primer sets worked properly. The lane 2 indicates that Atg8a and Tango5 were expressed in the w1118 as the baseline level. The lane 3 indicates that Atg8a was up-regulated by the expression of Dcp-1. As showed in the lane 4 and 5,Atg8a expression level was increased in GMR-GAL4/Atg8aEP(G9749) and GMR-GAL4, Dcp-1/Atg8aEP(G9749). The level of Tango5 expression was detected slightly in the Lane 2, 3 and 4. However, Tango5 expression level was not increased by Atg8aEP(G9749). [file 1471-2121-11-9-S4.TIFF]

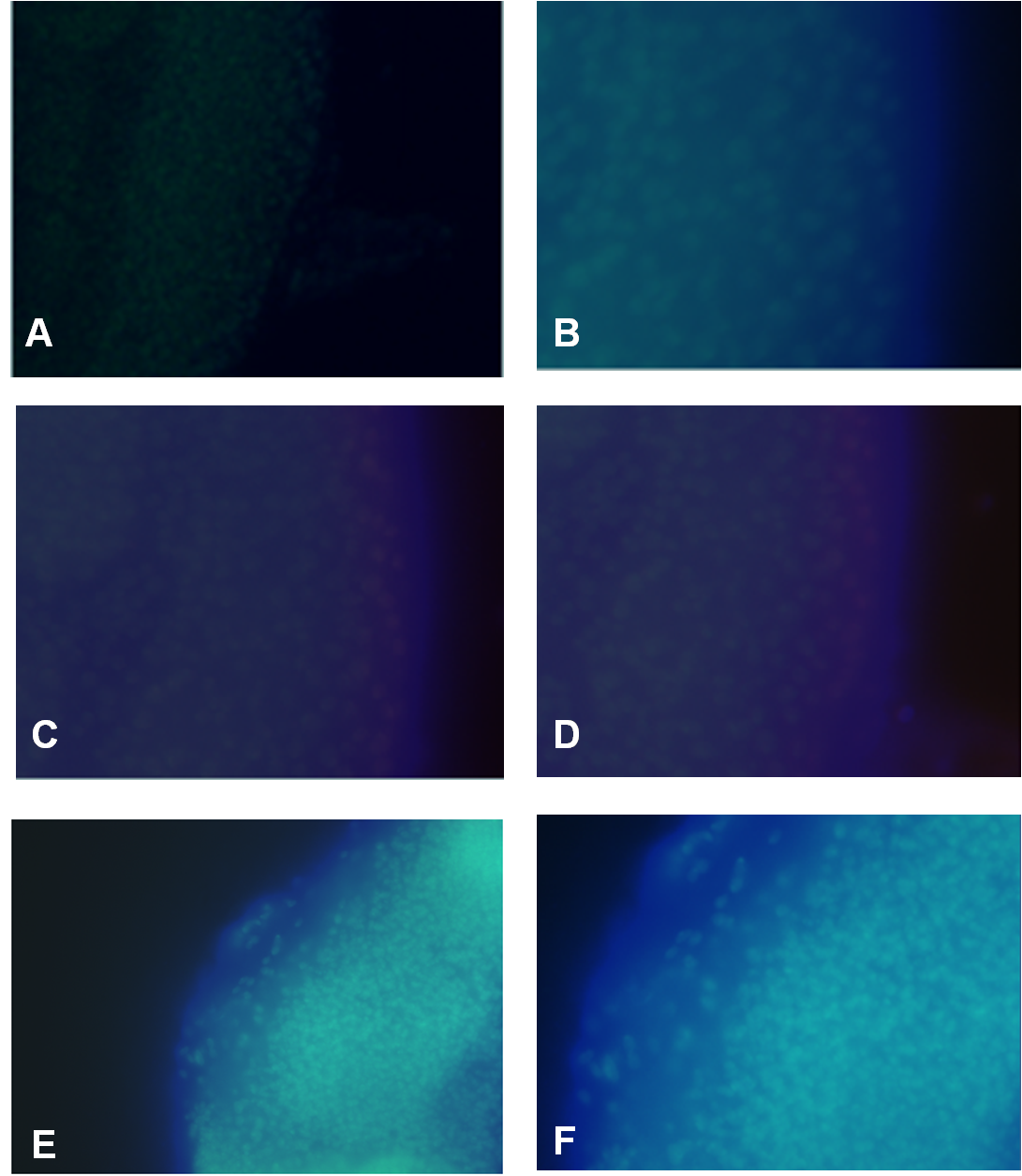

Supplement: Additional file 5 — eGFP-Atg5 and Atg8b-GFP were expressed by GMR-GAL4 in imaginal discs. (A) Merged image of GFP expression pattern and Höechst 33342 stained pattern in eye imaginal disc of a GMR-GAL4;UAS-eGFP-Atg5. (B) eGFP-Atg5 expression pattern in eye imaginal disc of a GMR-GAL4;UAS-eGFP-Atg5. (B, C and D) Higher-magnification images of (A). (C) LysoTracker Red spots. (D) LysoTracker Red spots and eGFP-Atg5 spots were overlapped (white arrows indicate autophagosome). (E and F) Merged image of GFP expression pattern and Höechst 33342 stained pattern in eye imaginal disc of Hs-Atg8b-GFP larva after heat shock. (12 hour after five 1-hour heat shocks). [file 1471-2121-11-9-S5.TIFF]

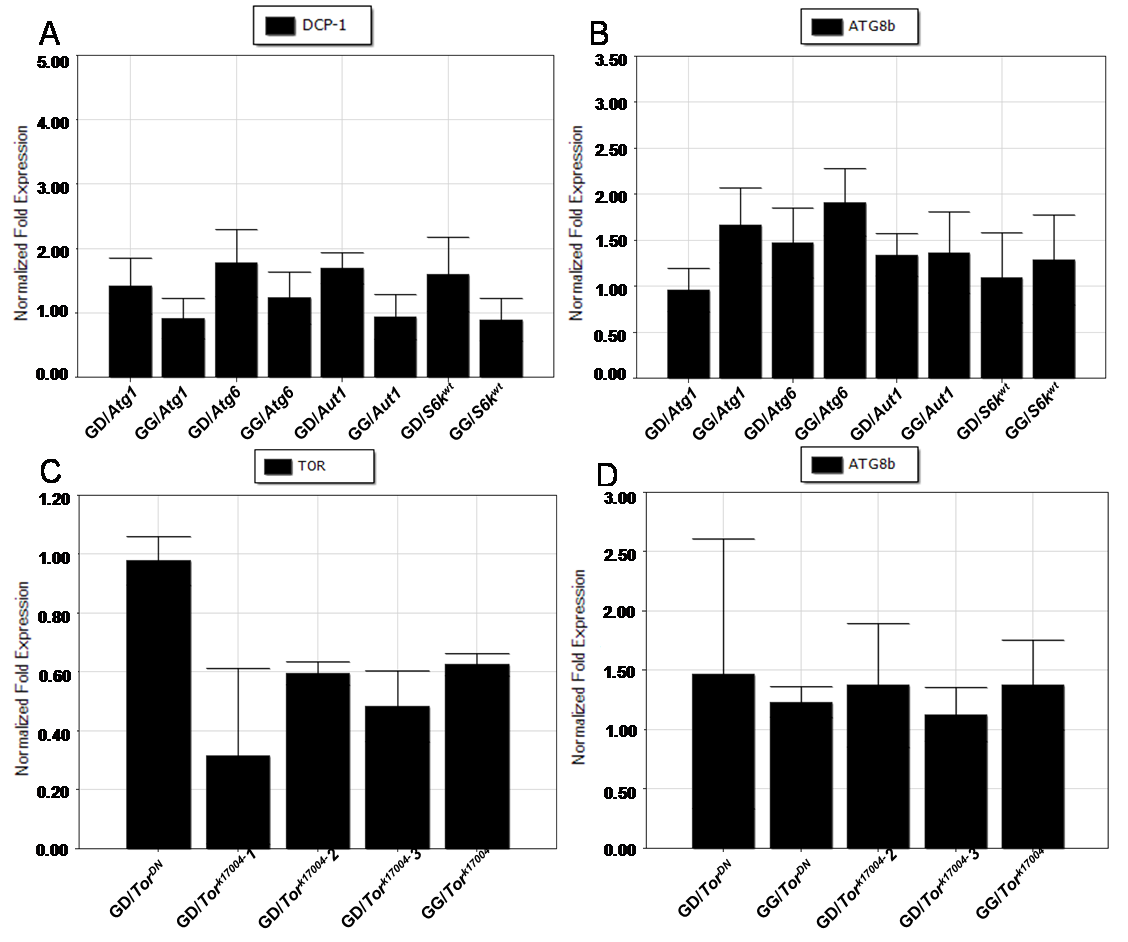

Supplement: Additional file 7 — Expression level test by real time PCR. (A) Dcp-1 expression level was increased in Dcp-1 GF flies that crossed with autophagy-related EP strains. (B) Atg8b expression level was increased in autophagy strains that crossed with GMR-GAL4 or Dcp-1 GF. (C) Tor expression level was reduced ~60% in, Tork17004/Dcp-1 GF and Tork17004/GMR-GAL4, Tor mutants. However, the expression level was not changed in TorDN/Dcp-1 GF line. (D) Atg8b expression level was increased in each of the Tor mutants. Atg8b expression level was slightly increased in the Tor mutants crossed with Dcp-1 GF than that of the flies crossed with GMR-GAL4. At least three individual experiments for each sample were put together for the gene expression study function in IQ5 2.0 and the expression levels were normalized to the reference gene RP-49. The control sample was obtained from w1118 wild-type flies. The error bars represent the SD of ddCt value. GD indicates GMR-GAL4;UAS-Dcp-1 and GG indicates GMR-GAL4. [file 1471-2121-11-9-S7.TIFF]
