# Supplementary material for: A genetic screen for modifiers of Drosophila caspase Dcp-1 reveals caspase involvement in autophagy and novel caspase-related genes
Source: BMC Cell Biol. 2010 Jan 25;11:9. doi: 10.1186/1471-2121-11-9 (PMC2822743; doi:10.1186/1471-2121-11-9)
Supplement: Additional file 6 — List of positive candidates from the screen. This file was originally made by using the MS Access and transformed to the PDF format provided here. [file 1471-2121-11-9-S6.PDF]

Number	source
9	My lab

line number	holding
Dronc	☑

chromosome	Insertion site
0	

crossGMRDCP-1	crossGMRGAL4
ND	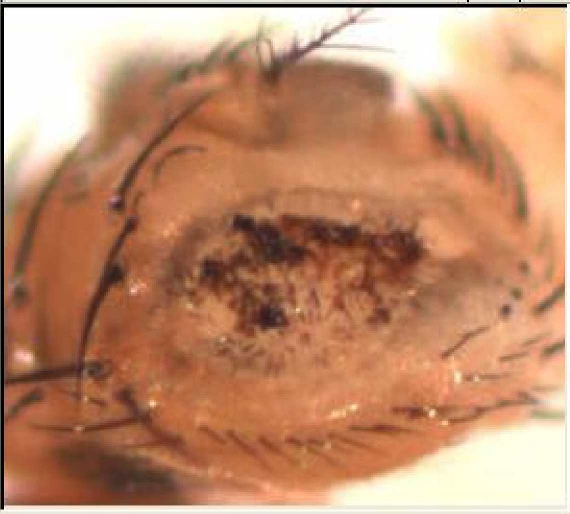

comments

Lethal(dronc 1-1,3-1,5-1,7-1,9-2,11-1,13-3,19-2)

similar phenotype

gene

Dronc

gene function

Number
10

source
My lab

line number
Dredd 25-1

holding
☑

chromosome
0

Insertion site

crossGMRDCP-1

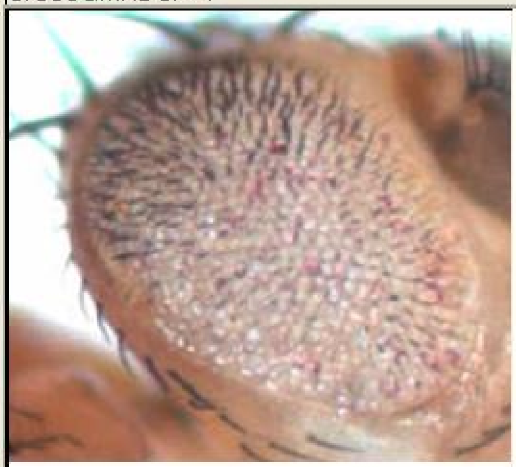

crossGMRGAL4

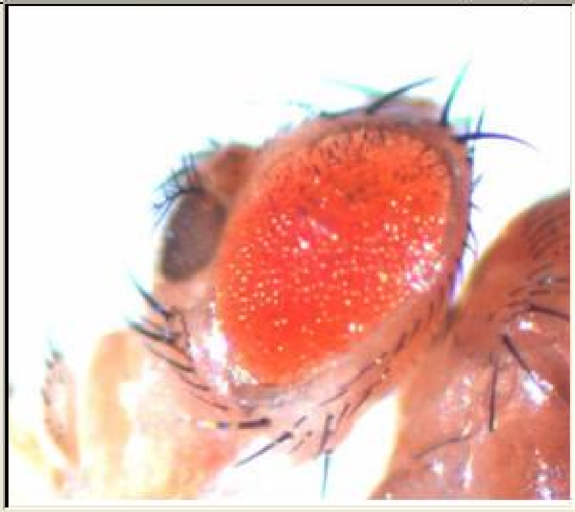

comments
reduced pigment.
Gmr-dredd25-1

similar phenotype

gene
Dredd

gene function

Number
11

source
My lab

line number
gmrPLZF

holding
☑

chromosome
0

Insertion site

crossGMRDCP-1

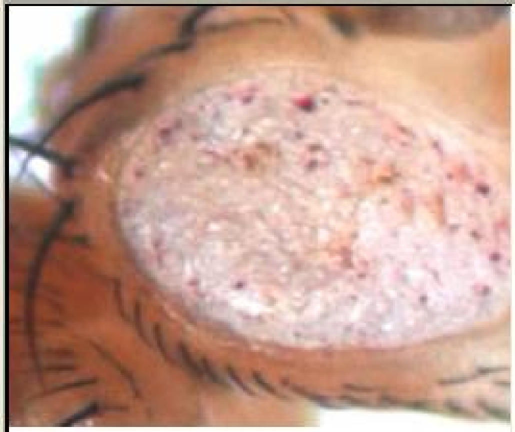

crossGMRGAL4

comments
rough eye, black spots.

similar phenotype

gene
PLZF

gene function

Number
12

source
My lab

line number
gmrHid

holding
☑

chromosome
0

Insertion site

crossGMRDCP-1

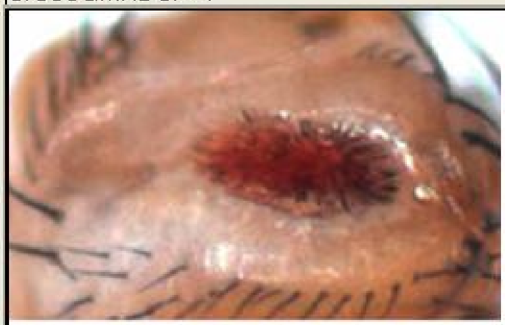

crossGMRGAL4

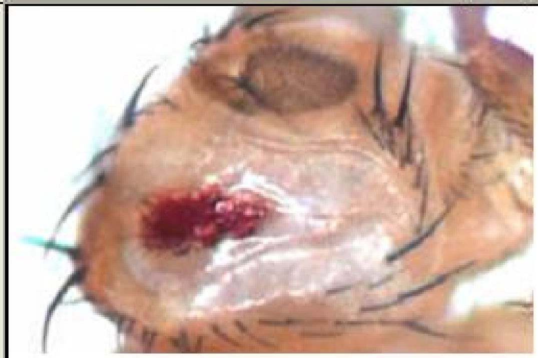

comments
Hid and DCP-1:Hid shows same phenotype.
Fig1: gmr-hid X DCP-1 19-2 (gmr,hid,dcp-1 1 copy each)
fig2: gmr-hid only

similar phenotype

gene
Hid

gene function

Number	source
13	My lab

line number	holding
UAS-rpr(2)	☑

chromosome	Insertion site
0	

crossGMRDCP-1	crossGMRGAL4
Lethal	Lethal

comments

Lethal,   gmr-rpr/tm6b   lethal
UAS-rpr(2) * GMR-dcp-1 raise in 18'C= Lethal

similar phenotype

gene

rpr

gene function

Number	source
14	signaling stocks

line number	holding
UAS-p35(3)	☑

chromosome	Insertion site
0	

crossGMRDCP-1	crossGMRGAL4
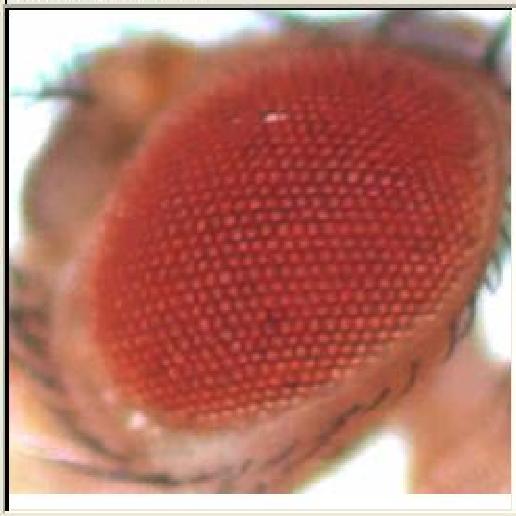	

comments

completely rescued

similar phenotype

gene

p35

gene function

Number	source
15	signaling stocks

line number	holding
BL6291 UAS-Rac1 v12(3)	☑

chromosome	Insertion site
0	

crossGMRDCP-1	crossGMRGAL4
Lethal	

comments

Lethal(all cyo red)

similar phenotype

gene

Rac1

gene function

Molecular function : Rho small monomeric GTPase

Biological process : cell proliferation, rhabdomere development

Number	source
17	signaling stocks

line number	holding
BL6292 UAS-Rac1N17	☑

chromosome	Insertion site
0	

crossGMRDCP-1	crossGMRGAL4
Lethal	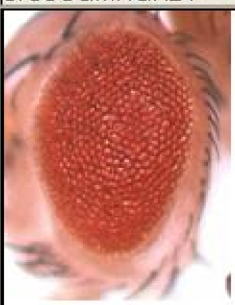

comments

Lethal
gmr little bit rough

similar phenotype

gene

Rac1

gene function

Molecular function : Rho small monomeric GTPase

Biological process : cell proliferation, rhabdomere development

Number	source
18	signaling stocks

line number	holding
yw;UAS-map2c	☑

chromosome	Insertion site
0	

crossGMRDCP-1	crossGMRGAL4
	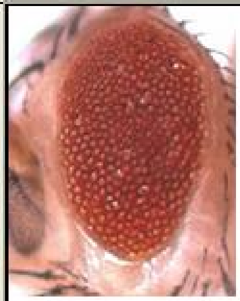

comments

small eye
gmr little bit small and rough

similar phenotype

gene

map2c

gene function

Number	source
19	signaling stocks

line number	holding
UAS-FMR1 (2)	☑

chromosome	Insertion site
0	

crossGMRDCP-1	crossGMRGAL4
Lethal	Lethal

comments

lethal

similar phenotype

gene

gene function

Number
21

source
signaling stocks

line number
UAS-p53/cyo

holding
☑

chromosome
2

Insertion site

crossGMRDCP-1

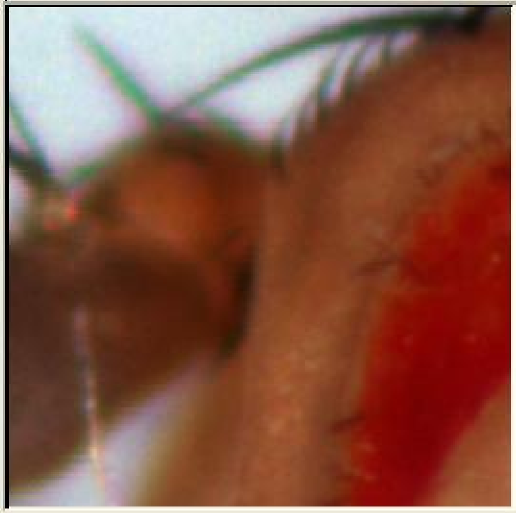

crossGMRGAL4

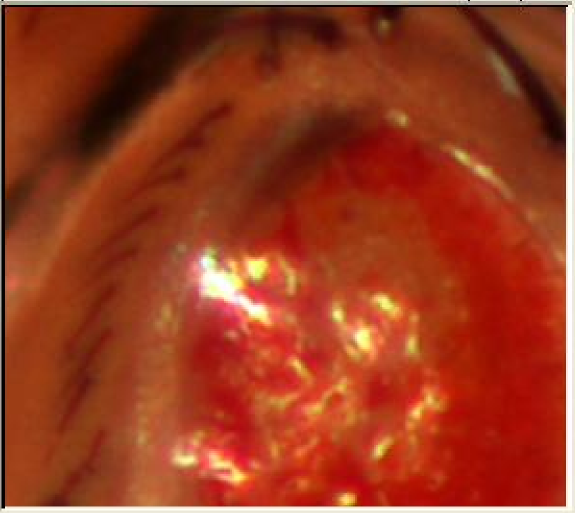

comments
slenderize, red

similar phenotype

gene

gene function

Number
22

source
signaling stocks

line number
w;UAS-PKAC/TM3

holding
☑

chromosome
0

Insertion site

crossGMRDCP-1

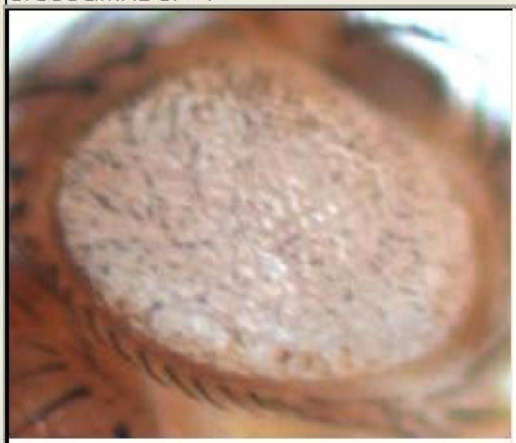

crossGMRGAL4

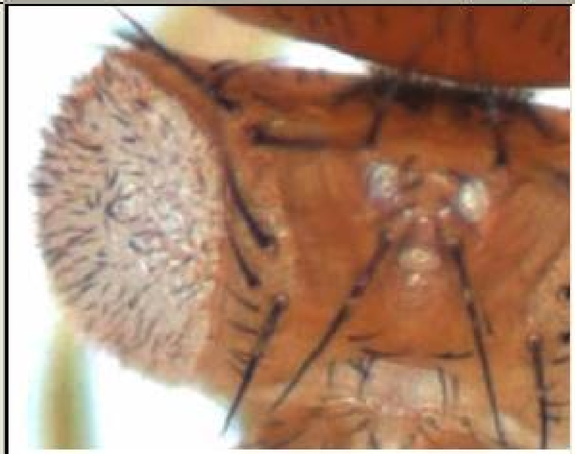

comments
bigger eye, pigment lost, rough
fig2- Not GMRGAL4 crossed line, same shot from fig1

similar phenotype

gene
PKAC

gene function

Number
23

source
signaling stocks

line number
EP381(faf)

holding
☑

chromosome
0

Insertion site

crossGMRDCP-1

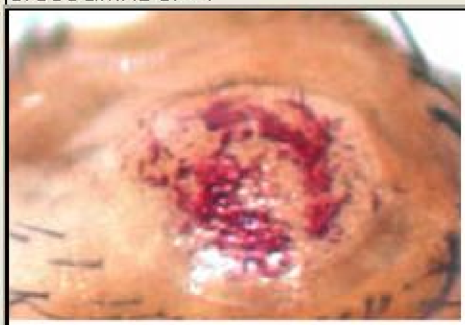

crossGMRGAL4

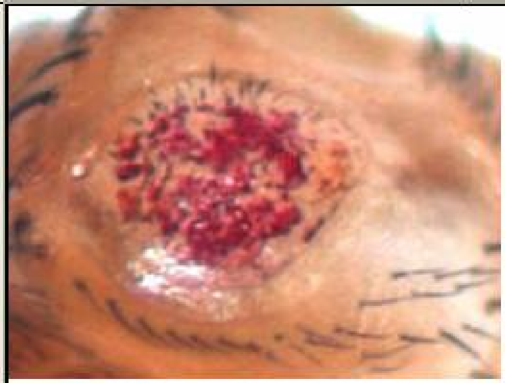

comments
small eye,
Fig2, Not GMRGAL4 crossed line, shot from another line.

similar phenotype

gene

gene function

Number	source
24	signaling stocks

line number

UAS-sgg(poor)

holding

☑

chromosome	Insertion site
0	

crossGMRDCP-1	crossGMRGAL4
Lethal	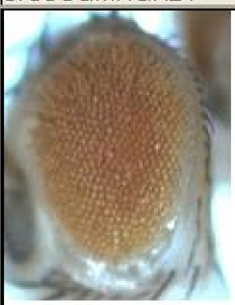

comments

lethal

gmr slighly rough

similar phenotype

gene

sgg(shaggy)

gene function

M : protein serine/threonine kinase

B : circadian rhythm, segment polarity determination

Field	Value
Number	25
source	signaling stocks
line number	UAS-p35(2)
holding	☑
chromosome	0
Insertion site	
crossGMRDCP-1	completely rescued
crossGMRGAL4	ND
comments	completely rescued
similar phenotype	UAS-p35(3)
gene	
gene function	

Number	source
28	signaling stocks

line number	holding
EP(3)3517	☑

chromosome	Insertion site
3	

crossGMRDCP-1	crossGMRGAL4
same as UAS-Dfmr1(144N)	ND

comments

small eye , glazed

similar phenotype

UAS-Dfmr1(144N)

gene

gene function

Number	source
29	signaling stocks

line number	holding
UAS-Dfmr1(1307N)	☑

chromosome	Insertion site
0	

crossGMRDCP-1	crossGMRGAL4
Lethal	ND

comments

Lethal

similar phenotype

gene

gene function

Number
30

source
signaling stocks

line number
UAS-dFMR1(144N)

holding
☑

chromosome
0

Insertion site

crossGMRDCP-1

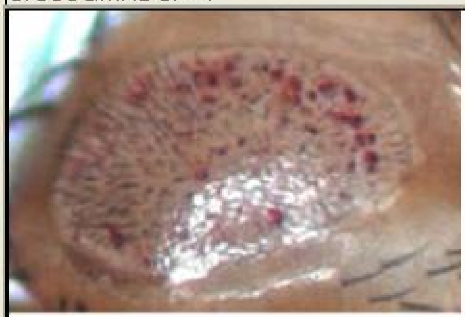

crossGMRGAL4

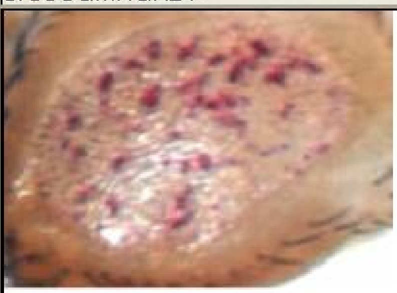

comments
small eye, glazed, flattened
fig2: Not GMRGAL4 crossed line, same line as Fig1

similar phenotype

gene

gene function

Number	source
31	signaling stocks

line number	holding
BL5078 FRT14A-B/FM7a ; en(e22c)>FLP #X,2	☑

chromosome	Insertion site
0	

crossGMRDCP-1	crossGMRGAL4
Lethal	ND

comments

Lethal

similar phenotype

gene

gene function

Number	source
32	signaling stocks

line number	holding
BL5079 FRT14A-B;T155>FLP #X,3	☑

chromosome	Insertion site
0	

crossGMRDCP-1	crossGMRGAL4
Lethal	ND

comments

Lethal

similar phenotype

gene

gene function

Number	source
35	signaling stocks

line number	holding
BL6343 yw; eyeFLP/CyO ; FRT79D,GMR-hid/TM6,y[+]	☑

chromosome	Insertion site
0	

crossGMRDCP-1	crossGMRGAL4
Lethal	ND

comments

Lethal

similar phenotype

gene

gene function

Number	source
40	signaling stocks

line number	holding
yw; UAS-Hraf	☑

chromosome	Insertion site
0	

crossGMRDCP-1	crossGMRGAL4
Lethal	ND

comments

Lethal

similar phenotype

gene

gene function

Number	source
41	signaling stocks

line number	holding
BL2033 UAS-Draf c.a #3	☑

chromosome	Insertion site
0	

crossGMRDCP-1	crossGMRGAL4
Lethal	ND

comments

Lethal

similar phenotype

gene

gene function

Number
42

source
signaling stocks

line number
BL5368 UAS-Egfr #3

holding
☑

chromosome
0

Insertion site

crossGMRDCP-1

crossGMRGAL4

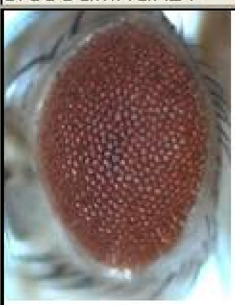

comments
pigment rescued, glazed

gmrgal4 cross= slightly rough

similar phenotype

gene

gene function

Number	source
43	signaling stocks

line number	holding
BL5364 UAS-Egfr DN #2,3	☑

chromosome	Insertion site
0	

crossGMRDCP-1	crossGMRGAL4
Lethal	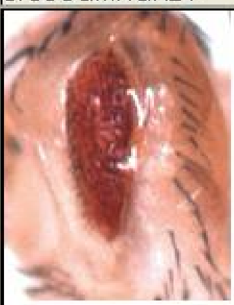

comments

Lethal?
Gmrgal4 crosss= slenderized red

similar phenotype

gene

gene function

Number
44

source
signaling stocks

line number
UAS-Dfosbzip

holding
☑

chromosome
0

Insertion site

crossGMRDCP-1

Lethal

crossGMRGAL4

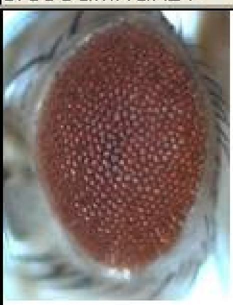

comments
semilethal?
Gmrgal4 cross=slightly rough

similar phenotype

gene
kayak(dfos)

gene function
M : DNA binding, specific RNA polymerase II transcription factor;

B : follicle cell migration, response to wounding

similar phenotype as egfr
bZIP (Basic-leucine zipper transcription factor.
One of the domain of Kayak

Number
45

source
signaling stocks

line number
UAS-RasV12 #3

holding
☑

chromosome
0

Insertion site

crossGMRDCP-1
Lethal

crossGMRGAL4

comments
Lethal

similar phenotype

gene

gene function

Number
46

source
signaling stocks

line number
UAS-rho #X

holding
☑

chromosome
0

Insertion site

crossGMRDCP-1

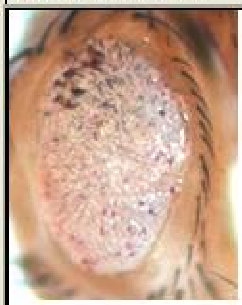

crossGMRGAL4

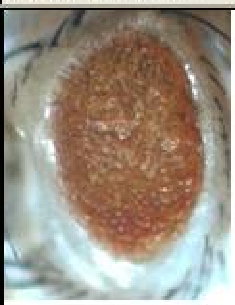

comments
pigment lost, rough, small eye

similar phenotype

gene

gene function
rho : EGF receptor ligand processing, peptidolysis

Number	source
47	signaling stocks

line number: UAS-rho #3

holding: ☐

chromosome: 0

Insertion site:

crossGMRDCP-1	crossGMRGAL4
same as UAS-rho #x	same as UAS-rho #x

comments

same as UAS-rho #x

similar phenotype

UAS-rho #x

gene

gene function

Number
48

source
signaling stocks

line number
BL6288 UAS-Cdc42, N17 #2

holding
☑

chromosome
0

Insertion site

crossGMRDCP-1

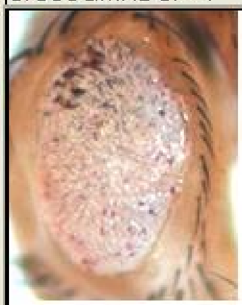

crossGMRGAL4

comments
same as UAS-rho #x

similar phenotype
UAS-rho #x

gene

gene function
cdc42 : Rho small monomeric GTPase…

Number	source
49	signaling stocks

line number	holding
BL6272 UAS-Rac1, N17 #3	☑

chromosome	Insertion site
0	

crossGMRDCP-1	crossGMRGAL4
Lethal	

comments

Lethal

similar phenotype

gene

gene function

Number	source
60	signaling stocks

line number	holding
BL5613 TM3[UAS-DIDN]Sb	☑

chromosome	Insertion site
0	

crossGMRDCP-1

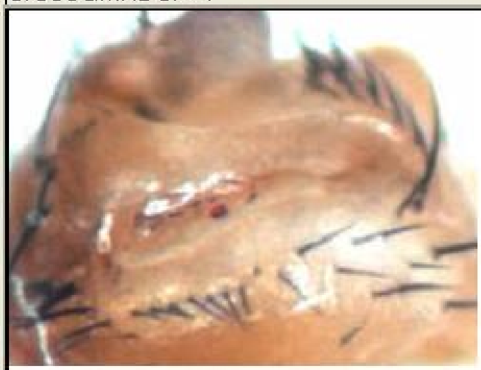

crossGMRGAL4

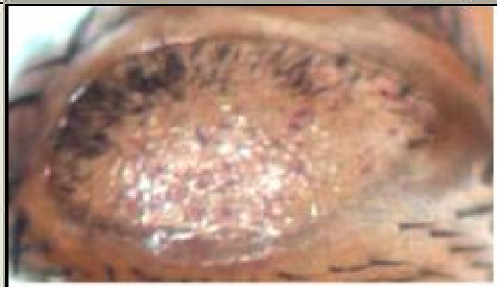

comments

slenderized, pigment lost, hairless.

similar phenotype

gene

gene function

Number
62

source
signaling stocks

line number
ΔTor2J-12

holding
☑

chromosome
0

Insertion site

crossGMRDCP-1

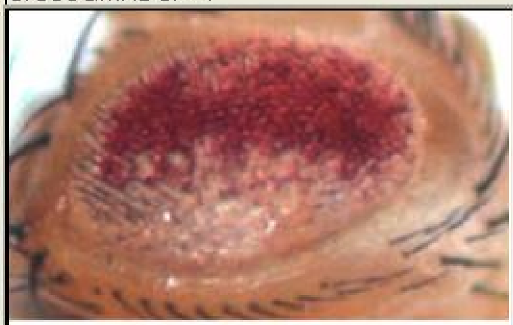

crossGMRGAL4

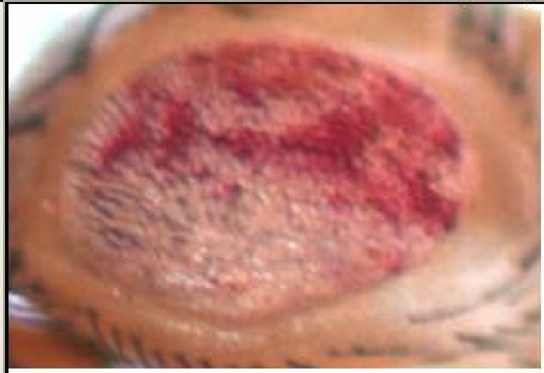

comments
partial rescued
fig2- Not GMRGAL4 crossed line, shot from same line as fig1

similar phenotype

gene

gene function

Number	source
81	signaling stocks

line number	holding
yw; UAS-hepCA (wing trap) #3	☑

chromosome	Insertion site
0	

crossGMRDCP-1	crossGMRGAL4
Lethal	normal

comments

Lethal

similar phenotype

gene

gene function

Number	source
82	signaling stocks

line number	holding
UAS-DJNKDN/FM7C	☑

chromosome	Insertion site
0	

crossGMRDCP-1	crossGMRGAL4
Lethal	Normal eye

comments

Lethal

similar phenotype

gene

basket(jnk)

gene function

M : protein serine/threonine kinase, JUN kinase, MAP kinase

B : JNK cascade, antibacterial humoral response (sensu Invertebrata), wound healir

Number	source
85	signaling stocks

line number	holding
UAS-dTAK1 DK4 #3	☑

chromosome	Insertion site
0	

crossGMRDCP-1	crossGMRGAL4
Lethal	Normal eye

comments

cyo, white : cyo,red: straight, gD eye = 1:1:1 => lethal

similar phenotype

p35

gene

dTak1(TGF-beta activated kinase 1)

gene function

M : protein kinase, MAP kinase kinase kinase

B : JNK cascade, apoptosis

Number
86

source
signaling stocks

line number
w; UAS-DMEKKIa3-5/TM3

holding
☑

chromosome
0

Insertion site

crossGMRDCP-1

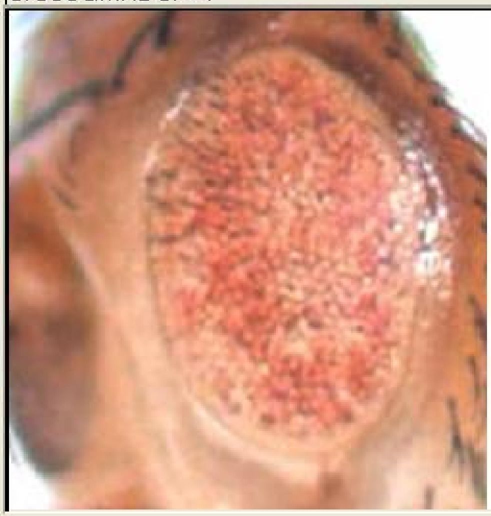

crossGMRGAL4

Normal eye

comments
small eye

similar phenotype

gene
Mekk1

gene function
M : receptor signaling protein serine/threonine kinase, MAP kinase kinase kinase

B : protein amino acid phosphorylation

Number	source
87	signaling stocks

line number	holding
yw; UAS-hepCA #3	☑

chromosome	Insertion site
0	

crossGMRDCP-1

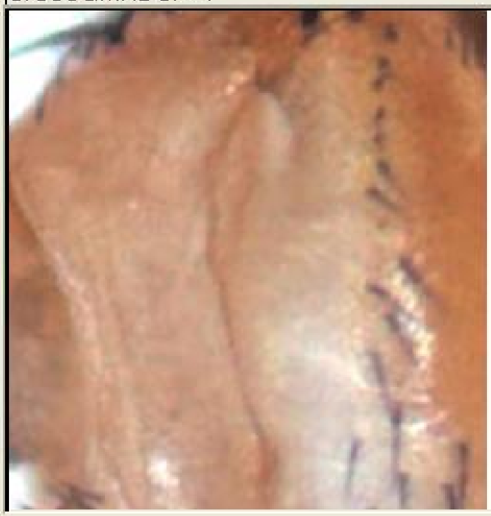

crossGMRGAL4

comments

rare progeny, almost lethal.

similar phenotype

gene

gene function

Gd cross= almost lethal

Number	source
88	signaling stocks

line number	holding
UAS-Akt #3	☑

chromosome	Insertion site
0	

crossGMRDCP-1

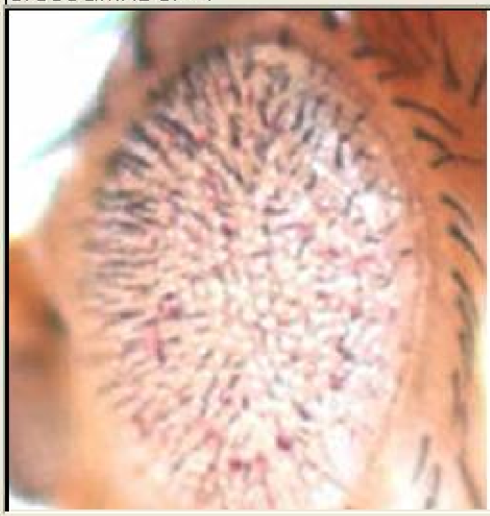

crossGMRGAL4

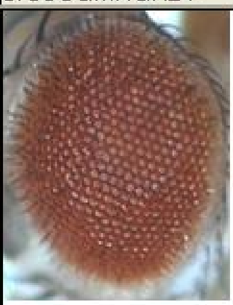

comments

pigment lost, rough eye
gmrgal4 crossed line= bigger eye

similar phenotype

gene

Akt

gene function

M : protein serine/threonine kinase, protein kinase

B : anti-apoptosis

similar phenotype as PKAC

Number	source
92	signaling stocks

line number	holding
w; UAS-P-DN-PVR/CyO	☑

chromosome	Insertion site
0	

crossGMRDCP-1	crossGMRGAL4
Lethal	Normal eye

comments

Lethal

similar phenotype

gene

PVR(PDGF- and VEGF-receptor related)

gene function

M : receptor signaling protein tyrosine kinase, vascular endothelial growth factor rec

B : actin cytoskeleton organization and biogenesis, border cell migration

Number	source
93	signaling stocks

line number	holding
w; UAS-λPVR-1/TM3SbSer	☑

chromosome	Insertion site
0	

crossGMRDCP-1

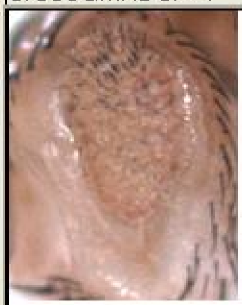

crossGMRGAL4

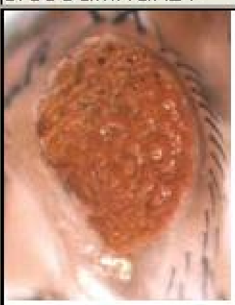

comments

small eye, pigment lost

similar phenotype

p35

gene

PVR(PDGF- and VEGF-receptor related)

gene function

M : receptor signaling protein tyrosine kinase, vascular endothelial growth factor rec

B : actin cytoskeleton organization and biogenesis, border cell migration

Number
94

source
signaling stocks

line number
yw;; UAS-dS6K "Fb2" #2

holding
☑

chromosome
0

Insertion site

crossGMRDCP-1
Lethal

crossGMRGAL4
Normal eye

comments
Lethal

similar phenotype

gene
S6k(RPS6-p70-protein kinase)

gene function
M : protein serine/threonine kinase, ribosomal protein S6 kinase

B : cell growth and/or maintenance, oogenesis

Number
98

source
signaling stocks

line number
UAS-PI3Kwt

holding
☑

chromosome
0

Insertion site

crossGMRDCP-1

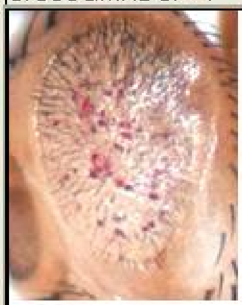

crossGMRGAL4

Normal eye

comments
small eye, glazed, flattened

similar phenotype

gene
phosphatidylinositol 3-kinase

gene function
M : phosphatidylinositol 3-kinase

B : phosphorylation

Number	source
99	signaling stocks

line number	holding
w; UAS-Pten/CyO ff20.2	☑

chromosome	Insertion site
0	

crossGMRDCP-1	crossGMRGAL4
Lethal	Normal eye

comments

Lethal

similar phenotype

gene

Pten

gene function

M : protein tyrosine/serine/threonine phosphatase

B : insulin receptor signaling pathway, protein amino acid dephosphorylation

Number
105

source
signaling stocks

line number
MKP/CyO ; TM2/TM6B #2

holding
☑

chromosome
0

Insertion site

crossGMRDCP-1

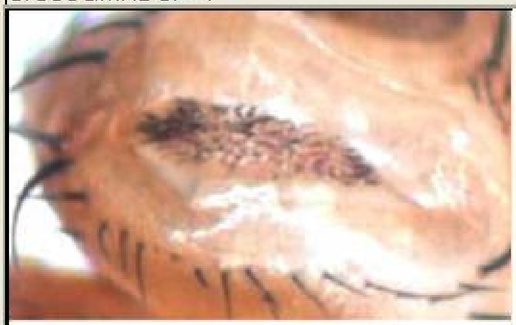

crossGMRGAL4
normal

comments
slenderized. Male lethal

similar phenotype

gene
MKP

gene function

Number
115

source
signaling stocks

line number
BL5844, UAS-Hsc70-4, D206S #2

holding
☑

chromosome
0

Insertion site

crossGMRDCP-1

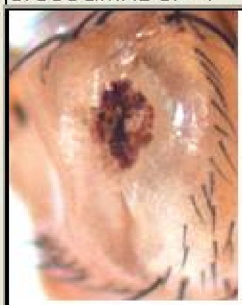

crossGMRGAL4

comments
small eye

similar phenotype

gene
Hsc70-4(Heat shock protein cognate 4)

gene function
M : heat shock protein

B : neurotransmitter secretion, response to heat shock, synaptic vesicle transport

Number

116

source

signaling stocks

line number

BL5845, UAS-Hsc70-4, K71S #2

holding

☑

chromosome

0

Insertion site

crossGMRDCP-1

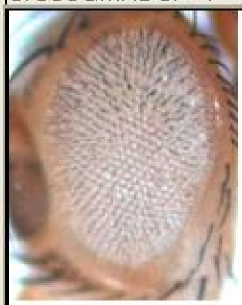

crossGMRGAL4

Yellow, normal eye

comments

pigment lost
gmrgal4 Cross yellowish normal

similar phenotype

gene

gene function

Number	source
123	signaling stocks

line number	holding
Ddc>GFP/CyO 1	☑

chromosome	Insertion site
0	

crossGMRDCP-1	crossGMRGAL4
Lethal	Normal eye

comments

lethal

similar phenotype

gene

Dopa decarboxylase

gene function

M : aromatic-L-amino acid decarboxylase

B : dopamine biosynthesis from tyrosine, serotonin biosynthesis from tryptophan

Number
149

source
signaling stocks

line number
EP(3)3091,FRT80B/TM6/TM2 2

holding

chromosome
0

Insertion site

crossGMRDCP-1

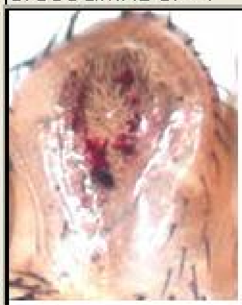

crossGMRGAL4

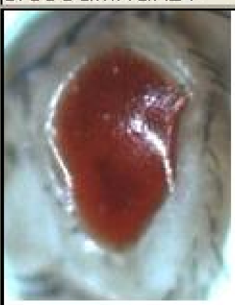

comments
small eye, fllatened.

similar phenotype

gene

gene function

Number
159

source
Szeged stock

line number
EP(X)1179

holding
☑

chromosome
1

Insertion site

crossGMRDCP-1	crossGMRGAL4
Lethal	Normal eye

comments
lethal

similar phenotype

gene
nej(nejire)

gene function
M : transcription co-activator, cAMP response element binding protein binding

B : synaptic vesicle transport

nej = CBP

Number
160

source
Szeged stock

line number
EP(3)3390

holding
☑

chromosome
3

Insertion site

crossGMRDCP-1

Lethal

crossGMRGAL4

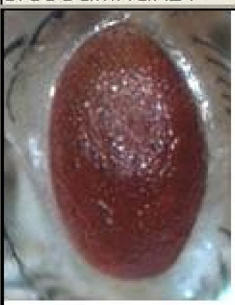

comments
cul-5 pupa lethal

similar phenotype

gene
cul-5

gene function
cul-5

Number
161

source
Szeged stock

line number
EP(3)3517#형가리

holding
☑

chromosome
3

Insertion site

crossGMRDCP-1

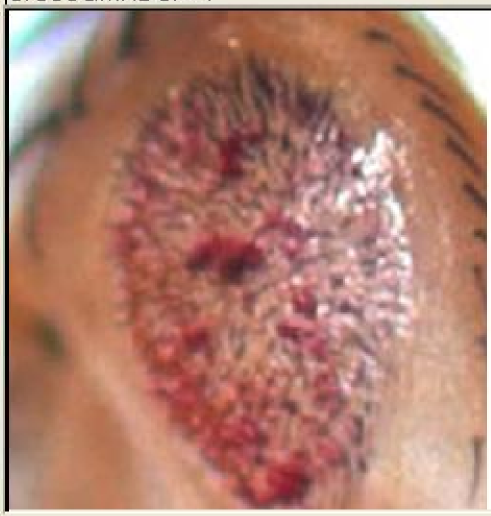

crossGMRGAL4

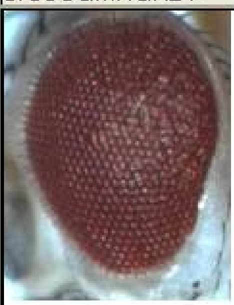

comments
small eye, reddish

similar phenotype

gene
fmr1

gene function
M : messenger RNA binding

B : translational repression

Number
162

source
Szeged stock

line number
EP(3)3520

holding
☑

chromosome
3

Insertion site

crossGMRDCP-1

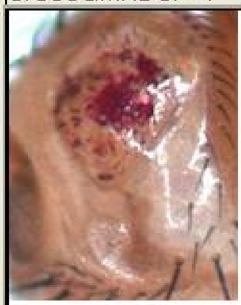

crossGMRGAL4

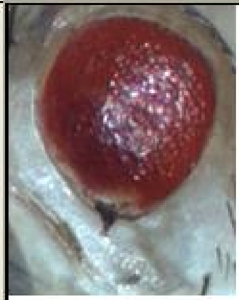

comments
small eye, reddish

similar phenotype

gene
faf(fat facets)

gene function
M : ubiquitin-specific protease

B : deubiquitylation, ubiquitin cycle

Number
163

source
Szeged stock

line number
EP(2)2299

holding
☑

chromosome
2

Insertion site

crossGMRDCP-1

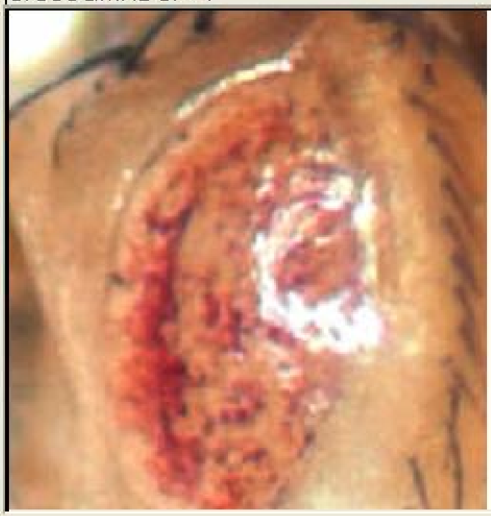

crossGMRGAL4

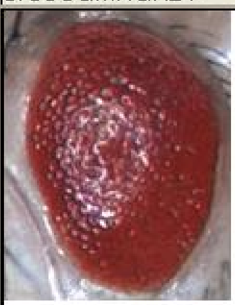

comments
small eye, glazed.

similar phenotype

gene
beach1

gene function
M : Unknown

B : axon에서 intracellular protein transport,lysosomal transport

Number	source
164	Szeged stock

line number	holding
EP(2)0316	☑

chromosome	Insertion site
2	

crossGMRDCP-1	crossGMRGAL4
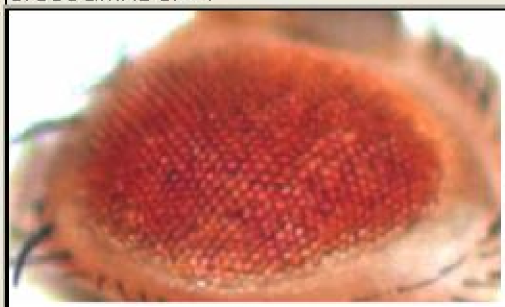	normal

comments

rescued

similar phenotype

gene

CG3065

gene function

CG3065 3'에 박힘

Field	Value
Number	165
source	Szeged stock
line number	EP(2)0594
holding	☑
chromosome	2
Insertion site	
crossGMRDCP-1	Lethal
crossGMRGAL4	Normal eye
comments	lethal
similar phenotype	
gene	CG4747
gene function	M : 3-hydroxyisobutyrate dehydrogenase<br>B : Unknown

Number

166

source

Szeged stock

line number

EP(2)0598

holding

☑

chromosome

2

Insertion site

crossGMRDCP-1

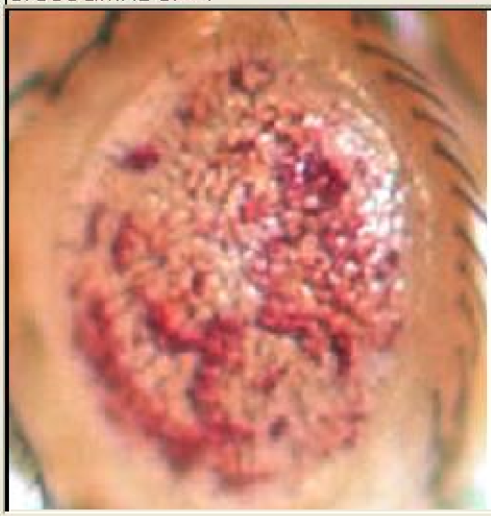

crossGMRGAL4

Normal eye

comments

glazed. Rounded

similar phenotype

gene

aop(anterior open)

gene function

M : specific RNA polymerase II transcription factor

B : induction of apoptosis, cell fate determination

Number
168

source
Szeged stock

line number
EP(2)2447

holding
☑

chromosome
2

Insertion site

crossGMRDCP-1	crossGMRGAL4
Lethal	Normal eye

comments
lethal

similar phenotype

gene
bun

gene function
M : RNA polymerase II transcription factor

B : cell fate determination, eye morphogenesis, oogenesis, peripheral nervous syste
development

Number
169

source
Szeged stock

line number
EP(2)2564

holding
☑

chromosome
2

Insertion site

crossGMRDCP-1

crossGMRGAL4

Normal eye

comments
pigment rescue

similar phenotype

gene
Cyclin E

gene function
M : cyclin-dependent protein kinase, regulator
B : Unknown

Number
173

source
Szeged stock

line number
EP(2)0670

holding
☑

chromosome
2

Insertion site

crossGMRDCP-1

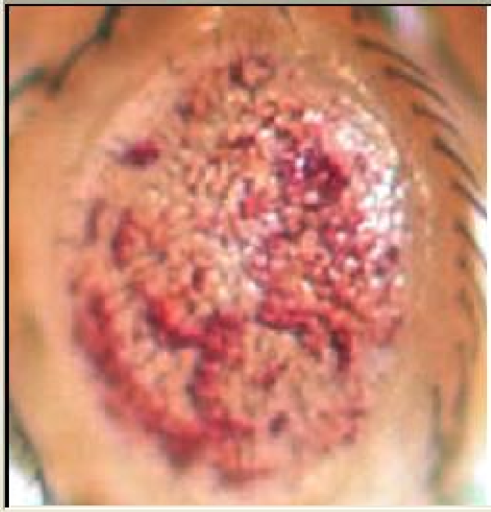

crossGMRGAL4

Normal eye

comments
same as EP(2)0598

similar phenotype
EP(2)0598

gene
Gst2

gene function
Gst2 5'
Gst2 : glutathione transferase

Number	source
174	Szeged stock

line number	holding
EP(3)3145	☑

chromosome	Insertion site
3	

crossGMRDCP-1

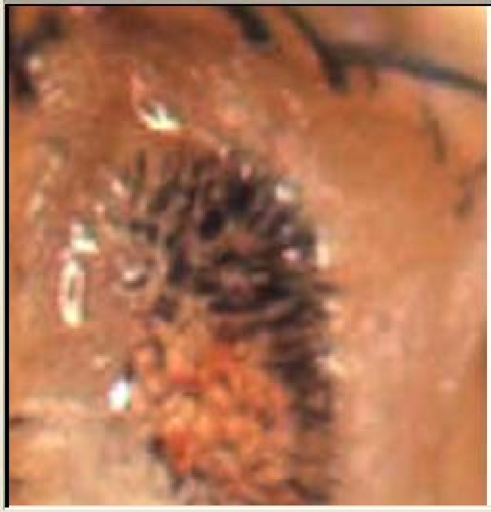

crossGMRGAL4

Normal eye

comments

pigment lost, crowded hair or lethal

similar phenotype

gene

CG5166 Ataxin-2, Atx2

gene function

Protein domain : Sm motif of small nuclear ribonucleoproteins, SNRNP

Number	source
175	Szeged stock

line number	holding
EP(3)3704	☑

chromosome	Insertion site
3	

crossGMRDCP-1	crossGMRGAL4
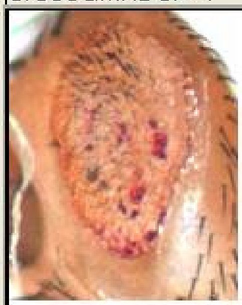	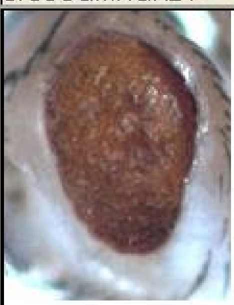

comments

similar phenotype

gene

rho(rhomboid)

gene function

M : serine-type peptidase
B : EGF receptor ligand processing, proteolysis and peptidolysis

Number	source
178	Szeged stock

line number	holding
EP(3)3279	☑

chromosome	Insertion site
3	

crossGMRDCP-1	crossGMRGAL4
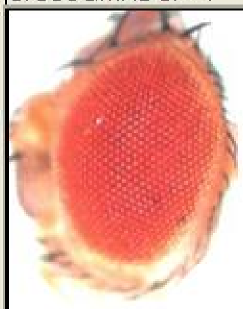	Normal eye

comments

completely rescued

similar phenotype

gene

th(thread=diap1)

gene function

apoptosis inhibitor

Number
179

source
Szeged stock

line number
EP(3)0381

holding
☑

chromosome
3

Insertion site

crossGMRDCP-1

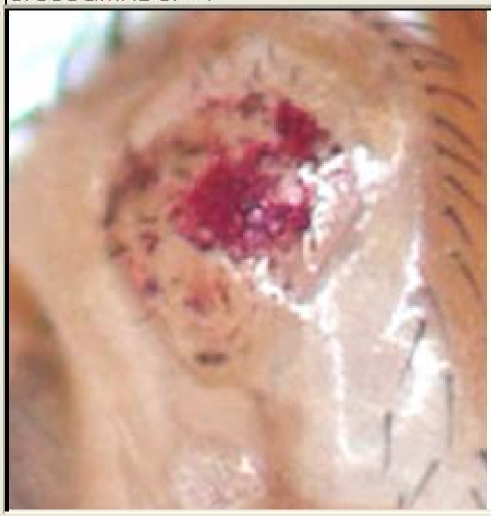

crossGMRGAL4

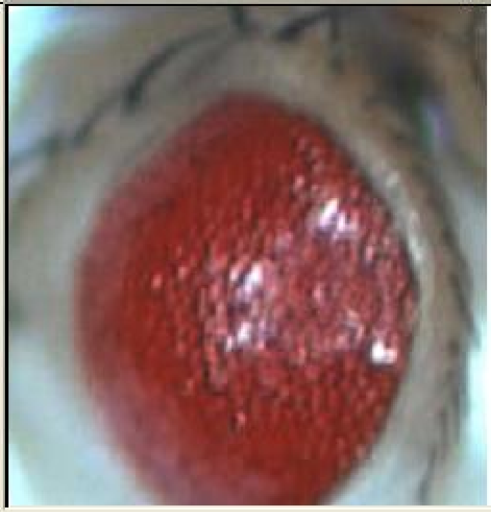

comments
same as EP(3)3520

similar phenotype
EP(3)3520

gene
faf

gene function
just beside 3520, both of 3520,0381 5' intron of faf

Number

192

source

Genexel stock

line number

GX79949

holding

chromosome

0

Insertion site

crossGMRDCP-1

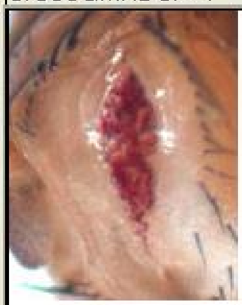

crossGMRGAL4

Normal eye

comments

slenderized, red, semilethal

similar phenotype

gene

gene function

Number
198

source
Genexel stock

line number
GX79989

holding

chromosome
0

Insertion site

crossGMRDCP-1

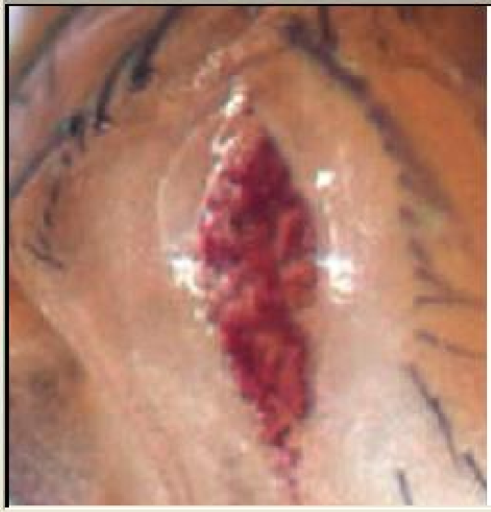

crossGMRGAL4

same as DCP-1

comments
slenderized, red, semilethal
gmrgal4= also slenderized, red, semilethal

similar phenotype

gene

gene function

Number	source
247	Genexel stock

line number	holding
GX79545	☐

chromosome	Insertion site
0	

crossGMRDCP-1	crossGMRGAL4
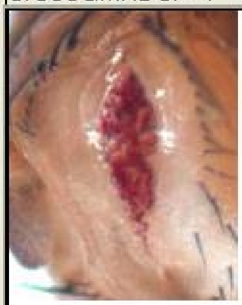	Normal eye

comments

slenderized, red

similar phenotype

gene

gene function

Number	source
252	Genexel stock

line number	holding
GX77223	☑

chromosome	Insertion site
0	AE003689 14585

crossGMRDCP-1	crossGMRGAL4
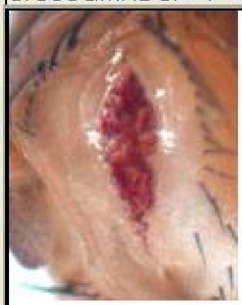	Normal eye

comments

slenderized, red
CG14696 14585

similar phenotype

gene

CG14696

gene function

Unknown

Number
260

source
Genexel stock

line number
GX83142 G4432

holding
☑

chromosome
3

Insertion site
AE003479 132804

crossGMRDCP-1

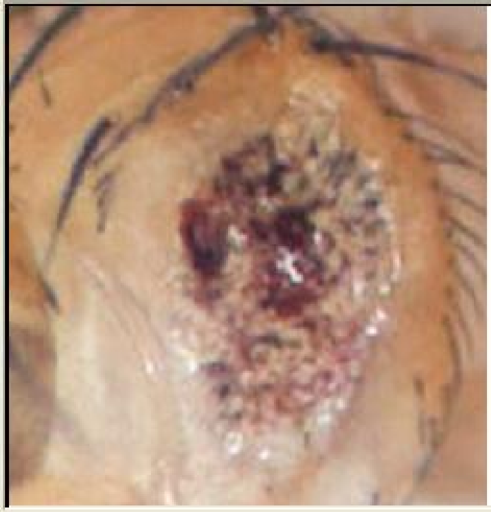

crossGMRGAL4

Normal eye

comments
small eye pigment lost
ENC 132804

similar phenotype

gene
enc

gene function

Number
272

source
Genexel stock

line number
GX75438 G15984

holding
☑

chromosome
0

Insertion site
AE003532 186848

crossGMRDCP-1

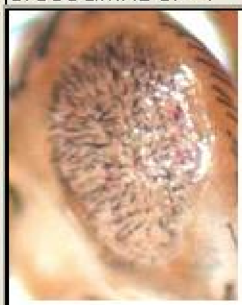

crossGMRGAL4

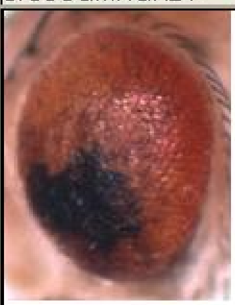

comments
pigment lost
CG6854 186848

similar phenotype

gene
CG6854

gene function
M : transcription factor, CTP synthase

B : Unknown

Number
281

source
Genexel stock

line number
GX75887 lost line

holding

chromosome
0

Insertion site

crossGMRDCP-1

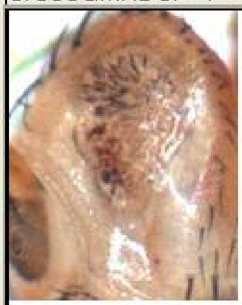

crossGMRGAL4

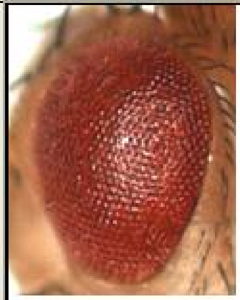

comments
small, pigment lost

similar phenotype

gene

gene function

Number	source
282	Genexel stock

line number	holding
GX75759 G5287	☐

chromosome	Insertion site
0	

crossGMRDCP-1	crossGMRGAL4

comments

slenderized, red

similar phenotype

gene

inside the CG5977

gene function

spas

Number
295

source
Genexel stock

line number
GX78123 G15482

holding
☑

chromosome
0

Insertion site
AE003693 205886

crossGMRDCP-1

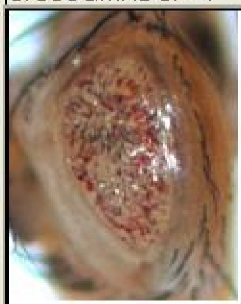

crossGMRGAL4

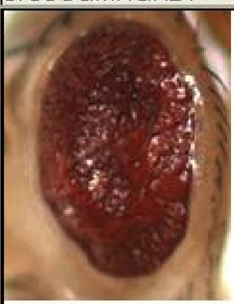

comments
small, glazed
CG31366 205886

similar phenotype

gene
CG31366=Hsp70Aa

gene function
hsp protein

Number	source
297	siganling stock

line number	holding
BL6287UAS-cdc42 V12#3	☐

chromosome	Insertion site

crossGMRDCP-1	crossGMRGAL4
Lethal	

comments

Lethal.all cyo

similar phenotype

gene

gene function

Number

298

source

siganling stock

line number

yw;UAS-InR wt#2

holding

☑

chromosome

0

Insertion site

crossGMRDCP-1

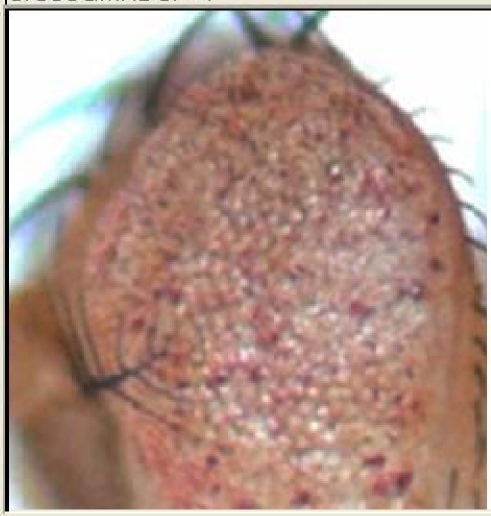

crossGMRGAL4

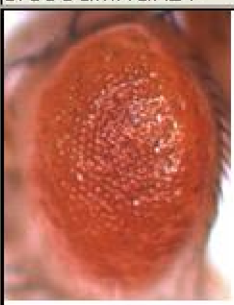

comments

bigger eye. Pigment lost.
gmrCross= bigger eye.little rough

similar phenotype

gene

InR(Insulin-like receptor)

gene function

M : insulin receptor, protein tyrosine kinase, insulin-like growth factor binding

B : insulin receptor signaling pathway

InR: insulin receptor, protein tyrosine kinase;

Number	source
302	Genexel stock

line number	holding
GX71056 G14526	☑

chromosome	Insertion site
0	AE003593 287653

crossGMRDCP-1

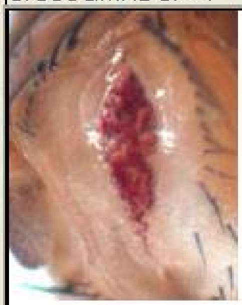

crossGMRGAL4

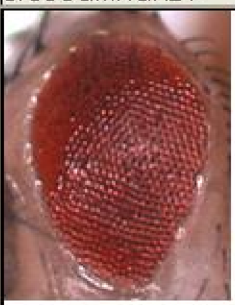

comments

slenderized, red
gmrgal4= slightly smaller eye.
Eip78c 287653

similar phenotype

gene

Ecdysone-induced protein 78C(Eip78c)

gene function

M : ligand-dependent nuclear receptor, specific RNA polymerase II transcription fact

B : larval/pupal development (sensu Insecta)

Number	source
303	Genexel stock

line number	holding
GX71057	☐

chromosome	Insertion site
0	

crossGMRDCP-1	crossGMRGAL4
	Normal eye

comments

pigment rescue
MKP3 167202
CG14081

similar phenotype

gene

Mkp3(Mitogen-activated protein kinase phosphatase 3)

gene function

M : protein tyrosine/serine/threonine phosphatase, MAP kinase phosphatase

B : protein amino acid phosphorylation

Number
304

source
Genexel stock

line number
GX71073

holding

chromosome
0

Insertion site

crossGMRDCP-1

crossGMRGAL4

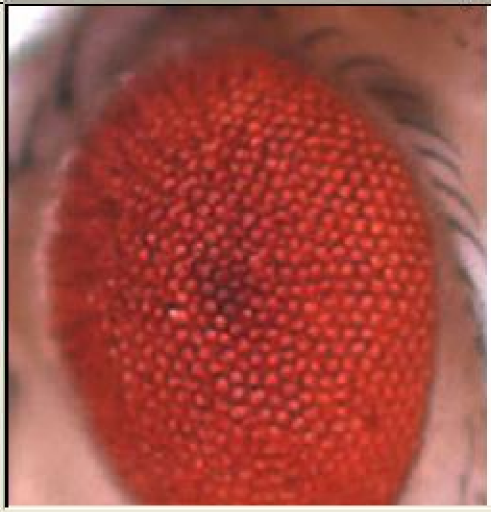

comments
ARGOS 179390
black spot,
gmrgal4 Cross= slightly rough

similar phenotype

gene
argos

gene function
M : Unknown

B : axon guidance, negative regulation of EGF receptor signaling pathway

Number	source
309	Genexel stock

line number	holding
GX46955	☐

chromosome	Insertion site
0	

crossGMRDCP-1	crossGMRGAL4

comments

slenderized, red

similar phenotype

gene

mekk

gene function

Number

319

source

Szeged stock

line number

EP(3)3354

holding

☑

chromosome

3

Insertion site

crossGMRDCP-1

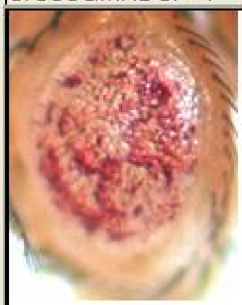

crossGMRGAL4

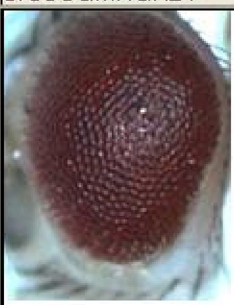

comments

, glazed- same as EP(2)0598(aop)
gmrgal4 cross = rough

similar phenotype

gene

jing interacting gene regulatory 1, jigr1

gene function

Number	source
330	Genexel stock

line number	holding
GX77224 G3142	☑

chromosome	Insertion site
0	

crossGMRDCP-1	crossGMRGAL4
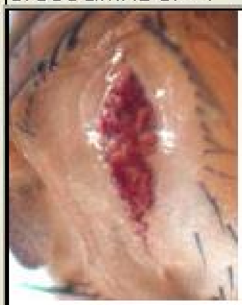	Normal eye

comments

slenderized, red

similar phenotype

gene

Sec61alpha

gene function

M : protein transporter

B : SRP-dependent, co-translational membrane targeting, translocation, cell death

Number	source
331	Genexel stock

line number	holding
GX77227 G16835	☑

chromosome	Insertion site
0	

crossGMRDCP-1	crossGMRGAL4
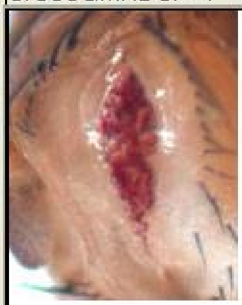	Normal eye

comments

slenderized, red

similar phenotype

gene

CG9813

gene function

Number

343

source

Genexel stock

line number

GX55961 G2198

holding

☑

chromosome

0

Insertion site

crossGMRDCP-1

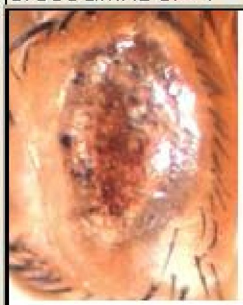

crossGMRGAL4

Normal eye

comments

small and flattened eye, black spots.

similar phenotype

gene

Hdc(headcase)

gene function

M : Unknown

B : cell differentiation, tracheal system development (a branching inhibitor produced specialised tracheal cells to prevent neighbouring cells from branching.)

Number

344

source

Genexel stock

line number

GX56814 G16886

holding

chromosome

0

Insertion site

crossGMRDCP-1

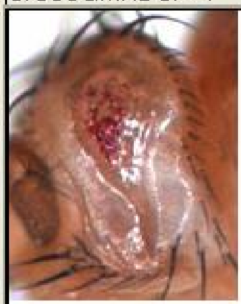

crossGMRGAL4

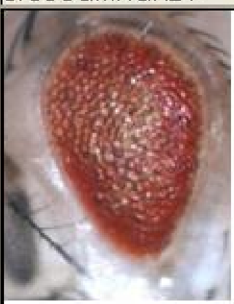

comments

slenderized, red

similar phenotype

gene

CG8789

gene function

M : protein serine/threonine kinase

B : protein amino acid phosphorylation

Number	source
345	Genexel stock

line number	holding
GX56709	☑

chromosome	Insertion site
0	

crossGMRDCP-1	crossGMRGAL4
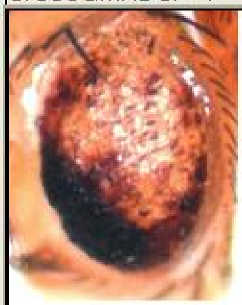	Normal eye

comments

severe rough and glazed eye, pigment accumulation?
l(2)05510

similar phenotype

gene

CG13432

gene function

lethal (2) 05510 l(2)05510

Number
346

source
Genexel stock

line number
GX57462 G15069

holding
☑

chromosome
0

Insertion site

crossGMRDCP-1

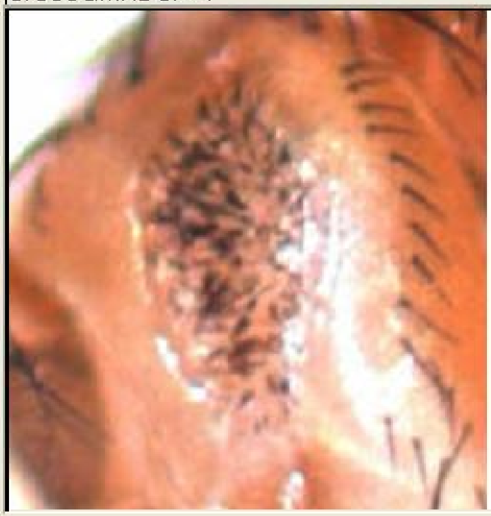

crossGMRGAL4

Normal eye

comments
flattened and crowded hair

similar phenotype

gene
effete(eff)

gene function
M : ubiquitin conjugating enzyme

B : ubiquitin cycle

Number	source
347	Genexel stock

line number	holding
GX55953 G4693	☑

chromosome	Insertion site
0	

crossGMRDCP-1	crossGMRGAL4
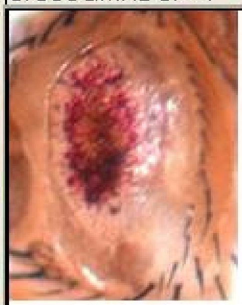	Normal eye

comments

flattened

similar phenotype

gene

CG17836

gene function

M : Unknown

B : Unknown

Number
350

source
Genexel stock

line number
GX47018 G3407

holding
☑

chromosome
0

Insertion site

crossGMRDCP-1

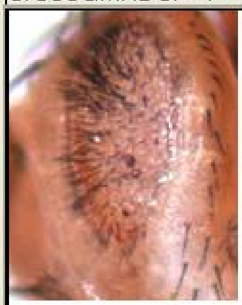

crossGMRGAL4

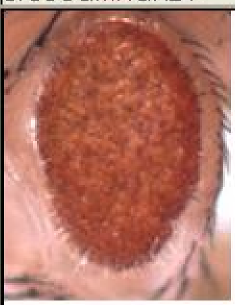

comments
small eye pigment lost

similar phenotype

gene
daughterless

gene function
M : transcriptional activator, specific RNA polymerase II transcription factor

B : sex determination, primary response to X:A ratio

Number
351

source
Genexel stock

line number
GX47057 G12355

holding

chromosome
0

Insertion site

crossGMRDCP-1
Lethal

crossGMRGAL4
Lethal

comments
pupa lethal
gmrgal4 cross= pupa lethal

similar phenotype

gene

gene function

Number	source
352	Genexel stock

line number	holding
GX47066 GE3467	☑

chromosome	Insertion site
0	

crossGMRDCP-1	crossGMRGAL4
Lethal	Normal eye

comments

pupa lethal

similar phenotype

gene

gene function

Number
353

source
Genexel stock

line number
GX47065 G15474

holding
☑

chromosome
3

Insertion site
AE003477 155819

crossGMRDCP-1

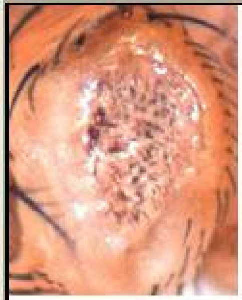

crossGMRGAL4

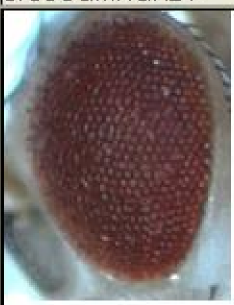

comments
small and pigment lost , second cross-> lethal
same as 40709
gmrcross-> rough eye

similar phenotype

gene
CG11505

gene function
M: Unknown

B : Unknown

RNA binding domain

Number
354

source
Genexel stock

line number
GX74265 G15347

holding
☑

chromosome
3

Insertion site
AE003523 89550

crossGMRDCP-1

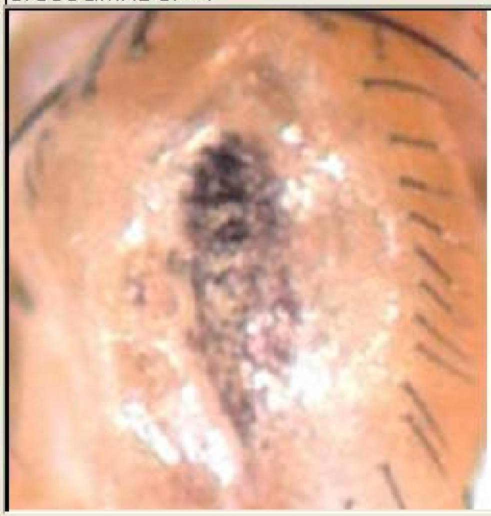

crossGMRGAL4

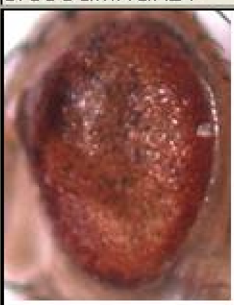

comments
slenderized, semilethal
gmrCross->rough,semilethal
inside the Eip74EF.but upstream of cds

similar phenotype

gene
Eip74EF(Ecdysone-induced protein 74EF)

gene function
M : specific RNA polymerase II transcription factor

B : autophagy

Number
385

source
Genexel stock

line number
GX22701

holding
☑

chromosome
2

Insertion site
AE003813 226447

crossGMRDCP-1

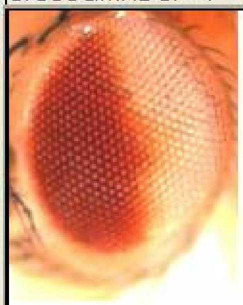

crossGMRGAL4

Normal eye

comments

HALF AND HALF CG12855 226447

similar phenotype

gene

hps

gene function

hps CG12855

Number
389

source
Genexel stock

line number
GX83464 G4511

holding
☑

chromosome
2

Insertion site

crossGMRDCP-1

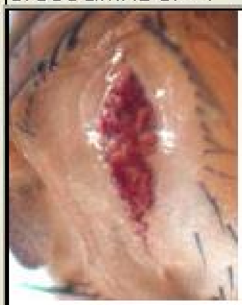

crossGMRGAL4

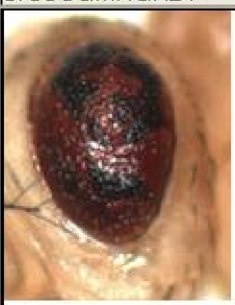

comments
slenderized, red
CG31217=CG10110(NCBI)

similar phenotype

gene
cleavage and polyadenylation specificity factor(cpsf)

gene function
cpsf
M : Mrna binding

B : Mrna cleavage, Mrna polyadenylation

Number
399

source
Genexel stock

line number
GX34104 G2194

holding
☑

chromosome
2

Insertion site
AE003824 163936

crossGMRDCP-1

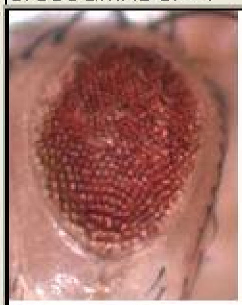

crossGMRGAL4

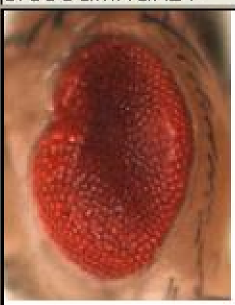

comments
small, round, glazed GX34104 165480, G2194 163936

similar phenotype

gene
Ef1alpha48D

gene function
M : translation elongation factor, protein-synthesizing GTPase, elongation

B : translational elongation

Number
401

source
Genexel stock

line number
GX34701 G12941

holding
☑

chromosome
2

Insertion site
AE003637 179164

crossGMRDCP-1

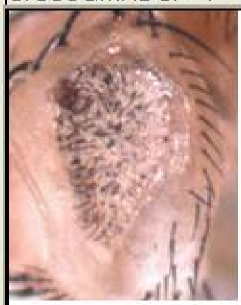

crossGMRGAL4

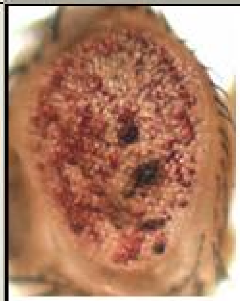

comments
slenderized, CG12283 179164

similar phenotype

gene
CG12283 KEK1

gene function
CG12283 kekkon-1
Its molecular function is described as: epidermal growth factor binding; transmembra
receptor protein serine/threonine kinase activity

Number

403

source

Genexel stock

line number

GX40709 G15474

holding

☑

chromosome

0

Insertion site

crossGMRDCP-1

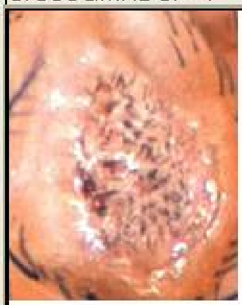

crossGMRGAL4

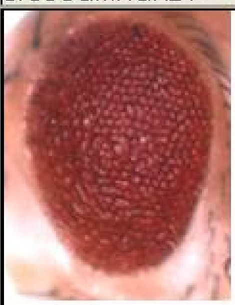

comments

small black hair in eye (74265) => second cross-> lethal
same position as 47065, same gmr, gD phenotype as 47065,
gmrCross-> rough

similar phenotype

gene

CG11505

gene function

M: Unknown

B : Unknown

RNA binding domain

Number

410

source

Genexel stock

line number

G7540

holding

☑

chromosome

0

Insertion site

crossGMRDCP-1

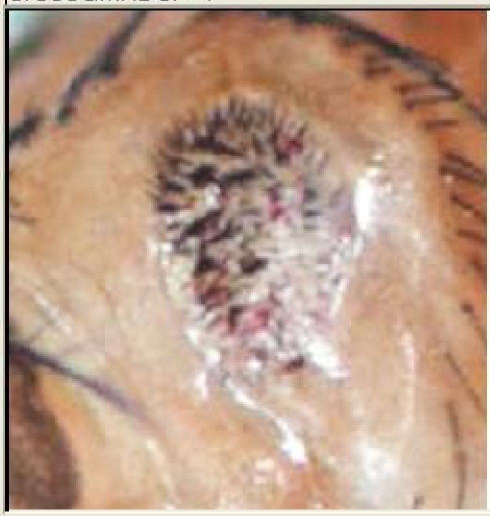

crossGMRGAL4

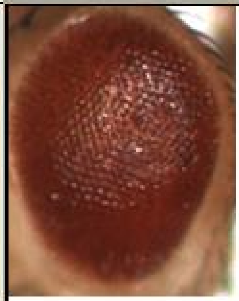

comments

small and black hair

similar phenotype

gene

pipsqueak(psq)

gene function

M : DNA binding

B : Unknown

Number	source
411	Genexel stock

line number	holding
BA8394	☑

chromosome	Insertion site
0	

crossGMRDCP-1	crossGMRGAL4
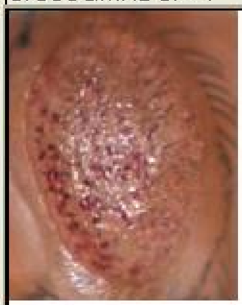	Normal eye

comments

, glazed CG32594

similar phenotype

gene

CG32594

gene function

CG32594

Number	source
412	Genexel stock

line number	holding
GX1512	☐

chromosome	Insertion site
0	

crossGMRDCP-1

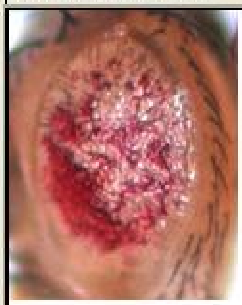

crossGMRGAL4

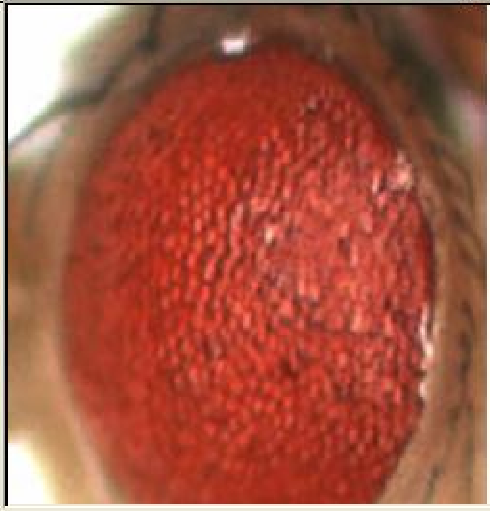

comments

small, glazed

similar phenotype

gene

gene function

Number	source
413	Genexel stock

line number	holding
GX6409	☑

chromosome	Insertion site
3	AE003733 205211

crossGMRDCP-1	crossGMRGAL4
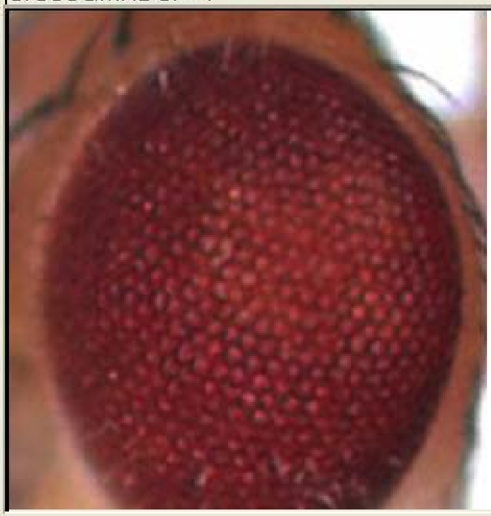	Normal eye

comments

RESCUED EYE, UNIQUE COLOR, SNF4Agamma 205211

similar phenotype

gene

SNF4Agamma(SNF4/AMP-activated protein kinase gamma subunit)

gene function

M : protein serine/threonine kinase, SNF1A/AMP-activated protein kinase

B : protein amino acid phosphorylation

Number

414

source

Genexel stock

line number

GX899

holding

chromosome

0

Insertion site

crossGMRDCP-1

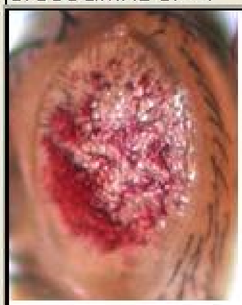

crossGMRGAL4

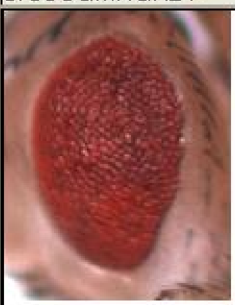

comments

same as GX1512

similar phenotype

gene

gene function

Number
416

source
Genexel stock

line number
G1851

holding
☑

chromosome
3

Insertion site
AE003519 269408

crossGMRDCP-1

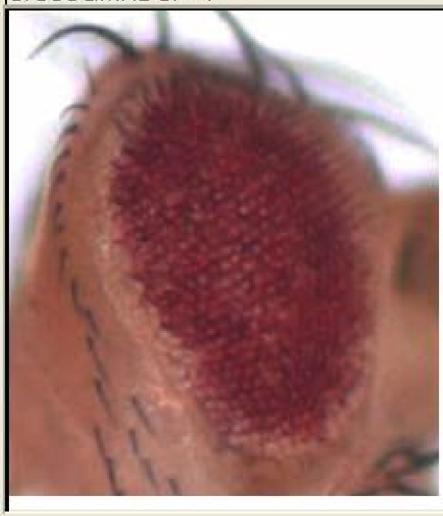

crossGMRGAL4
Normal eye

comments
CG3902 269408

similar phenotype

gene
Unknown

gene function
Unknown

Number	source
418	Genexel stock

line number	holding
G10174	☑

chromosome	Insertion site
1	AE003421 156746

crossGMRDCP-1	crossGMRGAL4
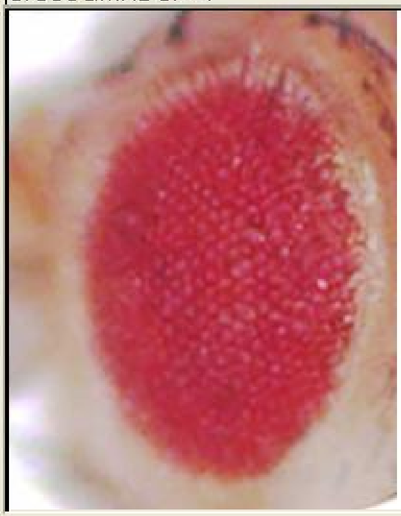	Normal eye

comments

br 156746

similar phenotype

gene

br

gene function

Number	source
419	Genexel stock

line number	holding
G2166	☑

chromosome	Insertion site
2	AE003799 315534

crossGMRDCP-1	crossGMRGAL4
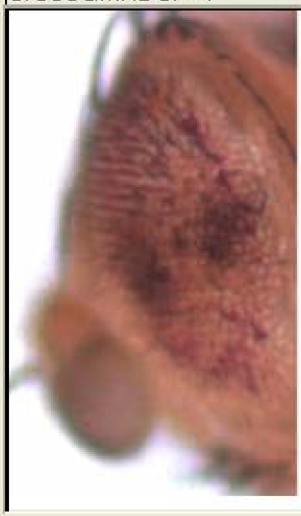	Normal eye

comments

eip55E 315534

similar phenotype

gene

eip55E

gene function

Number
420

source
Genexel stock

line number
G2362 bchs

holding
☑

chromosome
2

Insertion site
AE003611 211496

crossGMRDCP-1

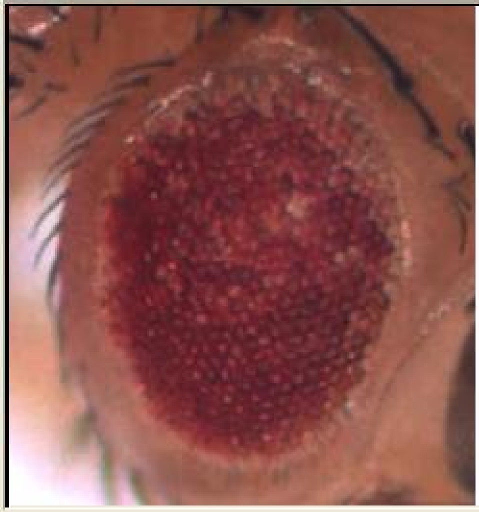

crossGMRGAL4

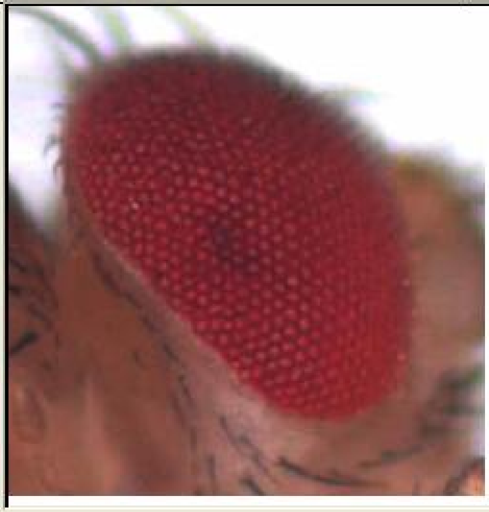

comments
BCHS SUPPRESOR? 211496

similar phenotype

gene
BCHS

gene function

Number
421

source
Genexel stock

line number
G12113

holding
☑

chromosome
2

Insertion site
AE003611 211693

crossGMRDCP-1

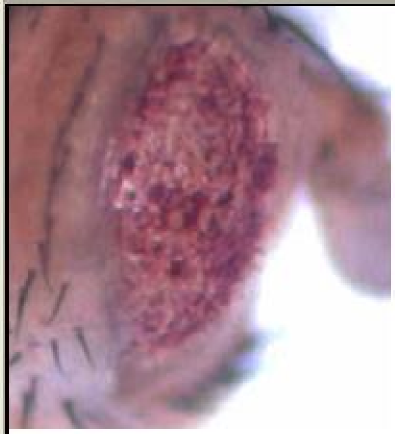

crossGMRGAL4

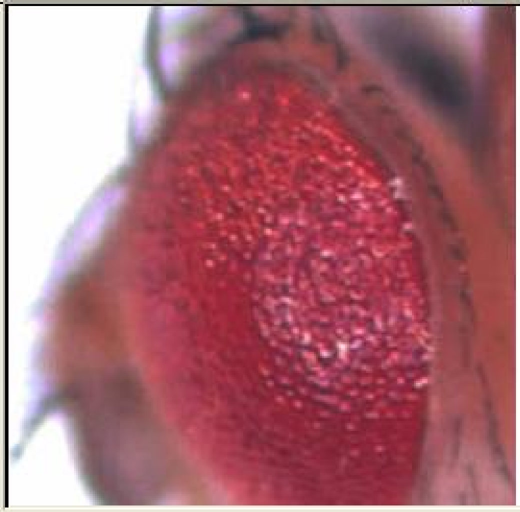

comments
bchs 211693

similar phenotype

gene
bchs

gene function
BCHS

Number
422

source
Genexel stock

line number
G13044

holding
☑

chromosome
0

Insertion site
AE003611 211951

crossGMRDCP-1

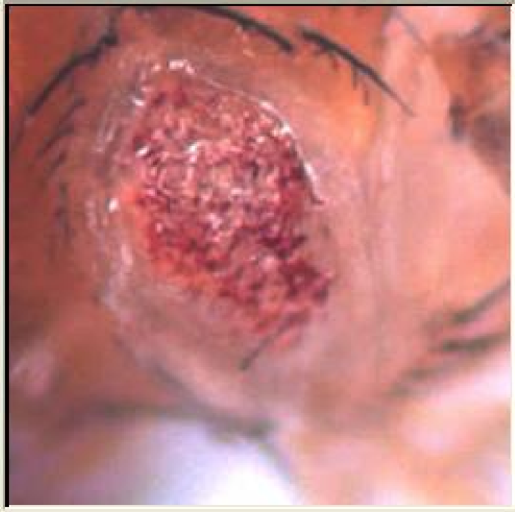

crossGMRGAL4

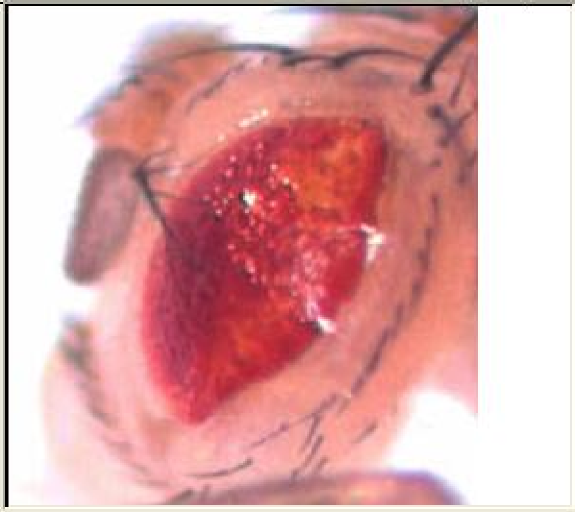

comments
bchs 211951

similar phenotype

gene
bchs

gene function

Number	source
423	Genexel stock

line number	holding
G3744	☑

chromosome	Insertion site
3	AE003556 135461

crossGMRDCP-1	crossGMRGAL4
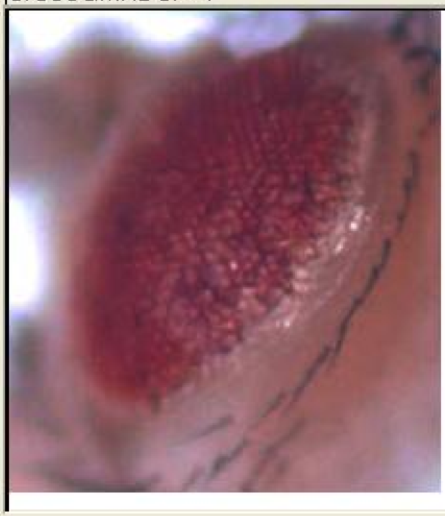	Normal eye

comments

ATG18 135461

similar phenotype

gene

ATG18

gene function

ATG18

Number	source
424	Genexel stock

line number	holding
G3772	☑

chromosome	Insertion site
3	AE003746 19136

crossGMRDCP-1	crossGMRGAL4
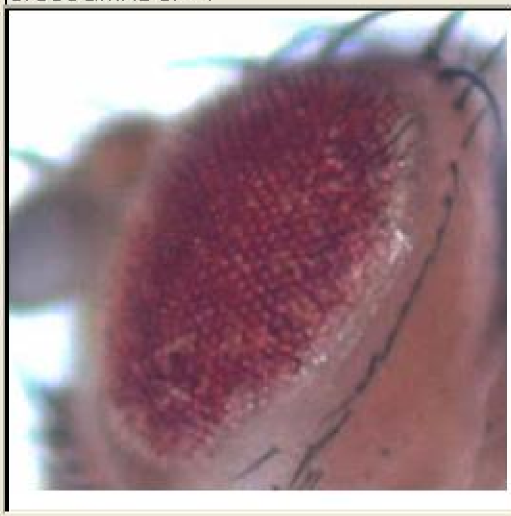	Normal eye

comments

ATG6 19136

similar phenotype

gene

ATG6

gene function

ATG6

Number	source
425	Genexel stock

line number	holding
G3894	☑

chromosome	Insertion site
3	AE003519 122740

crossGMRDCP-1	crossGMRGAL4
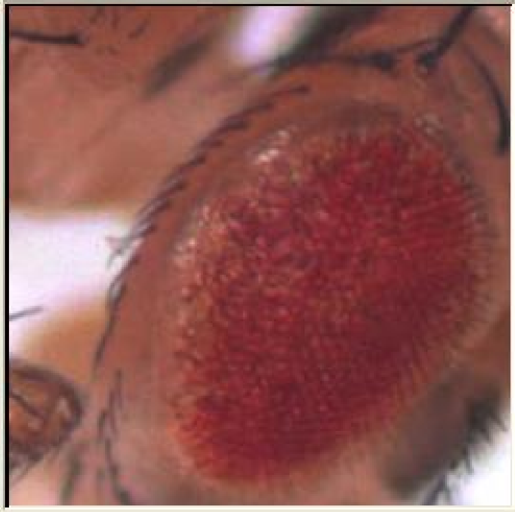	Normal eye

comments

AUT1 122740

similar phenotype

gene

AUT1

gene function

AUT1

Number

426

source

Genexel stock

line number

G4022

holding

☑

chromosome

3

Insertion site

AE003540 228430

crossGMRDCP-1

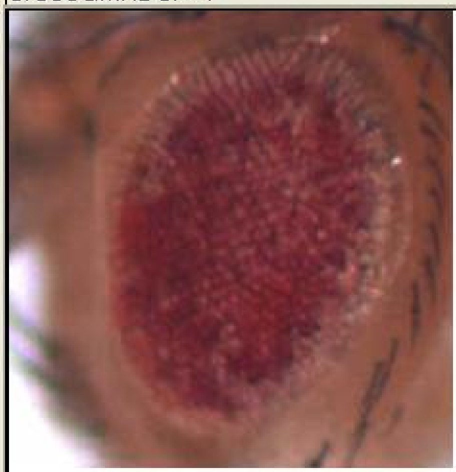

crossGMRGAL4

Normal eye

comments

ATG1 228430

similar phenotype

gene

ATG1

gene function

ATG1

Number
427

source
Genexel stock

line number
G13748

holding
☑

chromosome
3

Insertion site
AE003540 228529

crossGMRDCP-1

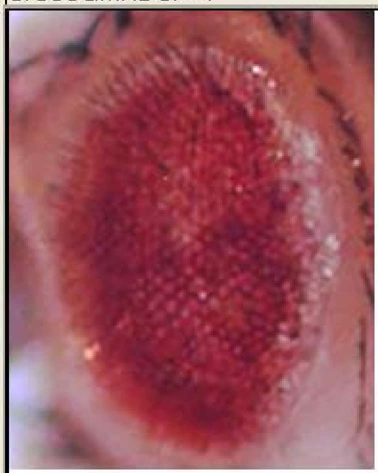

crossGMRGAL4
Normal eye

comments
ATG1 228529

similar phenotype

gene
ATG1

gene function
ATG1

Number
428

source
Genexel stock

line number
G6729

holding
☑

chromosome
3

Insertion site
AE003550 206457

crossGMRDCP-1

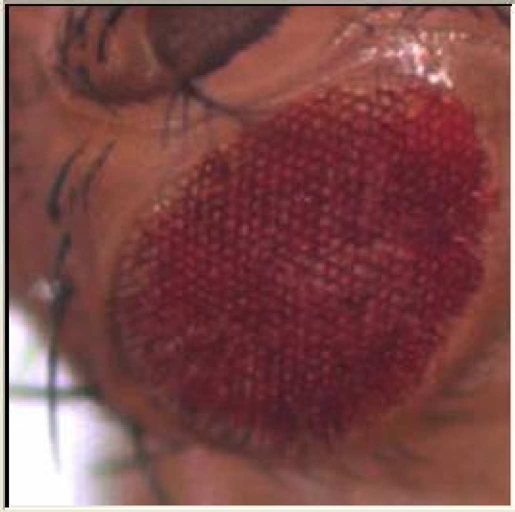

crossGMRGAL4

Normal eye

comments
CG6685 206457

similar phenotype

gene
CG6685

gene function
CG6685

Number
429

source
Genexel stock

line number
G4748

holding
☑

chromosome
3

Insertion site
AE003695 17769

crossGMRDCP-1

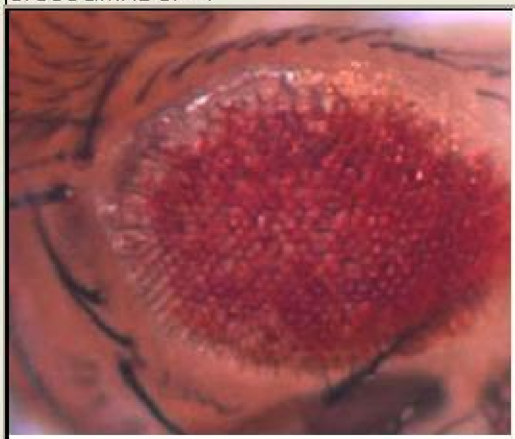

crossGMRGAL4

Normal eye

comments
CG4860 17769

similar phenotype

gene
CG4860

gene function
CG4860

Number	source
430	Genexel stock

line number	holding
G9085	☑

chromosome	Insertion site
3	AE003695 17523

crossGMRDCP-1	crossGMRGAL4
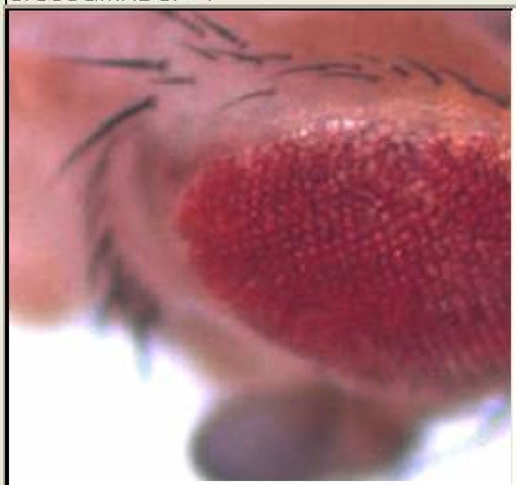	Normal eye

comments

CG4860 17523

similar phenotype

gene

CG4860

gene function

CG4860

Number
431

source
Genexel stock

line number
G14823

holding
☑

chromosome
3

Insertion site
AE003695 18204

crossGMRDCP-1

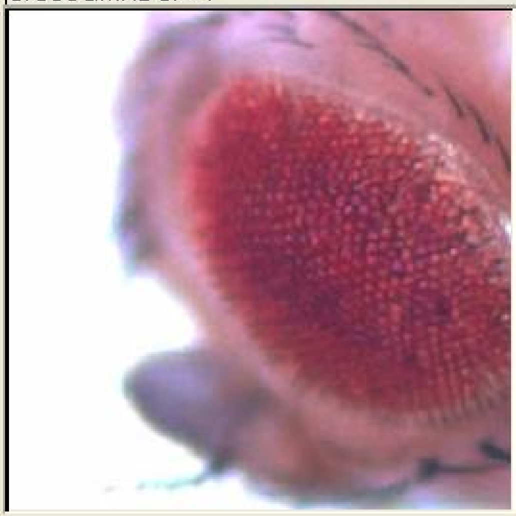

crossGMRGAL4

Normal eye

comments
CG4860 18204

similar phenotype

gene
CG4860

gene function
CG4860

Number
432

source
Genexel stock

line number
G6691

holding
☑

chromosome
3

Insertion site
AE003476 5754

crossGMRDCP-1

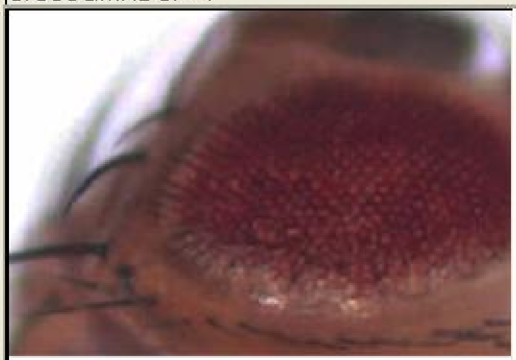

crossGMRGAL4

Normal eye

comments
ATG2 5754

similar phenotype

gene
ATG2

gene function
ATG2

Number	source
433	Genexel stock

line number	holding
G5566	☑

chromosome	Insertion site
3	AE003550 205542

crossGMRDCP-1	crossGMRGAL4
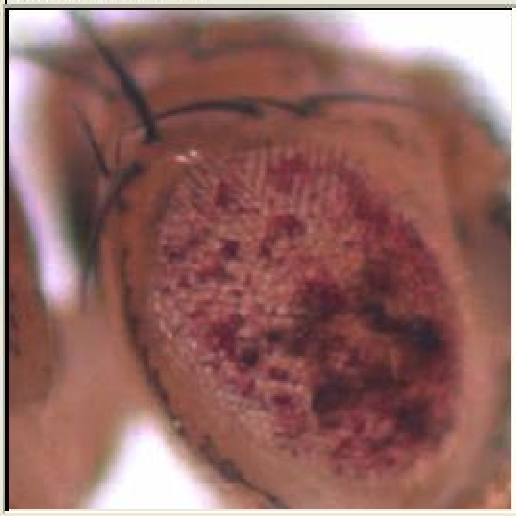	Normal eye

comments

NC DRONC 205542

similar phenotype

gene

NC DRONC

gene function

NC DRONC

Number
434

source
Genexel stock

line number
G5568

holding
☑

chromosome
3

Insertion site
AE003558 205542 239655

crossGMRDCP-1

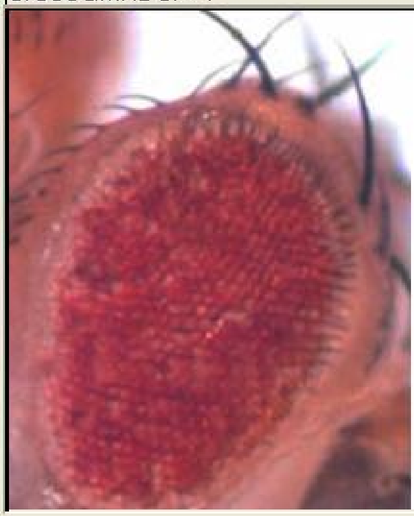

crossGMRGAL4

Normal eye

comments
CG12262 G5568 205542, G13743 239655

similar phenotype

gene
CG12262

gene function
CG12262

Number
435

source
Genexel stock

line number
G6854

holding
☑

chromosome
3

Insertion site
AE003746 19798

crossGMRDCP-1

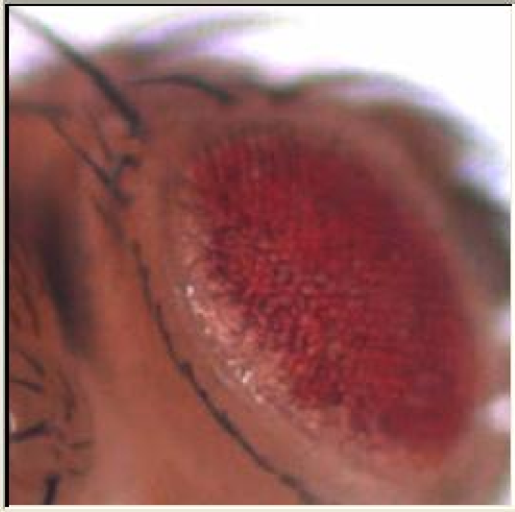

crossGMRGAL4

Normal eye

comments

CG5429 ATG6 G6854 19798

similar phenotype

gene

CG5429 ATG6

gene function

CG5429 ATG6

Number	source
436	Genexel stock

line number	holding
G8010	☑

chromosome	Insertion site
2	AE003799 309177

crossGMRDCP-1	crossGMRGAL4
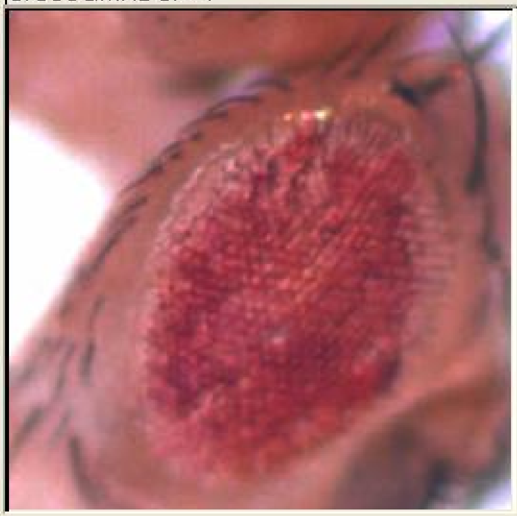	Normal eye

comments

CG5489 ATG7 G8010 309177

similar phenotype

gene

CG5489ATG7

gene function

CG5489ATG7

Number	source
437	Genexel stock

line number	holding
G8907	✔

chromosome	Insertion site
3	AE003530 64937

crossGMRDCP-1

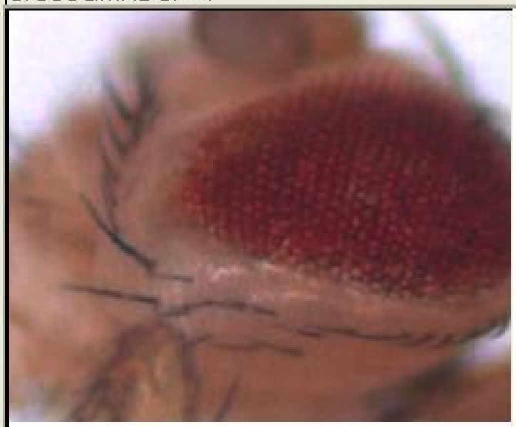

crossGMRGAL4

Normal eye

comments

CG7439 Argonaute 2 G8907 64937

similar phenotype

gene

Argonaute 2

gene function

Argonaute 2

Number	source
438	Genexel stock

line number	holding
G8940	☐

chromosome	Insertion site
3	AE003565 281188

crossGMRDCP-1	crossGMRGAL4

comments

S6K G8940 281188

similar phenotype

gene

S6K

gene function

S6K

Number	source
439	Genexel stock

line number	holding
G9749	☑

chromosome	Insertion site
1	AE003451 213709

crossGMRDCP-1	crossGMRGAL4
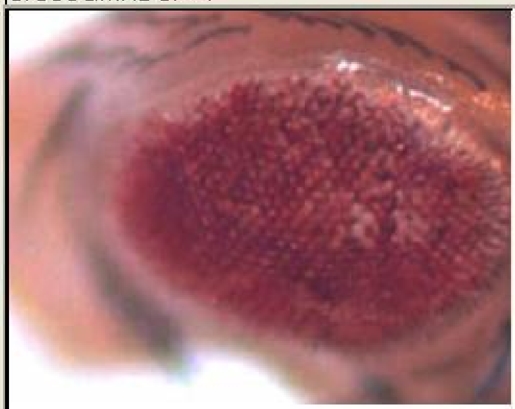	Normal eye

comments

CG32672 ATG8a G9749 213709

similar phenotype

gene

ATG8a

gene function

ATG8a

Number	source
440	Genexel stock

line number	holding
G12426	☑

chromosome	Insertion site
2	AE003669 211701

crossGMRDCP-1	crossGMRGAL4
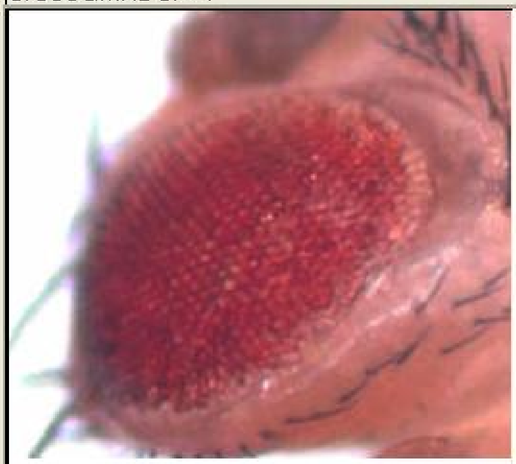	Normal eye

comments

CG8678 G12426 211701

similar phenotype

gene

CG8678

gene function

CG8678

Number

441

source

Genexel stock

line number

G13564

holding

☑

chromosome

2

Insertion site

AE003799 315472

crossGMRDCP-1

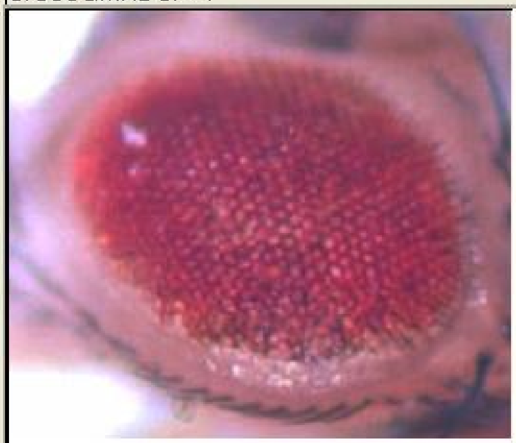

crossGMRGAL4

Normal eye

comments

Eip55E G13564 315472

similar phenotype

gene

Eip55E

gene function

Eip55E

Number
442

source
Genexel stock

line number
G18114

holding
☑

chromosome
0

Insertion site
AE003587 160706

crossGMRDCP-1

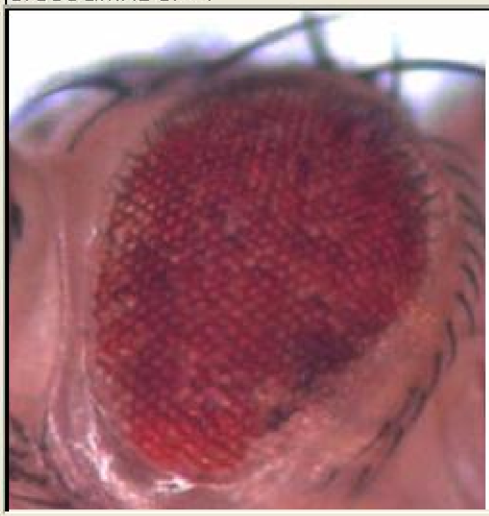

crossGMRGAL4

Normal eye

comments

CG4428 ATG4 G18114 160706

similar phenotype

gene

ATG4

gene function

ATG4

Number

443

source

Genexel stock

line number

G2481

holding

☑

chromosome

2

Insertion site

AE003799 315472

crossGMRDCP-1

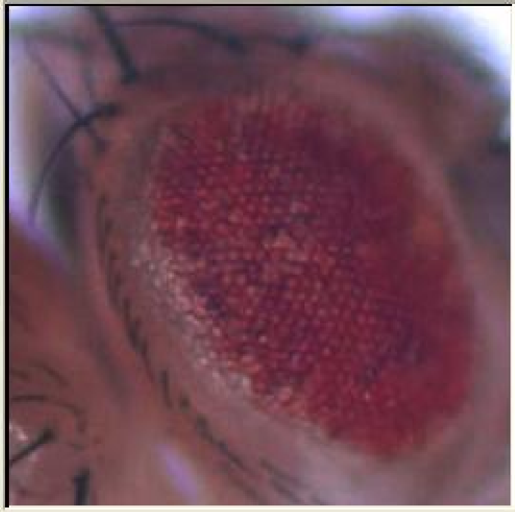

crossGMRGAL4

Normal eye

comments

CG5489 ATG7 G2481 315472

similar phenotype

gene

CG5489 ATG7

gene function

CG5489 ATG7

Number
444

source
Genexel stock

line number
G1972

holding
☑

chromosome
1

Insertion site
AE003421 152244

crossGMRDCP-1

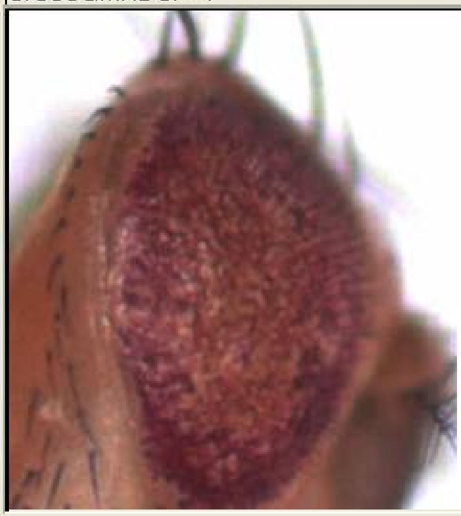

crossGMRGAL4

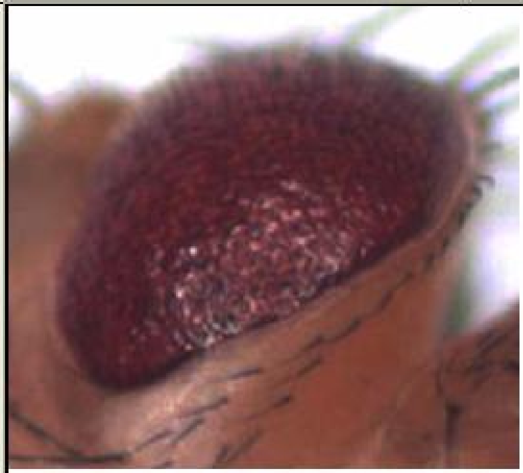

comments
BR G1972 152244

similar phenotype

gene
BR

gene function
BR

Number
445

source
Genexel stock

line number
G2628

holding
☑

chromosome
2

Insertion site
AE003576 53194

crossGMRDCP-1
LETHAL

crossGMRGAL4
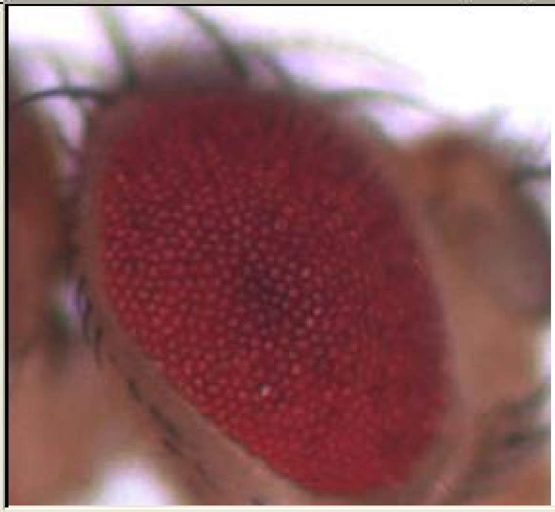

comments
Traf1 G2628 53194

similar phenotype

gene
Traf1

gene function
TNF-receptor-associated factor 1

Number
446

source
Genexel stock

line number
G9680

holding
☑

chromosome
0

Insertion site
AE003442 292211

crossGMRDCP-1

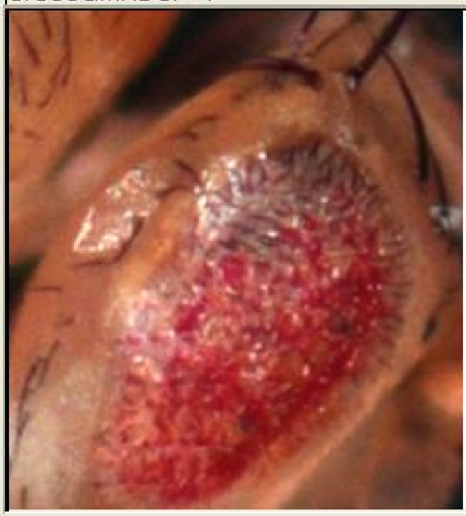

crossGMRGAL4

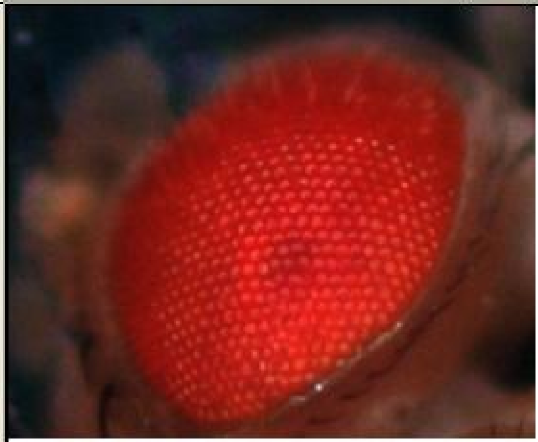

comments
Ubc-E2H G9680 292211

similar phenotype

gene
Ubc-E2H

gene function
Ubc-E2H

Number
447

source
Genexel stock

line number
G3534

holding
☑

chromosome
2

Insertion site
AE003832 87530

crossGMRDCP-1

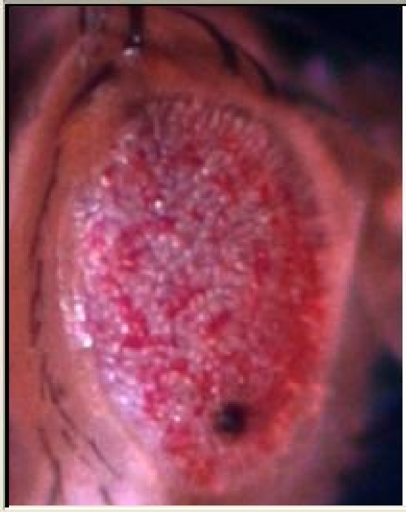

crossGMRGAL4

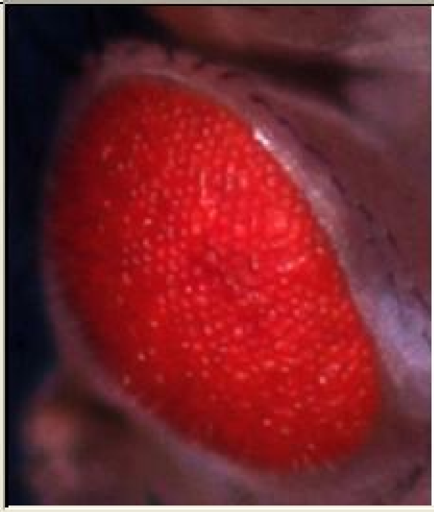

comments
Uba1 G3534 87530

similar phenotype

gene
Uba1

gene function
Uba1

Number
448

source
Genexel stock

line number
G6571

holding
☑

chromosome
0

Insertion site
AE003556 76828

crossGMRDCP-1

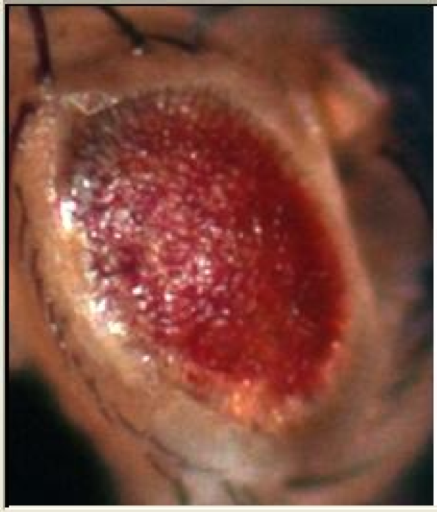

crossGMRGAL4

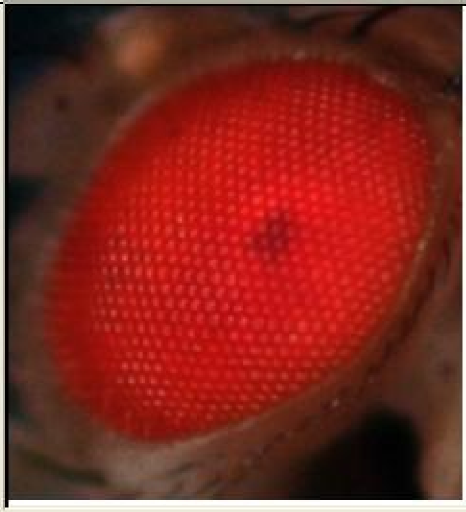

comments
CG7528 Uba2 G6571 76828

similar phenotype

gene
Uba2

gene function
Uba2

Number
449

source
Genexel stock

line number
G4384

holding
☑

chromosome
3

Insertion site
AE003556 76322

crossGMRDCP-1

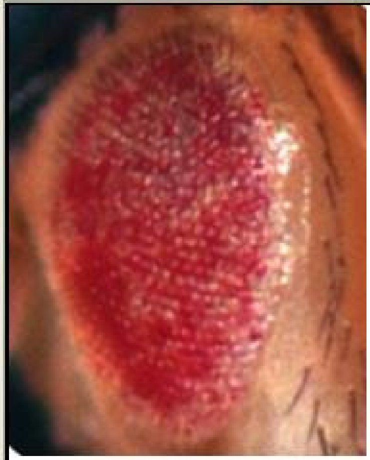

crossGMRGAL4

Normal eye

comments

CG7528 Uba2 G4384 76322

similar phenotype

gene

Uba2

gene function

Uba2

Number
450

source
Genexel stock

line number
G4874

holding
☑

chromosome
3

Insertion site
AE003551 221404

crossGMRDCP-1

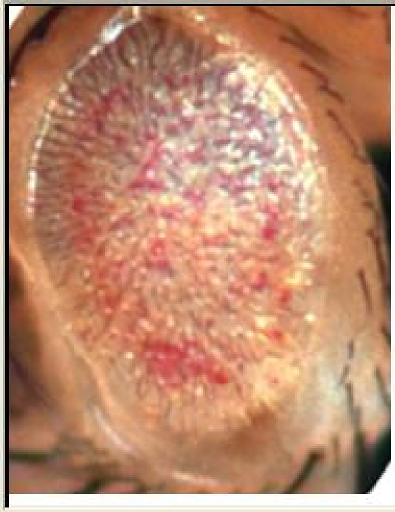

crossGMRGAL4
NORMAL

comments
CG8284 UBCD4 G4874 221404

similar phenotype

gene
UBCD4

gene function
UBCD4

Number	source
451	Genexel stock

line number	holding
G5032	☑

chromosome	Insertion site
3	AE003565 222801

crossGMRDCP-1	crossGMRGAL4
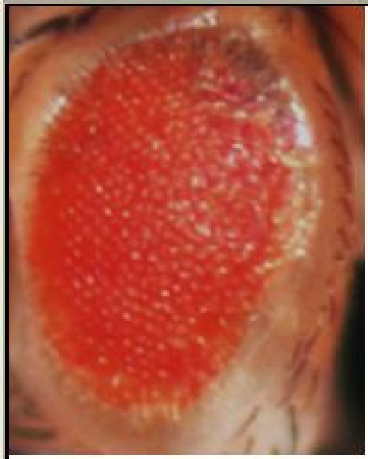	Normal eye

comments

UBP64E G5032 222801

similar phenotype

gene

UBP64E

gene function

UBP64E

Number
452

source
Genexel stock

line number
G5401

holding
☑

chromosome
3

Insertion site
AE003565 224699

crossGMRDCP-1

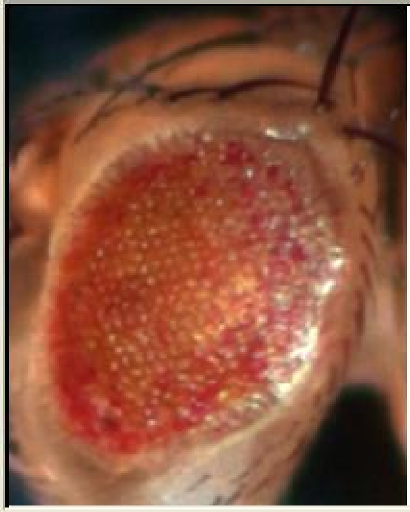

crossGMRGAL4
Normal eye

comments
UBP64E G5401 224699

similar phenotype

gene
UBP64E

gene function
UBP64E

Number	source
453	Genexel stock

line number	holding
G8583	☑

chromosome	Insertion site
3	AE003556 77421

crossGMRDCP-1

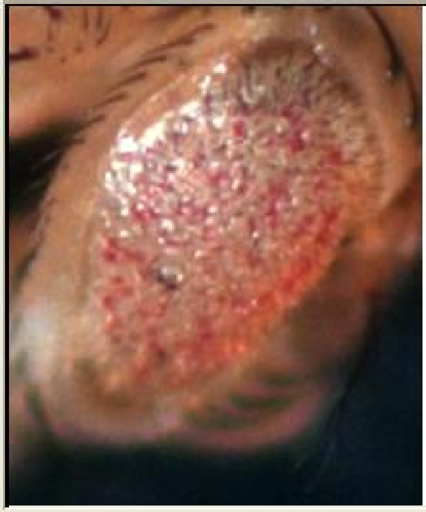

crossGMRGAL4

Normal eye

comments

UBA2 G8583 77421

similar phenotype

gene

UBA2

gene function

UBA2

Number	source
454	Genexel stock

line number	holding
G16806	☑

chromosome	Insertion site
3	AE003565 223636

crossGMRDCP-1	crossGMRGAL4

comments

Ubp64E G16806 223636

similar phenotype

gene

Ubp64E

gene function

Ubp64E
